# Chimeric peptide-based radiopharmaceuticals for glioblastoma imaging and therapy by targeting mHsp70 and enhancing BBB penetration

**DOI:** 10.1039/d6sc00011h

**Published:** 2026-06-03

**Authors:** Franziska Schuderer, Rúben D. M. Silva, Catarina I. G. Pinto, Lena Koller, Stefan Stangl, Lurdes Gano, Marco Cavaco, Miguel A. R. Castanho, Filipa Mendes, Susanne Kossatz, João D. G. Correia, Angela Casini

**Affiliations:** a Chair of Medicinal and Bioinorganic Chemistry, Department of Chemistry, School of Natural Sciences, Technical University of Munich Lichtenbergstrasse 4 85748 Garching bei München Germany angela.casini@tum.de; b Centro de Ciências e Tecnologias Nucleares, Instituto Superior Técnico, Universidade de Lisboa, CTN, LRS Estrada Nacional 10 2695-066 Bobadela Portugal jgalamba@ctn.tecnico.ulisboa.pt fmendes@ctn.tecnico.ulisboa.pt; c Department of Nuclear Medicine, TUM University Hospital, Central Institute for Translational Cancer Research (TranslaTUM), School of Medicine and Health, Technical University of Munich 81675 Munich Germany s.kossatz@tum.de; d Departamento de Engenharia e Ciências Nucleares, Instituto Superior Técnico, Universidade de Lisboa, LRS Estrada Nacional 10 2695-066 Bobadela Portugal; e Fundação GIMM – Gulbenkian Institute for Molecular Medicine Avenida Professor Egas Moniz Lisboa 1649–028 Portugal; f Instituto de Bioquímica, Faculdade de Medicina, Universidade de Lisboa Avenida Professor Egas Moniz Lisboa 1649–028 Portugal

## Abstract

Glioblastoma (GBM) is the most aggressive form of malignant brain cancer. Here, we synthesized two chimeric peptide-based radiopharmaceuticals (Comb-1 and Comb-2) targeted to the membrane-bound form of heat shock protein 70 (mHsp70), which is overexpressed in GBM tissues, for future theranostic applications. The design concept features a DOTA chelator for coordination to different radiometals tethered directly or *via* a PEG linker to a chimeric peptide. The latter combines the Hsp70-targeting ability of the TPP sequence with the blood–brain barrier (BBB) penetration of another 7-amino acid sequence (d-PepH3) derived from the dengue virus capsid protein (DEN2C). The two compounds were successfully radiolabelled with gallium-67, suitable for single photon emission computed tomography (SPECT) imaging, or with lutetium-177 for β^−^ therapy. Furthermore, the *in vitro* properties of the ligand, including lipophilicity (log *D*_7.4_), human serum albumin (HSA) binding, and stability in human serum were evaluated. The presence of the TPP sequence affected the half-life of the combinatorial peptides. Cytometry assays performed with a fluorescent analogue of Comb-2 confirmed the binding to mHsp70-expressing U87-MG cells. In an *in vitro* model, all tracers demonstrated the ability to cross the BBB, indicating that conjugation of the mHsp70-targeting peptide to the PepH3 sequence did not impair its translocating properties. Biodistribution experiments with ^67^Ga-labeled compounds were performed in naive female CD1 mice and showed brain uptake at 2 min p.i., as well as renal excretion. For the best performing compound [^67^Ga]Ga-Comb-2 (0.60 ± 0.17% IA g^−1^ (injected activity per gram)), the biodistribution experiment was also performed with perfusion of the organs after sacrifice, and the results showed retention of radioactivity in the brain (0.14 ± 0.05% IA g^−1^). Further metabolic studies in murine urine and blood were performed after biodistribution, confirming the stability of the chimeric tracers.

## Abstract 2

Glioblastoma (GBM) is the most aggressive form of malignant brain cancer. We designed chimeric peptide-based radioligands that cross the blood-brain barrier and target GMB.

## Graphical abstract

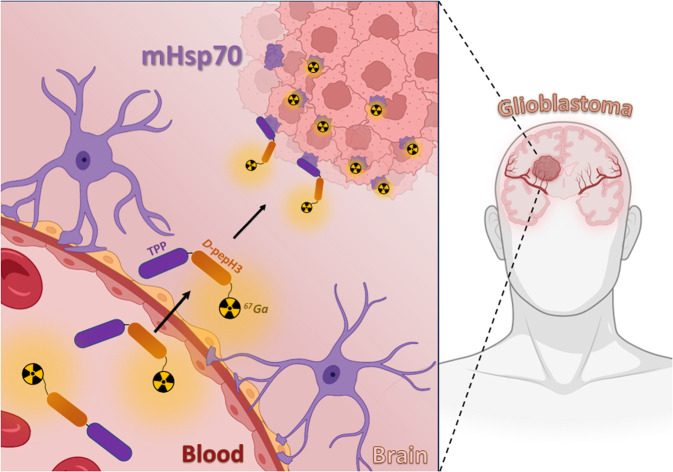

## Introduction

The most aggressive form of malignant brain cancer is glioblastoma (GBM).^1–3^ Standard treatment options include maximal resection, followed by external beam radiation therapy, chemotherapy with temozolomide, or a combination of both.^2,3^ However, for most patients, these are just palliative treatments that offer no cure. Considering this unfavorable prognosis and a very poor median overall survival of less than 2 years from diagnosis, new imaging and treatment options are urgently needed.^2,3^ Molecular imaging and targeted radionuclide therapy (TRT) represent valuable tools to address that goal. TRT with high-affinity ligands that exhibit high selectivity for the target can result in minimal negative impact on healthy tissue. This can be a crucial advantage for aggressive tumors like GBM, which is known for its resistance to standard therapies due to high tumor heterogeneity.^4^ To date, various approaches to radionuclide therapy for gliomas are in the early stages of development, targeting classical receptors such as the somatostatin receptor (SSTR) type 2, the prostate-specific membrane antigen (PSMA), and the chemokine receptor CXCR4, among others.^5–9^ The majority of the targeting agents are based on monoclonal antibodies,^10–12^ while some are also peptide-based compounds.^13,14^ Ongoing Phase I and I/II clinical trials are targeting the large neutral amino acid transporter 1 (LAT1, [^131^I]I-TLX101),^15^ SSTR2 ([^177^Lu]Lu-DOTA-TATE),^16^ gastrin-releasing peptide receptor (GRPR) ([^177^Lu]Lu-NeoB),^17^ and carbonic anhydrase XII ([^177^Lu]Lu-6A10 Fab-fragments).^18^ In this context, new radiolabeled peptides specifically designed to address cancer-specific targets expressed in GBM cells could prove highly beneficial.

One of the possible targets in GBM is the membrane-bound form of the heat shock protein 70 (mHsp70).^19^ The physiological functions of the intracellular form Hsp70, a molecular chaperone expressed ubiquitously in healthy and cancer cells in response to stress, include the folding and refolding of proteins to enable the (re-)gain of their functionality, as well as their targeting for degradation by the proteasome.^20^ Hsp70 is overexpressed in a variety of different cancers, increasing their resistance to several stress factors (*e.g.* hypoxia) and to apoptosis, and its overexpression can often be correlated with elevated tumor growth and cell migration, leading to poor prognosis.^20,21^ In cancer cells, cytosolic Hsp70 can be transported to the nucleus, translocated to the cytoplasmic membrane (mHsp70), or even be transported to the extracellular space.^20,22,23^ Screening of thousands of patient samples of several cancer types revealed that about 50% of the samples were positive for mHsp70, while control samples of healthy tissue were negative.^23–31^ This makes mHsp70 a potential target for tumor imaging and therapy.^24^ Moreover, Lobinger *et al.* stained 37 samples from glioma patients with different WHO grades and showed that all samples were positive for mHsp70, whereas the healthy brain tissue controls were negative.^32^

**Fig. 1:**
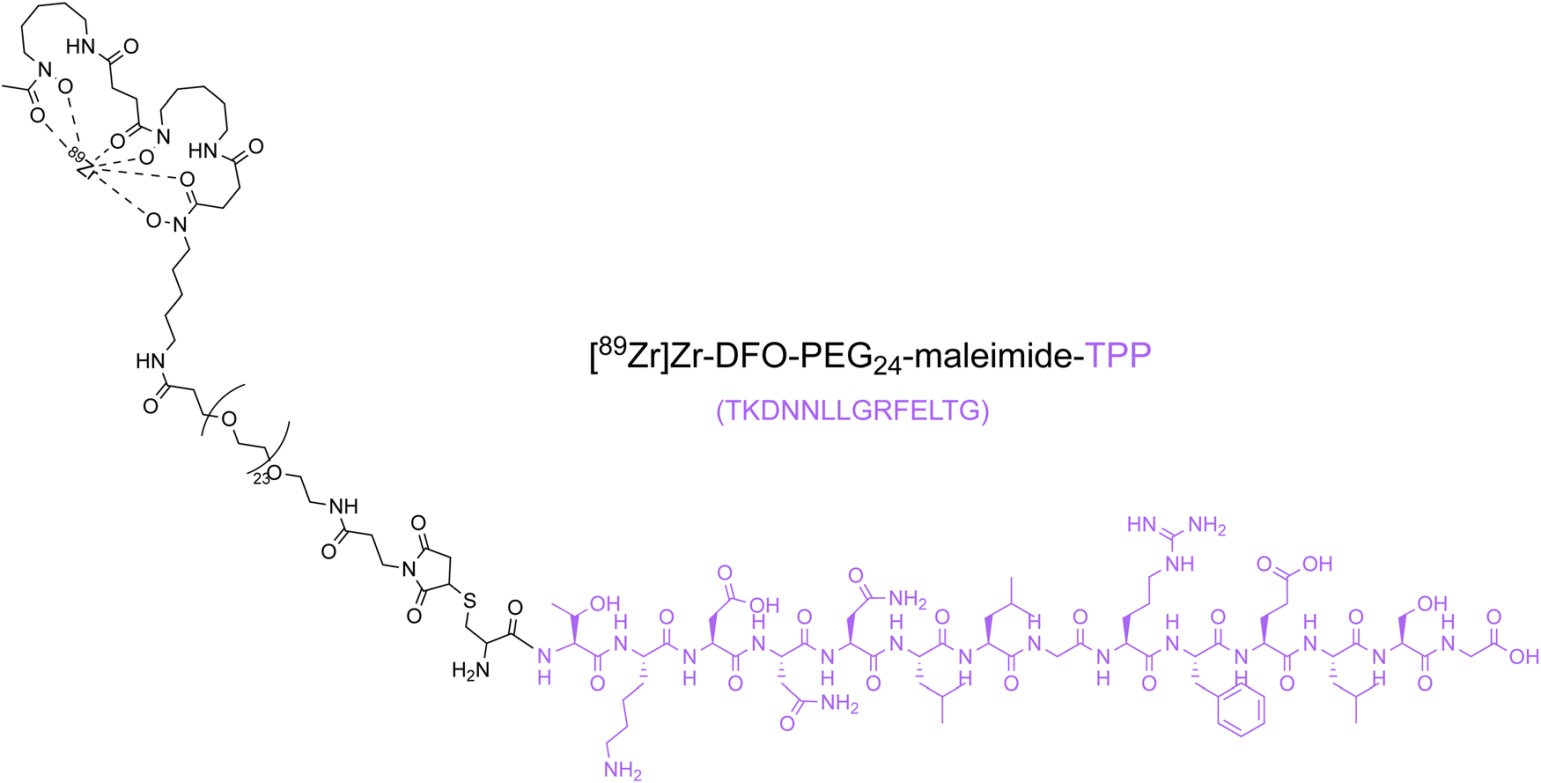
Previously reported structure of a ^89^Zr-labelled radiotracer featuring the TPP targeting mHsp70.^37^

Notably, Multhoff and coworkers identified both an antibody (cmHsp70.1) and a tumor penetrating peptide (TPP, previously TKD) targeting mHsp70.^24,33,34^ The latter is a 14-mer peptide sequence, derived from the extracellular oligomerization domain of mHsp70, TKDNNLLGRFELSG (TPP, 450–463 AA), which was first reported to have the same ability to activate natural killer (NK) cells, most likely *via* binding to mHsp70.^33,35^ Further *in vitro* studies revealed that carboxyfluorescein(CF)-labeled TPP exhibited specific binding to different mHsp70-positive tumor cells and fast internalization in mHsp70-positive breast cancer cells.^24^ Biodistribution studies in several mHsp70-positive tumor-bearing mice revealed tumor accumulation of CF-labeled TPP, with the kidneys being the only organ with distinct off-target accumulation, suggesting renal excretion.^36^ Most importantly, the highly specific accumulation of TPP together with the exclusive expression of mHsp70 in tumor cells, has already been exploited in the development of radiotracers, including the positron emission tomography (PET) tracer [^89^Zr]Zr-DFO-PEG_24_-TPP (Fig. 1),^37^ which showed specific mHsp70-expression-dependent tumor accumulation in mouse models bearing subcutaneous breast (4T1) or colorectal (CT26) tumors.^37^

One major challenge in delivering drugs to the brain is crossing the blood–brain barrier (BBB). The BBB is a tight cell layer that controls the influx of all substances from the bloodstream into the brain parenchyma, making the delivery of drugs (including radiopharmaceuticals) to the brain a major challenge.^38–40^ Various strategies have been employed to address this major issue, utilizing both passive and mediated mechanisms for permeation across the BBB.^41–44^ Among the latter, the use of BBB shuttles constitutes an elegant strategy for targeting the brain, including receptor-mediated transcytosis (RMT), carrier-mediated transcytosis (CMT), and adsorptive-mediated transcytosis (AMT).^45–47^ In this context, chimeric cell-penetrating peptides (CPPs) are engineered molecules that combine sequences from two or more different peptides to create a novel agent with enhanced or combined functions.^48^ Chimeric CPPs can aid the transportation of drugs (including tumor-targeting peptides) that are unable to pass the BBB by conjugating them to a brain-targeting peptidic vector.^48,49^

**Scheme 1:**
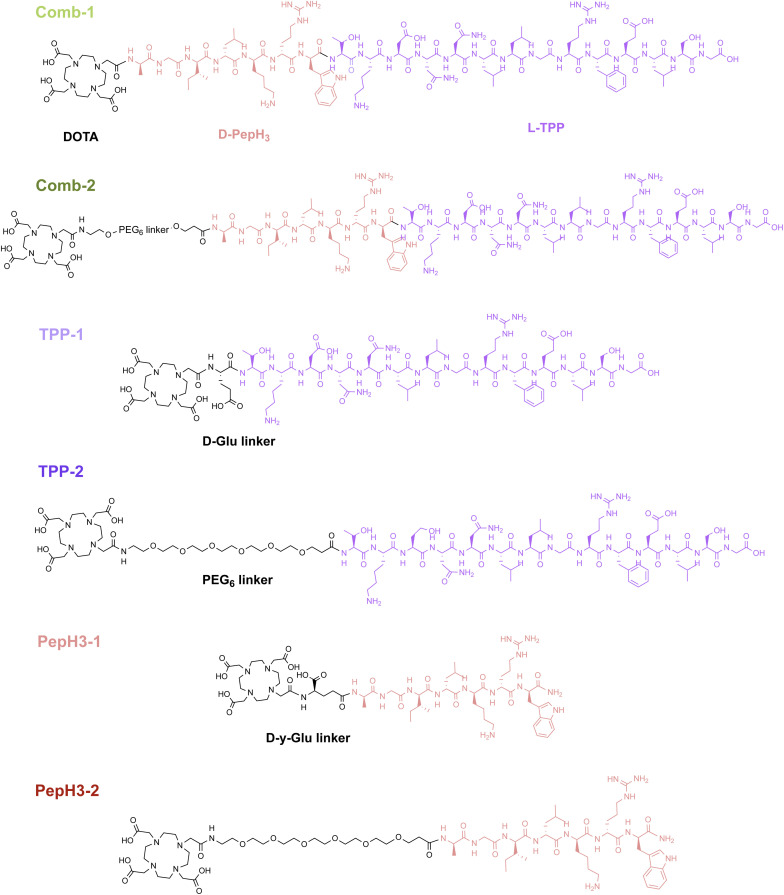
Structures of the chimeric compounds Comb-1 and Comb-2 reported in this work, combining the mHsp70-targeting abilities of TPP with the BBB-penetration of d-PepH3, and of the control tracers featuring only one of the two peptidic domains and with/without the PEG_6_ linker.

Despite the intensive ongoing research, there is still a lack of such peptide-based tracers suitable for the TRT of GBM that combine, simultaneously, the ability to cross the BBB and target GBM cells.^45^ This becomes evident in the aforementioned TRT trials of GBM patients, where the peptide derivatives are delivered *via* intratumoral/intracavitary injection. Therefore, in this work, we designed chimeric radiotracers that combine the Hsp70-targeting ability of the TPP sequence with the BBB penetration of another 7-amino acid sequence, named PepH3 (Scheme 1). PepH3 is derived from AA 63–69 of the 100 AA-long dengue virus capsid protein (DEN2C) and it is able to cross the BBB *via* AMT, while also featuring high *in vivo* stability.^50,51^ It should be noted that in our work, to improve the stability of the PepH3 peptide in human serum and *in vivo*, we opted for the unnatural d-PepH3 sequence.^52^ Importantly, our radiotracers featured a chelator for labeling with different radiometals, suitable for PET (*e.g.* Ga-68) or single photon emission computed tomography (SPECT) imaging (*e.g.* Ga-67), as well as therapy (*e.g.* Lu-177). Furthermore, the properties of gallium-67 and lutetium-177 labelled compounds, including lipophilicity determination, human serum albumin binding (HSA), and stability in human serum, were assessed *in vitro*. The binding of one of the chimeric compounds labeled with the luminescent fluorescein 5-isothiocyanate (FITC) to the mHsp70 protein was evaluated by cytometry in human glioblastoma U87-MG cells. Moreover, the ability of the radiotracers to translocate across an *in vitro* BBB cellular model and to accumulate in the brain of healthy, naive CD1 mice was also investigated. Metabolism studies complemented the *ex vivo* biodistribution and provided important information on the *in vivo* stability of the compounds.

## Results and discussion

### Synthesis and characterization of the peptide conjugates

With the goal of combining the mHsp70-targeting ability of TPP and the BBB-penetration of d-PepH3 into one radiotracer, the combinatorial compounds Comb-1 (DOTA-d-PepH3-TPP) and Comb-2 (DOTA-PEG_6_-d-PepH3-TPP) (Scheme 1) were synthesized *via* manual and automated solid-phase peptide synthesis (SPPS). In our nomenclature, the number 2 always indicates the use of a PEG_6_ linker, while the compounds with the number 1 contain either a shorter linker version (based on glutamate, as in compounds TPP-1 and PepH3-1) or no linker at all (Comb-1). The design of all TPP-containing compounds included the TPP sequence at the C-terminus of the construct, since modifications at the N-terminus of TPP are known to be tolerated and do not interfere with the affinity for mHsp70.^36,37^ In both combinatorial compounds, the d-PepH3 sequence was incorporated in a bridging fashion between the DOTA (or DOTA-PEG) moiety and the TPP. This design considered that d-PepH3 would most likely retain its BBB-translocation capabilities even when incorporated into a more complex peptide sequence, as it is derived from the central part of the DEN2C sequence.^50^ In fact, we also performed an *in silico* assessment of the BBB-translocation capabilities of the peptides using a predictive method that relies on the peptides' physicochemical properties (Table S1).^53^ Our results suggested that PepH3-1 and PepH3-2 have a high translocation capability (translocation score >0.80), followed by the chimeric tracers Combo-1 and Combo-2, whereas TPP-1 and TPP-2 exhibit poor translocation capability (Table S1).

Aimed at discerning the contribution of each peptidic building block, four other compounds (PepH3-1, PepH3-2, TPP-1, and TPP-2, see Scheme 1), containing only one of the two peptide sequences and with/without the PEG linker, were synthesized following the same procedure.

**Fig. 2:**
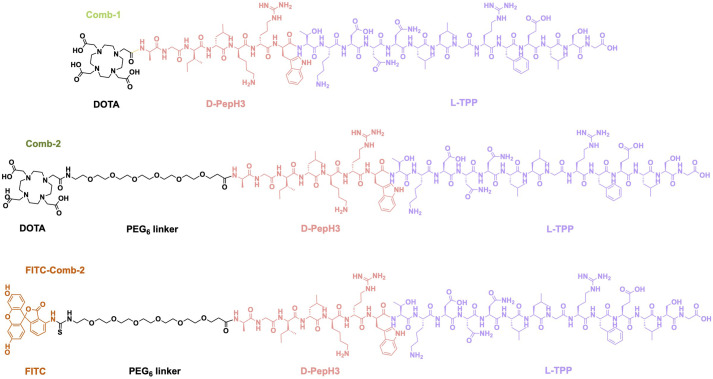
Structures of the fluorescent compounds FITC-Comb-2 and FITC-TPP-2 reported in this work.

Additionally, fluorescent derivatives (FITC-TPP-2 and FITC-Comb-2, Fig. 2) were designed by exchanging the DOTA chelator with fluorescein isothiocyanate (FITC) to be used in flow cytometry analysis. We chose FITC as a fluorophore because it is a bright, widely used, and well-established tag that has been used in previous studies.^37^

After synthesis and purification (≥95%) by Reversed-Phase High-Performance Liquid Chromatography (RP-HPLC), all peptide conjugates were characterized by High-Resolution Electrospray Ionization Mass Spectrometry (HR-ESI-MS) (Fig. S1–S36). The compounds containing the DOTA chelator were labeled with [^67^Ga]Ga or [^177^Lu]Lu. After optimizing the reaction conditions, specifically temperature and time, the corresponding radiopeptides were obtained with high radiochemical purity (≥95%) (Fig. S37–S49).

### *In vitro* evaluation of the compounds' lipophilicity, stability, serum albumin and target binding

**Fig. 3:**
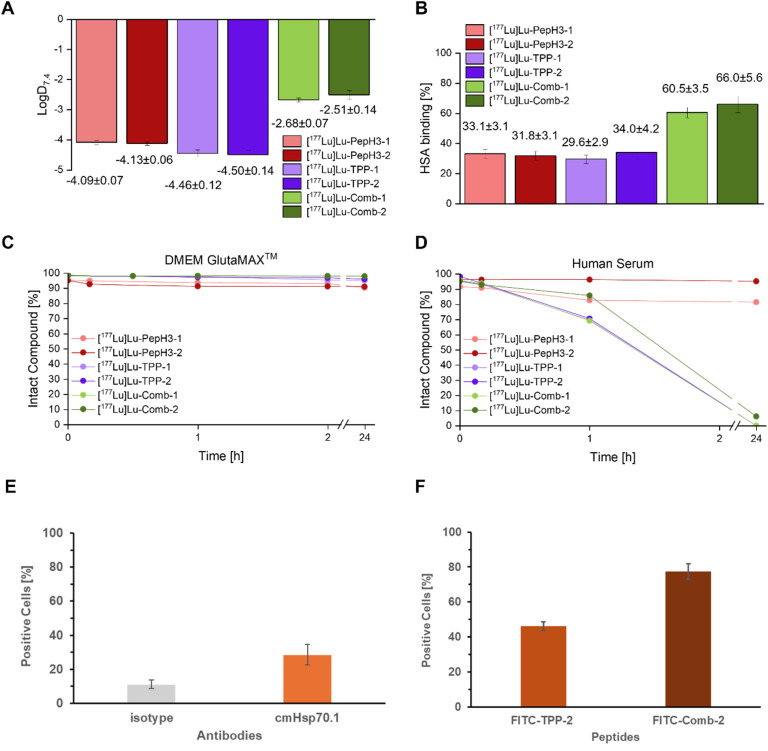
*In vitro* evaluation of the reported ^177^Lu-labeled radiotracers, including: (A) lipophilicity (log *D*_7.4_). (B) HSA binding, as well as stability in (C) cell culture medium and (D) human serum determined by radio RP-HPLC. (E) Cytometry analysis of mHsp70 expression in human glioblastoma U87-MG cells with antibody FITC-cmHsp70.1 (50 µg mL^−1^). AF488 (50 µg mL^−1^) was used as a negative isotype control. (F) Cytometry analysis of FITC-TPP-2 and FITC-Comb-2 to determine binding of the peptides (5 µm) to U87-MG cells. Both analyses (E) and (F) were performed with 30 min of incubation (37 °C, 5% CO_2_).

The hydro/lipophilic nature of all compounds was assessed through the determination of the octanol-PBS partition coefficient at pH = 7.4 (log *D*_7.4_) by the shake flask method. The results (Fig. 3A) show that all six compounds were hydrophilic with log *D*_7.4_ values in the same range as those of the reported radiolabeled benchmark compounds [^177^Lu]Lu-PSMA-617 (−4.44 ± 0.15) and [^68^Ga]Ga-DOTA-TATE (−2.81 ± 0.11).^54–57^ Nevertheless, [^177^Lu]Lu-Comb-1 and [^177^Lu]Lu-Comb-2 show higher lipophilicity with respect to the non-combinatorial derivatives, which might be beneficial for brain uptake.^58^

A balanced HSA-binding is one of the key parameters to achieve sufficient tracer bioavailability, as it influences both circulation time and tissue penetration.^54,59^ [^177^Lu]Lu-Comb-1 (60.5 ± 3.5%) and [^177^Lu]Lu-Comb-2 (66.0 ± 5.6%) showed a 1.8- to 2.2-fold increase in HSA-binding in comparison to the non-combinatorial compounds [^177^Lu]Lu-PepH3-1, [^177^Lu]Lu-PepH3-2, [^177^Lu]Lu-TPP-1, and [^177^Lu]Lu-TPP-2 (29.6 ± 2.9% – 34.0 ± 4.2%) (Fig. 3B). All six peptides showed high stability in medium, with more than 90% intact compound after 24 h of incubation (Fig. 3C, Table S2 and Fig. S50).

Further *in vitro* stability studies were performed in human serum (Fig. 3D, Table S3, and Fig. S51). All compounds remained >90% intact after 10 min at 37 °C, and the first signs of instability were detected only after 1 h of incubation. It should be noted that the main evidence of instability was given by the appearance of a new peak in the RP-HPLC chromatograms at *t*_R_ = 1.8 min, likely corresponding to free Lu-177. After 24 h, the differences between the compounds were more pronounced. While [^177^Lu]Lu-PepH3-1 (81.5% intact compound after 24 h) and [^177^Lu]Lu-PepH3-2 (95.3% intact compound after 24 h) showed a favorable stability profile and were highly improved with respect to the natural L-PepH3 sequence,^52^ the four TPP-containing peptides [^177^Lu]Lu-TPP-1, [^177^Lu]Lu-TPP-2, [^177^Lu]Lu-Comb-1, and [^177^Lu]Lu-Comb-2 were almost completely dissociated and featured half-lives between 90 min – 2 h (Fig. 3D).

To investigate whether the ability of the TPP sequence to target mHsp70 is preserved in these new constructs, the binding of the FITC-labeled compounds (FITC-TPP-2 and FITC-Comb-2) to mHsp70-expressing human glioblastoma U87-MG cells was tested (Fig. 3E–F and Table S4).^37^ The flow cytometric analysis of cells incubated with FITC-TPP-2 and FITC-Comb-2 showed binding of both compounds to U87-MG cells. Interestingly, FITC-TPP-2 showed a lower percentage of positively stained cells (46.2% ± 2.5), whereas more than 75% of cells showed binding of FITC-Comb-2 (77.5% ± 4.4). Since we hypothesize that the PepH3-2 moiety enables the conjugates to cross the cell membrane and reach the intracellular space,^50^ FITC-Comb-2 can feature increased binding compared to FITC-TPP-2, whose binding is limited to cell surface HSP70. The TPP-2 binding intensity matched the HSP70 expression level, as confirmed by staining the cells with the HSP70-targeting antibody FITC-cmHsp70.1. FITC-cmHsp70.1 showed much higher positivity (28.7% ± 6.0 compared to unstained cells) than the non-specific isotype control (11.2% ± 2.5 compared to unstained cells) (Fig. 3E).

It should be noted that FITC is pH-sensitive, and since our FITC-labeled probes are internalizing, this may impact the fluorescence readouts. However, FITC is brightest at neutral pH and is mainly quenched in acidic vesicles (lysosomes). We report the percentage of FITC-positive cells using a fixed gating strategy and consider any pH-dependent modulation of the fluorescence intensity to be too small to affect our qualitative classification of cells as positive or negative in this study. If anything, pH-based quenching would lead to an underestimation of positive cells, further elevating FITC-Comb-2 as the most promising ligand. In any case, previous studies have not shown fluorescence quenching with TPP-FITC conjugates.^37,60^

### *In vitro* evaluation of blood–brain barrier translocation

**Fig. 4:**
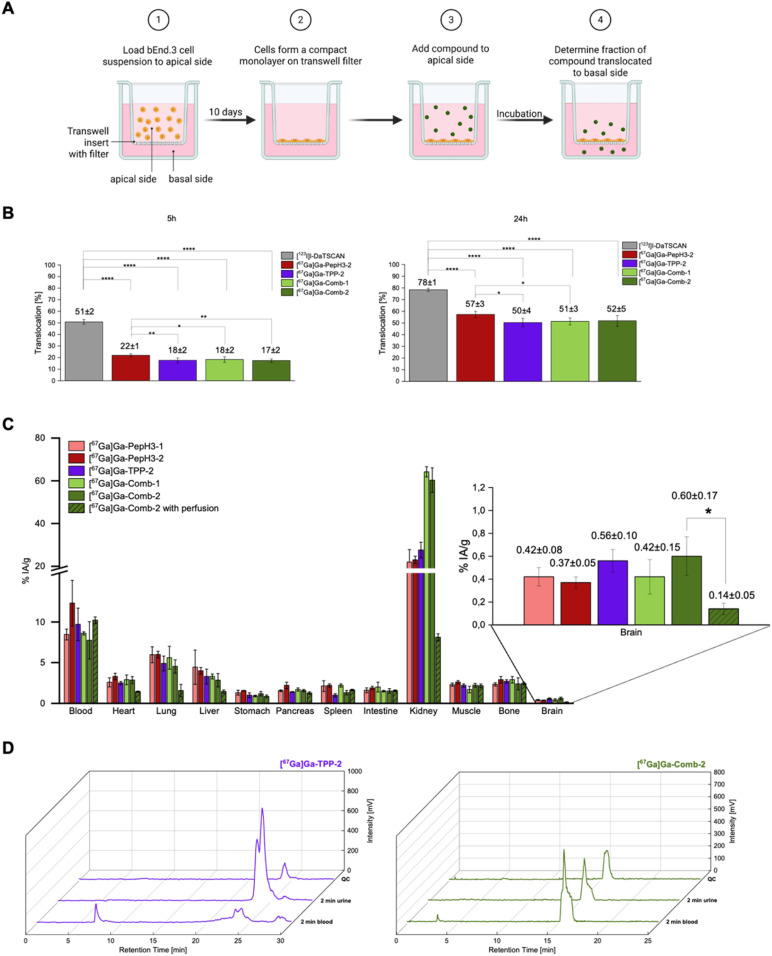
(A) Scheme of the BBB-translocation assay (created in BioRender). (B) BBB-translocation of the reported [^67^Ga]Ga radiotracers (185 kBq mL^−1^) after 5 h of incubation *in vitro*. Statistical analysis: one-way Anova with Tukey’s test, *: *p* < 0.05, **: *p* < 0.01, ***: *p* < 0.001, and ****: *p* < 0.0001. (C) *Ex vivo* biodistribution of the investigated [^67^Ga]Ga-labelled compounds (2.7–4.1 MBq) in naive CD1 mice, 2 min p.i. Data are expressed as % IA g^−1^, mean ± SD (*n* = 3). The exact values calculated for this diagram are given in the SI (Tables S6 and S7). (D) Selection of radio-RP-HPLC chromatograms from stability studies on blood and urine samples from mice sacrificed for biodistribution studies at 2 min p.i.

Aimed at assessing the ability of the novel conjugates to translocate the BBB, we used an advanced *in vitro* model established with murine brain endothelial cells (b.End3). The latter form functional barriers and express tight junction proteins in culture, thus mimicking BBB characteristics in this type of model.^61–63^ As described previously by us, this model was established by growing bEnd.3 cells in fibronectin-coated transwell filters, enabling the growth of a tight monolayer of cells (Fig. 4A).^61^ After formation of the cell monolayer, the radiopeptide derivatives [^67^Ga]Ga-PepH3-2, [^67^Ga]Ga-TPP-2, [^67^Ga]Ga-Comb-1, and [^67^Ga]Ga-Comb-2 were added to the apical side for 5 h. Additionally, [^123^I]I-ioflupane ([^123^I]I-DaTSCAN™) was used as a positive control for BBB translocation. Ioflupane is an approved radiopharmaceutical that can bind to dopamine transporters in the brain and has been indicated for the diagnosis of Parkinsonian syndromes in clinical settings since 2000.^64^ The translocation efficiency was determined by the amount of the radiolabeled compound detected in the basolateral medium as a % of the total content recovered (apical + basolateral media) (Fig. 4B). To assess whether the integrity of the BBB model was affected by the radiotracers, we performed a post-translocation integrity assay. To this end, the permeability of the BBB model to fluorescently labeled dextran with a 4 kDa molecular weight (FD4) was measured. No decrease in the integrity of the cell model (integrity >85%) was observed, indicating that the compounds did not disrupt the monolayer in the BBB *in vitro* model.

All radiotracers tested efficiently crossed the *in vitro* BBB model (Fig. 4B), in line with previous studies on peptide-based radiotracers.^50^ As expected, [^123^I]I-ioflupane presented the highest translocation level, which is significantly higher than those of the peptide conjugates (*p* < 0.0001 for all values). The translocation ability of [^67^Ga]Ga-PepH3-2, [^67^Ga]Ga-Comb-1, [^67^Ga]Ga-Comb-2, and [^67^Ga]Ga-TPP-2 was comparable, while a small but statistically significant difference was observed for [^67^Ga]Ga-PepH3-2 compared to the other compounds. This means that conjugation of the mHsp70-targeting peptide TPP to the PepH3 sequence had a negligible impact on the BBB-translocating properties of the chimeric radioconjugates.

Concerning the possible role of the charged [^67^Ga]-DOTA unit in facilitating PepH3 translocation, previously reported studies with a radiopeptide with a neutral metal moiety, [^67^Ga]-NODAGA-PepH3, showed similar translocation ability to the [^67^Ga]Ga-DOTA-D-PepH3-2 radiopeptide (27% and 22%, respectively) after 5 h of incubation.^50^ Therefore, we can exclude a substantial impact of the positively charged metal chelating group on the BBB translocation properties of the tracers.

To follow up on these results and to explore the comparable BBB translocation of the [^67^Ga]Ga-TPP-2 construct to the other PepH3-based compounds, we evaluated mHSP70 expression in bEnd.3 cells by flow cytometry and found that these cells also express mHSP70, although to a lower extent than U87 cells (Table S5 and Fig. S52). We hypothesize that this target expression in the endothelial cell model could lead to radiotracer binding and internalization, eventually allowing its translocation. It should be noted that it is extremely difficult to discern the BBB translocation capabilities of peptides that rely on AMT mechanisms (*e.g.*, PepH3) *vs.* those relying on receptor-mediated pathways (as is possible for TPP *via* mHSP70 binding). The mechanisms by which peptides interact with the endothelial cell membrane and translocate the BBB are diverse, with subtle differences, particularly for active mechanisms, and they often overlap in standard assays.

### *In vivo* studies

#### *Ex vivo* biodistribution studies

To further analyze the BBB penetration ability and the pharmacokinetic properties of the ^67^Ga-labeled compounds, biodistribution experiments were performed in naive female CD1 mice. In addition to the tracers tested in the BBB assay, [^67^Ga]Ga-PepH3-1 was included as well, to further analyze the effect of the linker length (glutamate *vs.* PEG_6_ linker). The mice were injected intravenously with the respective radiopeptide and sacrificed 2 min post-injection (p.i.) to assess the initial brain uptake at the first blood passage, and after 60 min p.i. The results are presented in Tables S6 and S7 and displayed in Fig. 4C. As expected, at 2 min p.i., the tissue distribution was at an early stage, resulting in high accumulation in blood and major organs. The pronounced activity accumulation in the kidneys (>20% IA g^−1^, injected activity per gram) suggests renal excretion as the predominant excretion pathway for all tested compounds.

Concerning brain uptake, the mean values at 2 min p.i. all ranged between 0.37 ± 0.05% IA g^−1^ for [^67^Ga]Ga-PepH3-2 and 0.60 ± 0.17% IA g^−1^ for [^67^Ga]Ga-Comb-2, with no statistically significant differences between them. In line with the observed translocation in the *in vitro* BBB model, [^67^Ga]Ga-TPP-2 (0.56 ± 0.1% IA g^−1^) showed brain accumulation comparable to that of the PepH3-containing compounds. In general, our results are in line with those previously reported by Neves *et al.*^50^ Moreover, brain uptake values in healthy or subcutaneous tumor-bearing mice of other reported peptide-based radiotracers designed to cross the BBB were between 0.3 ± 0.0 and 0.9 ± 1.1% IA g^−1^.^65^ In addition, the small molecule [^18^F]FET, which is routinely used for PET imaging of cancer, including in patients affected by GBM,^66,67^ showed brain uptake below 1% (0.99 ± 0.24% IA g^−1^, 5 min p.i.) in a mouse model bearing subcutaneous S180 tumors.^68^ This comparison suggests that our values (0.37 ± 0.05 to 0.60 ± 0.17% IA g^−1^) are also compatible with BBB crossing.

At 60 min p.i. (Table S7), radioactivity levels confirmed clearance of the tracers from the brain and major retention in the kidneys of [^67^Ga]Ga-Comb-1 and [^67^Ga]Ga-Comb-2 (91.88 ± 20.63 and 110.90 ± 16.80% IA g^−1^, respectively), which should be considered for future therapeutic applications. High kidney retention is a common feature of several radiolabeled peptides, often related to tubular reabsorption. Although this is a dose-limiting issue in clinical applications, there are well-established strategies to reduce renal reabsorption.^69^ Anyway, similar uptake has been observed with the commonly used PSMA ligands,^70^ which does not necessarily exclude our tracers from possible clinical imaging use.

For the best-performing compound in terms of brain uptake, [^67^Ga]Ga-Comb-2, the biodistribution experiment (2 min p.i.) was also performed with perfusion, to exclude the fraction of the radiotracer associated with the blood vessels in the brain. In the perfusion protocol, after sacrifice, the blood volume of the animal was exchanged with PBS before the removal of the organs. Therefore, the activity can be measured exclusively within the organ tissue itself. This lengthy procedure results in reduced activity values, particularly in highly vascularized organs. Accordingly, brain uptake was reduced from 0.60 ± 0.17 % IA g^−1^ before perfusion to 0.14 ± 0.05% IA g^−1^ at 2 min p.i. after perfusion (Fig. 4C). The statistical analysis of this value showed that it was significantly different from the brain uptake of [^67^Ga]Ga-Comb-2 without perfusion (*p* < 0.05). Nevertheless, the activity left in the brain is still relevant, especially when compared with that of the peptide-free *fac*-[^99m^Tc(CO)_3_]-pyrazol-diamine chelator ([^99m^Tc]TcPz3), which served as a negative control in the studies performed by Neves *et al.*^50^ (brain uptake of 0.09 ± 0.01% IA g^−1^, 5 min p.i.). Therefore, the obtained results suggested that [^67^Ga]Ga-Comb-2 was able to cross the BBB.

#### *Ex vivo* stability studies

To further investigate the stability of the compounds *in vivo*, radio-RP-HPLC measurements were performed on blood and urine samples from mice sacrificed for biodistribution studies. While urine samples from three mice were collected, combined, and filtered before injection, the blood sample with the highest radioactivity was used after protein precipitation and filtration. The results are displayed in Fig. 4D and S53. The PepH3-containing compounds [^67^Ga]Ga-PepH3-1, [^67^Ga]Ga-PepH3-2, [^67^Ga]Ga-Comb-1, and [^67^Ga]Ga-Comb-2 showed high stability in both blood and urine at 2 min p.i. [^67^Ga]Ga-Comb-2 revealed only a small additional signal at a retention time of *t*_R_ = 3.0 min in blood, which is most likely attributable to free [^67^Ga]Ga^3+^. The intact [^67^Ga]Ga-PepH3-1 exhibited a very defined signal at *t*_R_ = 11.8 min in the radio-RP-HPLC quality control.

Of note, [^67^Ga]Ga-TPP-2 formed several metabolites (Fig. 4D), revealing *in vivo* stability issues, as already noted in the *in vitro* human serum study. In urine, the retention time shifted to *t*_R_ = 10.3 min, and thus, to a more hydrophilic region compared to the signal of the intact compound at *t*_R_ = 12.6 min. In blood, the signal of the intact compound was observed, together with several signals of shorter retention time, suggesting the formation of a more hydrophilic species. While it is still reasonable to assume that the uptake in the brain was partly due to the intact [^67^Ga]Ga-TPP-2, the value could also be influenced by the formed metabolites. Overall, the *in vivo* results indicate that the combination with the d-PepH3 sequence in [^67^Ga]Ga-Comb-1 and [^67^Ga]Ga-Comb-2 significantly stabilized the l-TPP sequence.

## Conclusions

In summary, we have synthesized two chimeric radiotracers that can be radiolabeled with nuclides suitable for imaging and therapy. The performed *in vitro* and *in vivo* experiments demonstrate that the compounds can both target brain cancer cells and translocate the BBB. Our results provide a sufficient basis for further investigation and optimization of the combinatorial peptides. For example, ongoing work involves conjugating the PepH3 peptide to CXCR4-targeting peptidic vectors for brain cancer imaging and treatment. In the future, dynamic PET/MRI or PET/CT imaging would help further assess brain uptake of the compounds and identify the optimal time point for maximal brain uptake in future biodistribution experiments. These efforts could lead to the development of radiopharmaceutical compounds for GBMtheranostics.

## Experimental section

### General

All reagents and solvents were purchased from commercial suppliers and used without further purification. ESI-MS spectra were recorded on an expression^L^ CMS mass spectrometer (Advion Ltd, Harlow, UK) with a quadrupole analyzer and an electrospray ionizer. To obtain high-resolution ESI-MS (HR-ESI-MS) of the end products, an Exactive Plus Orbitrap Mass Spectrometer from Thermo Fisher Scientific (Massachusetts, USA) was used. Simulated spectra were calculated with the free enviPat webpage, provided by EAWAG (Dübendorf, Switzerland).

Analytical and preparative RP-HPLC was carried out on Shimadzu Corp. Instruments (Kyoto, Japan) equipped with two LC-20AD gradient pumps, a CBM-20A communications module and a Smartline UV detector 2500 (*λ* = 220 nm, *λ* = 254 nm) from Dr Ing. Herbert Knauer GmbH (Berlin, Germany). For analytical RP-HPLC a flow rate of 1.0 mL min^−1^ and for preparative RP-HPLC a flow rate of 10 mL min^−1^ were used. Quality controls of peptidic ligands were performed on a MultoKrom^®^ 100-5-C8 column (150 × 4.6 mm, 5 µm particle size, CS Chromatographie GmbH, Langerwehe, Germany). Different gradients of A (H_2_O + 0.1% TFA) and B (MeCN + 5% H_2_O and 0.1% TFA) were used as eluents for all RP-HPLC operations, except for the experiment with ^67^Ga-labeled compounds, where A (H_2_O + 0.1% TFA) and B (Methanol) were used as eluents on a Nucleosil 100–5 C18 column (250 mm × 4.6 mm, 5 µm) from Macherey-Nagel (Düren, Germany). All compounds are >95% pure by HPLC analysis.

An Alpha 1.2 freeze-dryer from Christ (Osterode, Germany) connected to a RZ-2 rotary vane pump from Vacuubrand GmbH & Co. KG (Wertheim, Germany) was used to freeze-dry products. Human glioblastoma U87-MG cells were purchased from ATCC (HTB-14) and cultivated in DMEM GlutaMAX™ from Thermo Fisher Scientific (Waltham, United States) with 10% FBS purchased from FBS Zellkultur (Berlin, Germany) or Thermo Fisher Scientific (Waltham, United States) and 1% penicillin (10 000 units)/streptomycin (10 mg mL^−1^) in 0.9% NaCl, purchased from Sigma-Aldrich Co. (St. Louis, United States) as supplements. The murine brain endothelial cell line b.End3 was purchased from ATCC (Virginia, USA) and cultivated in DMEM from Thermo Fisher Scientific (Waltham, United States) with 10% FBS purchased from Thermo Fisher Scientific (Waltham, United States). The cells were cultivated at 37 °C in a humidified 5% CO_2_ atmosphere in a cell cabinet. They were handled under sterile conditions in laminar workflow stations.

[^177^Lu]LuCl_3_ in 0.04 M HCl was purchased in radiochemical grade EndolucinBeta® n.*c.a.* (40 GBq mL^−1^) from ITM Isotope Technologies Munich SE (Munich, Germany) and was used without further purification. [^67^Ga]Gallium citrate in water was purchased from Curium Netherlands B.V. (Le Petten, Netherlands) and was transferred to [^67^Ga]GaCl_3_ in 0.1 M HCl before further use. All radioactive samples were measured on a 2480 Wizard γ-counter from PerkinElmer Inc. (Waltham, USA), or a Hidex Automatic γ-counter from Hidex Oy (Turku, Finland).

### Peptide synthesis and purification

The compounds were acquired using manual solid-phase peptide-synthesis (SPPS) on the 2-CTC resin or a combination of manual and automated SPPS (using LibertyBlue™ from CEM Corporation) on the ProTide Rink amide or the Fmoc-Gly-Wang-ProTide resin. Manual coupling steps were performed in DMF in a SPPS reactor equipped with a polyethylene frit for at least 2 h at rt. Here, the coupling reagents TBTU (1.5 eq.) and HOAt (1.5 eq.), as well as the base DIEA (6.0 eq. or more to ensure a pH of 9–10) were added. The resin was swollen for at least 1 h at rt in DMF before the reaction, and 1.5 eq. of the respective Fmoc-protected amino acid were used for the coupling. Fmoc deprotection between the coupling steps was realized with 20% piperidine/DMF for 5 + 10 + 15 min at rt. When coupling DOTA(*t*Bu)_3_, 3.0 eq. of the chelator and the coupling reagents and 9.0 eq. of DIEA were applied. Coupling of FITC-isomer 1 (2.5 eq.) was performed with 8.0 eq. DIEA and no coupling reagents.

During automated SPPS, DMF was used as a solvent, DIC (0.5 M, 10 eq.) in DMF as the activator and Oxyma/DIEA (0.5 M/0.1 M, 5 eq./2 eq.) as the activator base. The Fmoc-protected amino acids (0.2 M, 10 eq.) were coupled at 90 °C for 2 min. For Fmoc-Arg(Pbf)-OH, the coupling was performed two times in a row. Fmoc deprotection was achieved using 20% piperidine/DMF for 1 min at 90 °C.

Cleavage from the resin after the completion of the peptide was achieved by adding a TFA/TIPS/H_2_O (95/2.5/2.5) mixture for 1 h + 1 h at rt. To ensure the deprotection of all amino acid residues, the cocktail was stirred for at least 2 h at rt. Then, TFA was evaporated under a nitrogen current, and the crude peptide was dissolved in a mixture of H_2_O, MeCN, and DMF, to achieve a clear solution, filtered, and purified by RP-HPLC. After evaporating the HPLC solvents under reduced pressure and lyophilization, the product was obtained in high purity (≥95%).

### Radiolabeling

For the complexation with lutetium-177, each compound (1 µL, 1 mM in DMSO) was added to 10 µL of sodium acetate buffer (1 M, pH 5.5) in a Protein LoBind Eppendorf tube (Merck KGaA (Darmstadt, Germany): EP0030108116). Depending on the planned experiments, a varying amount of [^177^Lu]LuCl_3_ in 0.04 M HCl was added (about 20–55 MBq nmol^−1^). After the volume was adjusted to 80 µL, the reaction mixture was heated to 80–90 °C for 15–20 min. Subsequently, 20 µL of the radiolysis quencher sodium ascorbate (0.1 M in PBS) were added, and the final volume was adjusted to 100 µL with 0.04 M HCl. Quality control was performed *via* radio RP-HPLC (10–40% or 10–50% MeCN (2% H_2_O and 0.1% TFA)/H_2_O (0.1% TFA) v/v in 15 min) on a MultoKrom® 100 RP 18 column (125 × 4.6 mm, 5 µM particle size) purchased from CS – Chromatographie Service GmbH (Langerwehe, Germany).

For the complexation with gallium-67 the received [^67^Ga]gallium-citrate (CURIUM NETHERLANDS B.V., the Netherlands) was first transformed to [^67^Ga]GaCl_3_ by ionic exchange using a Sep-Pak® Classic Silica column (690 mg, 50–105 µm, Waters™, Milford, USA) and eluting with 3 mL 0.1 M HCl in 0.5 mL fractions.^71^ The respective amount of 0.1 M NaOAc buffer (pH = 5) was added to the needed activity of [^67^Ga]GaCl_3_ (about 0.1–2.2 MBq nmol^−1^), to reach a pH of 4–5. The respective amount of DOTA-bearing compound was added to achieve a concentration of 50 µm. The reaction mixture was heated for 15 min (80–95 °C), and quality control *via* radio RP-HPLC (10% MeOH/H_2_O (0.1% TFA) v/v for 5 min and 10–100% MeCN/H_2_O (0.1% TFA) v/v in 20 min) was performed on a NUCLEOSIL 100–5 C18 column (250 mm × 4.6 mm, 5 µm) from MACHEREY-NAGEL (Düren, Germany).

### *In silico* predictive BBB-translocation

To calculate the theoretical capabilities of the peptides to cross the BBB, we have applied a previously described predictive model.^53^ Initially, we calculated the physicochemical properties of each peptide and the sum of squares (*S*), with lower *S* indicating greater capacity to cross the BBB. Then, for a better comparison with the current BBB-penetrating peptides, we calculated the relative translocation capacity using positive and negative controls (with a translocation factor (cross) of 1 or 0, respectively). The crossing capacity is reported as high (cross >0.80); moderate (0.50 > cross > 0.80); or low (cross <0.50).

### Lipophilicity (log *D*_7.4_)

The octanol-PBS partition coefficient at pH = 7.4 (log *D*_7.4_) was determined as follows. A mixture of 500 µL PBS (pH 7.4) buffer and 500 µL octanol was transferred to a Protein LoBind Eppendorf reaction tube (Merck KGaA (Darmstadt, Germany): EP0030108116). Furthermore, 0.5–1 MBq of the [^177^Lu]Lu labelled compound were added (the added volume did not exceed 10 µL). The samples were vortexed for 3 min at 3000 rpm and centrifuged for 5 min at 9000 rpm. Afterwards, 200 µL of the octanol phase and 200 µL of the PBS phase were carefully withdrawn. For very hydrophilic compounds, with log *D*_7.4_ < −3, the measurement was repeated with only 20 µL of PBS withdrawn, to avoid exceeding the linear measurement window of the γ-counter. The activity of the separated octanol and PBS phases was measured using a γ-counter (PerkinElmer Inc. Langerwehe, Germany). For each compound, the experiment was repeated with a total of eight samples. The log *D*_7.4_ was determined for each sample *via* the following equation. The mean value was obtained using at least 5 of the 8 measured samples.
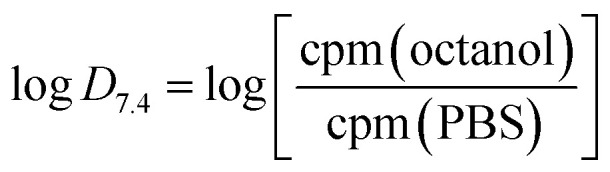


### Human serum albumin (HSA) binding

After radioactive labeling with [^177^Lu]Lu and quality control *via* radio RP-HPLC, the compounds were diluted in PBS (pH 7.4) buffer to obtain stock solutions with an activity concentration of 20 MBq mL^−1^. Additionally, a stock HSA solution was prepared by dissolving HSA in PBS buffer to reach a concentration of 777.8 µM. Thus, 6 samples containing the [^177^Lu]Lu labelled compound at a concentration of 2 MBq mL^−1^ were prepared for each peptidic derivative, by adding 25 µL of the respective stock solutions to 225 µL of the HSA/PBS solution in Protein LoBind Eppendorf tubes (Merck KGaA (Darmstadt, Germany): EP0030108116), in order to obtain a compound:HSA ratio of about 1 : 10 000. Thereby, a final HSA concentration of 700 µm was achieved, which is equivalent to the physiological HSA concentration in the blood (30–50 g L^−1^, 452–753 µM).^72^ In parallel, 6 control dilutions of each compound were prepared by adding 25 µL of the stock solutions to 225 µL PBS. All samples were then incubated for 30 min in the cell incubator (37 °C and 5% CO_2_). Thereafter, each sample was transferred to a Centrifree® centrifugal filter unit (Merck Millipore Ltd, Cork, Ireland) and centrifuged at 3200 rpm for 40 min. The filter and the filtrate were separated and measured separately in the γ-counter. The HSA-binding was calculated using the cpm-values and the following equations. Thereby, the total HSA binding was corrected for the non-selective binding to the ultrafiltration vial using the controls.
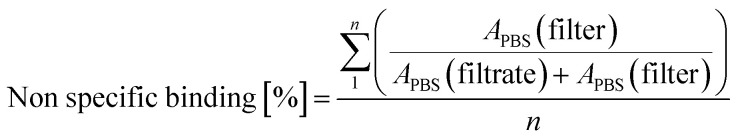

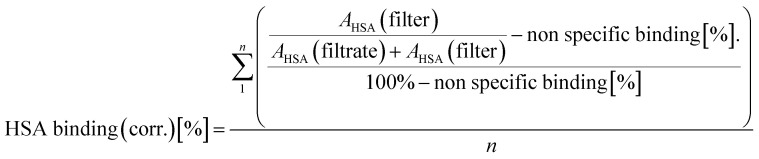


### Stability studies

For the stability studies, the [^177^Lu]Lu-labeled peptides were transferred to Protein LoBind Eppendorf tubes (Merck KGaA (Darmstadt, Germany): EP0030108116), diluted with the respective medium and incubated in a cell safety cabinet (37 °C and 5% CO_2_) for the respective amount of time. For the stability in DMEM GlutaMAX™, one vial for the 4 time points was prepared (28 µL (12 MBq) compound + 372 µL DMEM, 1 : 13 v/v). Samples of 100 µL were taken at each time point, and 75–80 µL were immediately injected to perform the RP-HPLC measurement. For stability in human serum (H4522 from Sigma-Aldrich, US), 1 vial was prepared for each of the 3 time points (30 µL (6–9 MBq) compound + 300 µL DMEM, 1 : 10 v/v). Samples of 100 µL were taken at each time point and transferred to a new Protein LoBind Eppendorf tube, where 125 µL cold MeCN and 50 µL cold EtOH were added to precipitate serum proteins. The tubes were centrifuged for 5 min at 5000 rpm; the supernatant was transferred to centrifugal filters (VWR International GmbH (Darmstadt, Germany): 516–0231) and centrifuged again for 5 min at 5000 rpm. Then, 50 µL of the filtrate were injected to perform RP-HPLC measurement. The gradient used for all measurements was 10–50% B over 15 min. Integration of the resulting HERM signals was used to determine the percentage of intact compounds.

### Flow cytometry

Preparations for the cytometry experiments began by trypsinizing the U87-MG or bEnd.3 cells and washing them with PBS containing 1% FBS. Incubation was performed with 5 µM of the compounds in PBS (0.1% FBS for peptides and 1% FBS for the Mouse Anti-HSPA1A Recombinant Antibody (clone cmHsp70.1, Creative Biolabs, Cat No: HPAB-0243-YC) and the AF488 isotype control (B434947, Biolegend)) at 37 °C for 30 or 60 min. After subsequent washing, the cells were resuspended in PBS with 1% FBS and stained with DAPI. Measurements were performed using a NovoCyte 2060R and processed with the NovoExpress 1.6.0 software from Agilent Technologies Inc. (Santa Clara, California, USA). The flow cytometry gating strategy was performed according to a standardized sequential workflow. Briefly, the cell population was selected by gating on forward and side scatter, followed by doublet discrimination to isolate single-cell events. Viable cells were identified by exclusion of DAPI-positive events (dead cells).

### Blood–brain barrier model

#### Cell culture

Murine brain endothelial cells, bEnd.3 (American Type Culture Collection, CRL-2299TM) were cultured in Dulbecco's Modified Eagle Medium (DMEM) supplemented with 10% fetal bovine serum (FBS) (all from Gibco, Thermo Fisher Scientific) in a humidified atmosphere of 5% CO_2_ at 37 °C. The cells were tested for mycoplasma using the LookOut® mycoplasma Polymerase Chain Reaction (PCR) Detection kit (Sigma-Aldrich, Merck). To obtain the *in vitro* BBB model, 200 µL of a cell suspension with 5000 cells were seeded on the apical side of fibronectin-coated tissue culture Polyethylene Terephthalate (PET) inserts (pore size of 1 µm) for 24-well plates (Corning, Merck), and 500 µL of complete culture medium were added to the basal side of the inserts. The cells were grown for 10 days, and the media of both the apical and basal sides were changed every other day. On the day of the translocation assay, the integrity of every filter was checked after the translocation assay was performed.

#### BBB translocation assay

For the translocation assay, the filters were washed two times with PBS and one time with assay medium (DMEM FluoroBrite™ (+10% FBS)). Afterwards, 500 µL of assay medium was added to the basolateral side, while 200 µL of solution containing 185 kBq mL^−1^ of the ^67^Ga-compound under investigation, diluted in assay medium, was added to the apical side. Incubation was performed for 5 h at 37 °C in a humidified 5% CO_2_ atmosphere. Afterwards, contents of each apical and basolateral side were transferred to a centrifuge tube and measured in a γ-counter.

#### BBB integrity assay

To evaluate the integrity of the BBB *in vitro* model, the fluorescent probe fluorescein isothiocyanate-dextran with a molecular weight of 4 kDa (FD4, Sigma-Aldrich) was used. During the incubation of the radioactive probe, 2 h before the end of that incubation, 2 µL FD4 were added to the apical side to obtain a final concentration of 25 µg mL^−1^ in DMEM FluoroBrite^™^. After the measurement in the γ-counter, the fluorescence was measured (*ex.* 493 nm em^−1^ 560 nm) in a microplate reader (Varioskan Lux multimode, Thermo Fisher Scientific). Empty inserts were used as the control. The integrity of the cell layer was determined by the following calculation and was required to be at least 90% for the translocation values to be considered valid.
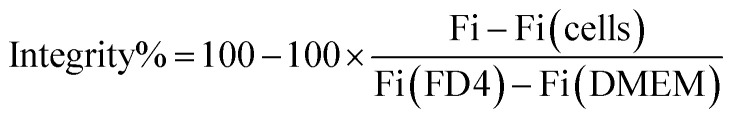
Fi: fluorescence intensity of the basal side of the controlled filter; Fi(cells): fluorescence intensity of the basal side of the filter only incubated with medium; Fi(FD4): fluorescence intensity of total FD4 initially added to each filter transwell; Fi(DMEM): fluorescence intensity of medium only.

#### Biodistribution and *in vivo* stability studies

Animal experiments were performed by certified personnel only, accredited by the National Authorities (Direção-Geral da Alimentação e Veterinária – DGAV) and with the research project approved by the local ethical committee and the respective National Authority. They were performed in agreement with National and European Union directives regarding ethics, care, and protection of animals used for experimental and other scientific purposes. The animal housing was also approved by the DGAV and consisted of a temperature- and humidity-controlled room with a 12-hour light/dark schedule.

For biodistribution experiments, naive, 8-week-old female CD1 mice were injected intravenously in the tail vein with 100 µL of a 0.9% (m/v) NaCl solution containing 2.7–4.1 MBq of the [^67^Ga]Ga-labeled compounds. The mice were sacrificed by cervical dislocation at 2 min p.i. and weighed; subsequently, urine and blood were collected, and the main organs were removed, rinsed, weighed and measured for radioactivity in a Hidex AMG Automatic γ-counter. The resulting organ activities were expressed as a percentage of injected activity per gram of tissue (% IA g^−1^). In the experiment with perfusion, the blood volume of the animal was exchanged with PBS before the organ removal.

For *in vivo* stability studies, the urine samples of three mice treated with the same compound were combined after γ-counter measurement. The combined samples were centrifuged at 2000*g* for 10 min prior to being analyzed by RP-HPLC. For the blood samples, the one with the highest remaining activity was chosen. The sample was prepared by centrifugation at 2000*g* for 10 min to separate the blood serum, followed by precipitation of the serum proteins using cold EtOH in a 2 : 1 (v/v) ratio (ethanol/serum) and centrifugation at 2000*g* for 5 min. Radio RP-HPLC analysis was used for the evaluation of the *in vivo* stability, using the same experimental procedure reported above for the radiochemical purity assessment of the [^67^Ga]Ga-containing compounds.

## Author contributions

A. C. and F. S. conceived the research, designed experiments, performed data analysis, and drafted the manuscript; F. M., J. G. C., and S. K. designed experiments and performed data analysis; F. S. synthesized and characterized the compounds. F. S. and C. I. G. P. performed the BBB translocation assays. R. D. M. S. and L. G. performed the *in vivo* studies. L. K., F. S., and S. S. performed cytometry analysis. M. C. and M. A. R. C. performed the *in silico* BBB translocation studies and contributed to the translocation result analysis. A. C., J. G. C., F. M., S. C., and M. A. R. C. secured funding and provided essential resources. All authors contributed to manuscript writing. All authors have given approval to the final version of the manuscript.

## Conflicts of interest

The authors declare no competing financial interest.

## Supplementary Material

SC-017-D6SC00011H-s001

## Data Availability

Original cytometry data files have been provided on Zenodo, DOI: https://doi.org/10.5281/zenodo.20183561. The data supporting this article, including abbreviations, additional experimental procedures, characterization data, additional figures and tables, chromatograms, and spectra, are included in the supplementary information (SI). Supplementary information: details on the synthesis and characterization of peptide-based radiotracers, as well as RP-HPLC chromatograms and (HR-) ESI-MS spectra of quality controls and radioactive labeling; and data on stability studies, cytometry analysis, *in silico* prediction of BBB translocation, biodistribution, and metabolic studies. See DOI: https://doi.org/10.1039/d6sc00011h.

## References

[cit1] Sharma A., Guerrero-Cazares H., Maciaczyk J. (2023). Editorial to Special Issue “Glioblastoma: Recapitulating the Key Breakthroughs and Future Perspective”. Int. J. Mol. Sci..

[cit2] Zheng X., Tang Q., Ren L., Liu J., Li W., Fu W., Wang J., Du G. (2021). A narrative review of research progress on drug therapies for glioblastoma multiforme. Ann. Transl. Med..

[cit3] Shergalis A., Bankhead 3rd A., Luesakul U., Muangsin N., Neamati N. (2018). Current Challenges and Opportunities in Treating Glioblastoma. Pharmacol. Rev..

[cit4] Lerouge L., Ruch A., Pierson J., Thomas N., Barberi-Heyob M. (2024). Non-targeted effects of radiation therapy for glioblastoma. Heliyon.

[cit5] Weller M., Albert N. L., Galldiks N., Bink A., Preusser M., Sulman E. P., Treyer V., Wen P. Y., Tonn J. C., Le Rhun E. (2024). Targeted radionuclide therapy for gliomas: Emerging clinical trial landscape. Neuro Oncol..

[cit6] Waked A., Crabbe M., Neirinckx V., Perez S. R., Wellens J., Rogister B., Benotmane M. A., Vermeulen K. (2024). Preclinical evaluation of CXCR4 peptides for targeted radionuclide therapy in glioblastoma. EJNMMI Radiopharm. Chem..

[cit7] Lapa C., Luckerath K., Kleinlein I., Monoranu C. M., Linsenmann T., Kessler A. F., Rudelius M., Kropf S., Buck A. K., Ernestus R. I., Wester H. J., Lohr M., Herrmann K. (2016). (68)Ga-Pentixafor-PET/CT for Imaging of Chemokine Receptor 4 Expression in Glioblastoma. Theranostics.

[cit8] Wang S., Wang J., Liu D., Yang D. (2018). The value of 68Ga-PSMA-617 PET_CT in differential diagnosis between low-grade and high-grade gliomas. J. Nucl. Med..

[cit9] Marafi F., Sasikumar A., Fathallah W., Esmail A. (2020). 18F-PSMA 1007 Brain PET/CT Imaging in Glioma Recurrence. Clin. Nucl. Med..

[cit10] Li L., Quang S. T., Gracely E. J., Kim J. H., Emrich J. G., Yaeger T. E., Jenrette J. M., Cohen S. C., Black P., Brady L. W. (2010). A Phase II study of anti-epidermal growth factor receptor radioimmunotherapy in the treatment of glioblastoma multiforme. J. Neurosurg..

[cit11] Bigner D. D., Brown M. T., Friedman A. H., Coleman R. E., Akabani G., Friedman H. S., Thorstad W. L., McLendon R. E., Bigner S. H., Zhao X. G., Pegram C. N., Wikstrand C. J., Herndon 2nd J. E., Vick N. A., Paleologos N., Cokgor I., Provenzale J. M., Zalutsky M. R. (1998). Iodine-131-labeled antitenascin monoclonal antibody 81C6 treatment of patients with recurrent malignant gliomas: phase I trial results. J. Clin. Oncol..

[cit12] Reardon D. A., Quinn J. A., Akabani G., Coleman R. E., Friedman A. H., Friedman H. S., Herndon J. E., McLendon R. E., Pegram C. N., Provenzale J. M., Dowell J. M., Rich J. N., Vredenburgh J. J., Desjardins A., Sampson J. H., Gururangan S., Wong T. Z., Badruddoja M. A., Zhao X.-G., Bigne D. D., Zalutsky M. R. (2006). Novel Human IgG2b/Murine Chimeric Antitenascin Monoclonal Antibody Construct Radiolabeled with 131I and Administered into the Surgically Created Resection Cavity of Patients with Malignant Glioma: Phase I Trial Results. J. Nucl. Med..

[cit13] Heute D., Kostron H., von Guggenberg E., Ingorokva S., Gabriel M., Dobrozemsky G., Stockhammer G., Virgolini I. J. (2010). Response of recurrent high-grade glioma to treatment with (90)Y-DOTATOC. J. Nucl. Med..

[cit14] Schumacher T., Hofer S., Eichhorn K., Wasner M., Zimmerer S., Freitag P., Probst A., Gratzl O., Reubi J. C., Maecke R., Mueller-Brand J., Merlo A. (2002). Local injection of the 90Y-labelled peptidic vector DOTATOC to control gliomas of WHO grades II and III: an extended pilot study. Eur. J. Nucl. Med. Mol. Imag..

[cit15] Telix Pharmaceuticals (Innovations) Pty Limited , 131I-TLX-101 for Treatment of Newly Diagnosed Glioblastoma (IPAX-2) (IPAX-2) clinicaltrials.gov, 2022, https://clinicaltrials.gov/study/NCT05450744?term=NCT05450744&rank=1, 16.09.2025

[cit16] Pharmaceuticals N. , A Dose Finding Study of [177Lu]Lu-DOTA-TATE in Newly Diagnosed Glioblastoma in Combination With Standard of Care and in Recurrent Glioblastoma as a Single Agent.clinicaltrials.gov, 2021, https://clinicaltrials.gov/study/NCT05109728?term=NCT05109728&rank=1, 16.09.2025

[cit17] Advanced Accelerator Applications , [177Lu]-NeoB in Patients With Advanced Solid Tumors and With [68Ga]-NeoB Lesion Uptake (NeoRay), clinicaltrials.gov, 2019, https://clinicaltrials.gov/study/NCT03872778?term=NCT03872778&rank=1&tab=history, 16.09.2025

[cit18] University Hospital Muenster , Radioimmunotherapy with Lu-177 Labeled 6A10 Fab-fragments in Patients with Glioblastoma After Standard Treatment (RIT in GBM), clinicaltrials.gov, 2022, https://clinicaltrials.gov/study/NCT05533242?term=NCT05533242&rank=1&tab=history, 16.09.2025

[cit19] Iglesia R. P., Fernandes C. F. L., Coelho B. P., Prado M. B., Melo Escobar M. I., Almeida G., Lopes M. H. (2019). Heat Shock Proteins in Glioblastoma Biology: Where Do We Stand?. Int. J. Mol. Sci..

[cit20] Elmallah M. I. Y., Cordonnier M., Vautrot V., Chanteloup G., Garrido1 C., Gobbo J. (2020). Hsp70 in cancer: role of the membrane-anchored chaperone. Cancer Lett..

[cit21] Zhao K., Zhou G., Liu Y., Zhang J., Chen Y., Liu L., Zhang G. (2023). HSP70 Family in Cancer: Signaling Mechanisms and Therapeutic Advances. Biomolecules.

[cit22] Ferrarini M., Heltai S., Zocchi M. R., Rugarli C. (1992). Unusual expression and localization of heat-shock proteins in human tumor cells. Int. J. Cancer.

[cit23] Multhoff G., Botzler C., Wiesnet M., Muller E., Meier T., Wilmanns W., Issels R. D. (1995). A stress-inducible 72-kDa heat-shock protein (HSP72) is expressed on the surface of human tumor cells, but not on normal cells. Int. J. Cancer.

[cit24] Gehrmann M., Stangl S., Foulds G. A., Oellinger R., Breuninger S., Rad R., Pockley A. G., Multhoff G. (2014). Tumor imaging and targeting potential of an Hsp70-derived 14-mer peptide. PLoS One.

[cit25] Pfister K., Radons J., Busch R., Tidball J. G., Pfeifer M., Freitag L., Feldmann H. J., Milani V., Issels R., Multhoff G. (2007). Patient survival by Hsp70 membrane phenotype: association with different routes of metastasis. Cancer.

[cit26] Steiner K., Graf M., Hecht K., Reif S., Rossbacher L., Pfister K., Kolb H. J., Schmetzer H. M., Multhoff G. (2006). High HSP70-membrane expression on leukemic cells from patients with acute myeloid leukemia is associated with a worse prognosis. Leukemia.

[cit27] Farkas B., Hantschel M., Magyarlaki M., Becker B., Scherer K., Landthaler M., Pfister K., Gehrmann M., Gross C., Mackensen A., Multhoff G. (2003). Heat shock protein 70 membrane expression and melanoma-associated marker phenotype in primary and metastatic melanoma. Melanoma Res..

[cit28] Gehrmann M., Doss B. T., Wagner M., Zettlitz K. A., Kontermann R. E., Foulds G., Pockley A. G., Multhoff G. (2011). A novel expression and purification system for the production of enzymatic and biologically active human granzyme B. J. Immunol. Methods.

[cit29] Botzler C., Issels R., Multhoff G. (1996). Heat-shock protein 72 cell-surface expression on human lung carcinoma cells is associated with an increased sensitivity to lysis mediated by adherent natural killer cells. Cancer Immunol. Immunother..

[cit30] Botzler C., Schmidt J., Luz A., Jennen L., Issels R., Multhoff G. (1998). Differential Hsp70 Plasmamembrane Expression on Primary Human Tumors and Metastases in Mice with Severe Combined Immunodeficiency. Int. J. Cancer.

[cit31] Multhoff G. (2007). Heat shock protein 70 (Hsp70): membrane location, export and immunological relevance. Methods.

[cit32] Lobinger D., Gempt J., Sievert W., Barz M., Schmitt S., Nguyen H. T., Stangl S., Werner C., Wang F., Wu Z., Fan H., Zanth H., Shevtsov M., Pilz M., Riederer I., Schwab M., Schlegel J., Multhoff G. (2021). Potential Role of Hsp70 and Activated NK Cells for Prediction of Prognosis in Glioblastoma Patients. Front. Mol. Biosci..

[cit33] Multhoff G., Hafner M., Pfister K., Gehrmann M., Hiddemann W. (2001). A 14-mer Hsp70 peptide stimulates natural killer (NK) cell activity. Cell Stress Chaperones.

[cit34] Stangl S., Gehrmann M., Riegger J., Kuhs K., Riederer I., Sievert W., Hube K., Mocikat R., Dressel R., Kremmer E., Pockley A. G., Friedrich L., Vigh L., Skerra A., Multhoff G. (2011). Targeting membrane heat-shock protein 70 (Hsp70) on tumors by cmHsp70.1 antibody. Proc. Natl. Acad. Sci. U. S. A..

[cit35] Multhoff G., Mizzen L., Winchester C. C., Milner C. M., Wenk S., Eissner G., Kampinga H. H., Laumbacher B., Johnson J. (1999). Heat shock protein 70 (Hsp70) stimulates proliferation and cytolytic activity of natural killer cells. Exp. Hematol..

[cit36] Stangl S., Varga J., Freysoldt B., Trajkovic-Arsic M., Siveke J. T., Greten F. R., Ntziachristos V., Multhoff G. (2014). Selective *in vivo* imaging of syngeneic, spontaneous, and xenograft tumors using a novel tumor cell-specific hsp70 peptide-based probe. Cancer Res..

[cit37] Stangl S., Tei L., De Rose F., Reder S., Martinelli J., Sievert W., Shevtsov M., Ollinger R., Rad R., Schwaiger M., D'Alessandria C., Multhoff G. (2018). Preclinical Evaluation of the Hsp70 Peptide Tracer TPP-PEG(24)-DFO[(89)Zr] for Tumor-Specific PET/CT Imaging. Cancer Res..

[cit38] Oller-Salvia B., Sanchez-Navarro M., Giralt E., Teixido M. (2016). Blood–brain barrier shuttle peptides: an emerging paradigm for brain delivery. Chem. Soc. Rev..

[cit39] Wu D., Chen Q., Chen X., Han F., Chen Z., Wang Y. (2023). The blood–brain barrier: structure, regulation, and drug delivery. Signal Transduct. Targeted Ther..

[cit40] Arvanitis C. D., Ferraro G. B., Jain R. K. (2020). The blood–brain barrier and blood-tumour barrier in brain tumours and metastases. Nat. Rev. Cancer.

[cit41] Pawar B., Vasdev N., Gupta T., Mhatre M., More A., Anup N., Tekade R. K. (2022). Current Update on Transcellular Brain Drug Delivery. Pharmaceutics.

[cit42] Cavaco M., Gaspar D., Arb Castanho M., Neves V. (2020). Antibodies for the Treatment of Brain Metastases, a Dream or a Reality?. Pharmaceutics.

[cit43] Chacko A. M., Li C., Pryma D. A., Brem S., Coukos G., Muzykantov V. (2013). Targeted delivery of antibody-based therapeutic and imaging agents to CNS tumors: crossing the blood–brain barrier divide. Expert Opin. Drug Deliv..

[cit44] Kreuter J. (2014). Drug delivery to the central nervous system by polymeric nanoparticles: what do we know?. Adv. Drug Deliv. Rev..

[cit45] Bolcaen J., Kleynhans J., Nair S., Verhoeven J., Goethals I., Sathekge M., Vandevoorde C., Ebenhan T. (2021). A perspective on the radiopharmaceutical requirements for imaging and therapy of glioblastoma. Theranostics.

[cit46] Baghirov H. (2025). Mechanisms of receptor-mediated transcytosis at the blood–brain barrier. J. Contr. Release.

[cit47] Herve F., Ghinea N., Scherrmann J. M. (2008). CNS delivery *via* adsorptive transcytosis. AAPS J..

[cit48] Pancholi B., Choudhary M. K., Kumar M., Babu R., Vora L. K., Khatri D. K., Garabadu D. (2025). Cell-penetrating proteins and peptides as a promising theragnostic agent for neurodegenerative disorder. J. Drug Delivery Sci. Technol..

[cit49] Mendes M., Sousa J. J., Pais A., Vitorino C. (2018). Targeted Theranostic Nanoparticles for Brain Tumor Treatment. Pharmaceutics.

[cit50] Neves V., Aires-da-Silva F., Morais M., Gano L., Ribeiro E., Pinto A., Aguiar S., Gaspar D., Fernandes C., Correia J. D. G., Castanho M. (2017). Novel Peptides Derived from Dengue Virus Capsid Protein Translocate Reversibly the Blood–Brain Barrier through a Receptor-Free Mechanism. ACS Chem. Biol..

[cit51] Cavaco M., Valle J., da Silva R., Correia J. D. G., Castanho M., Andreu D., Neves V. (2020). (D)PepH3, an Improved Peptide Shuttle for Receptor-independent Transport Across the Blood–Brain Barrier. Curr. Pharm. Des..

[cit52] Cavaco M., Perez-Peinado C., Valle J., Silva R. D. M., Gano L., Correia J. D. G., Andreu D., Castanho M., Neves V. (2024). The use of a selective, nontoxic dual-acting peptide for breast cancer patients with brain metastasis. Biomed. Pharmacother..

[cit53] Cavaco M., Fraga P., Valle J., Silva R. D. M., Gano L., Correia J. D. G., Andreu D., Castanho M., Neves V. (2024). Molecular determinants for brain targeting by peptides: a meta-analysis approach with experimental validation. Fluids Barriers CNS.

[cit54] Benešová M., Umbricht C. A., Schibli R., Müller C. (2018). Albumin-Binding PSMA Ligands: Optimization of the Tissue Distribution Profile. Mol. Pharm..

[cit55] Raheem S. J., Salih A. K., Garcia M. D., Sharpe J. C., Toosi B. M., Price E. W. (2023). A Systematic Investigation into the Influence of Net Charge on the Biological Distribution of Radiometalated Peptides Using [(68)Ga]Ga-DOTA-TATE Derivatives. Bioconjug. Chem..

[cit56] Deiser S., Fenzl S., Konig V., Drexler M., Smith L. M., George M. E., Beck R., Witney T. H., Inoue S., Casini A. (2024). SiFA)SeFe: A Hydrophilic Silicon-Based Fluoride Acceptor Enabling Versatile Peptidic Radiohybrid Tracers. J. Med. Chem..

[cit57] Wurzer A., Parzinger M., Konrad M., Beck R., Gunther T., Felber V., Farber S., Di Carlo D., Wester H. J. (2020). Preclinical comparison of four [(18)F, (nat)Ga]rhPSMA-7 isomers: influence of the stereoconfiguration on pharmacokinetics. EJNMMI Res..

[cit58] Pajouhesh H., Lenz G. R. (2005). Medicinal Chemical Properties of Successful Central Nervous System Drugs. NeuroRx.

[cit59] Brandt M., Cardinale J., Giammei C., Guarrochena X., Happl B., Jouini N., Mindt T. L. (2019). Mini-review: Targeted radiopharmaceuticals incorporating reversible, low molecular weight albumin binders. Nucl. Med. Biol..

[cit60] Holzmann K. L. K., Wolf J. L., Stangl S., Lennartz P., Kasajima A., Mogler C., Haller B., Ebert E. V., Jira D., Lauterbach M. L. A., von Meyer F., Stark L., Mauch L., Schmidl B., Wollenberg B., Multhoff G., Wirth M. (2024). Improved ex vivo fluorescence imaging of human head and neck cancer using the peptide tracer TPP-IRDye800 targeting membrane-bound Hsp70 on tumor cells. Br. J. Cancer.

[cit61] Woods B., Silva R. D. M., Schmidt C., Wragg D., Cavaco M., Neves V., Ferreira V. F. C., Gano L., Morais T. S., Mendes F., Correia J. D. G., Casini A. (2021). Bioconjugate Supramolecular Pd(2+) Metallacages Penetrate the Blood–Brain Barrier *In Vitro* and *In Vivo*. Bioconjug. Chem..

[cit62] Jagtiani E., Yeolekar M., Naik S., Patravale V. (2022). In vitro blood–brain barrier models: An overview. J. Contr. Release.

[cit63] Li G., Simon M. J., Cancel L. M., Shi Z. D., Ji X., Tarbell J. M., Morrison 3rd B., Fu B. M. (2010). Permeability of endothelial and astrocyte cocultures: *in vitro* blood–brain barrier models for drug delivery studies. Ann. Biomed. Eng..

[cit64] Banks K. P., Peacock J. G., Clemenshaw M. N., Kuo P. H. (2019). Optimizing the Diagnosis of Parkinsonian Syndromes With (123)I-Ioflupane Brain SPECT. AJR Am. J. Roentgenol..

[cit65] Sarko D., Beijer B., Garcia Boy R., Nothelfer E. M., Leotta K., Eisenhut M., Altmann A., Haberkorn U., Mier W. (2010). The pharmacokinetics of cell-penetrating peptides. Mol. Pharm..

[cit66] Katsanos A. H., Alexiou G. A., Fotopoulos A. D., Jabbour P., Kyritsis A. P., Sioka C. (2019). Performance of 18F-FDG, 11C-Methionine, and 18F-FET PET for Glioma Grading: A Meta-analysis. Clin. Nucl. Med..

[cit67] Pasi F., Persico M. G., Marenco M., Vigorito M., Facoetti A., Hodolic M., Nano R., Cavenaghi G., Lodola L., Aprile C. (2020). Effects of Photons Irradiation on (18)F-FET and (18)F-DOPA Uptake by T98G Glioblastoma Cells. Front. Neurosci..

[cit68] Qiao Y., He Y., Zhang S., Li G., Liu H., Xu J., Wang X., Qi C., Peng C. (2009). Synthesis and evaluation of novel F-18 labeled fluoroarylvaline derivatives: potential PET imaging agents for tumor detection. Bioorg. Med. Chem. Lett..

[cit69] Geenen L., Nonnekens J., Konijnenberg M., Baatout S., De Jong M., Aerts A. (2021). Overcoming nephrotoxicity in peptide receptor radionuclide therapy using [(177)Lu]Lu-DOTA-TATE for the treatment of neuroendocrine tumours. Nucl. Med. Biol..

[cit70] Weineisen M., Schottelius M., Simecek J., Baum R. P., Yildiz A., Beykan S., Kulkarni H. R., Lassmann M., Klette I., Eiber M., Schwaiger M., Wester H. J. (2015). 68Ga- and 177Lu-Labeled PSMA I&T: Optimization of a PSMA-Targeted Theranostic Concept and First Proof-of-Concept Human Studies. J. Nucl. Med..

[cit71] Scasnár V., van Lier J. E. (1993). The use of SEP-PAK SI cartridges for the preparation of gallium chloride from the citrate solution. Eur. J. Nucl. Med..

[cit72] Costa-Tuna A., Chaves O. A., Loureiro R. J. S., Pinto S., Pina J., Serpa C. (2024). Interaction between a water-soluble anionic porphyrin and human serum albumin unexpectedly stimulates the aggregation of the photosensitizer at the surface of the albumin. Int. J. Biol. Macromol..

