# Supplementary material for: Chimeric peptide-based radiopharmaceuticals for glioblastoma imaging and therapy by targeting mHsp70 and enhancing BBB penetration
Source: Chem Sci. 2026 Jun 3;17(29):14222–36. doi: 10.1039/d6sc00011h (PMC13267484; doi:10.1039/d6sc00011h)
Supplement: SC-017-D6SC00011H-s001 [file SC-017-D6SC00011H-s001.pdf]

Supplementary Information (SI) for Chemical Science.
This journal is © The Royal Society of Chemistry 2026

Supplementary Information Available

Chimeric peptide-based radiopharmaceuticals for glioblastoma imaging and therapy by targeting mHsp70 and enhancing BBB penetration

Franziska Schuderer,[1] Rúben D. M. Silva,[2] Catarina I. G. Pinto,[2] Lena Koller,[3] Stefan Stangl,[3] Lurdes Gano,[2,4] Marco Cavaco,[5,6] Miguel A. R. Castanho,[5,6] Filipa Mendes,[2,4,*] Susanne Kossatz,[3,*] João D. G. Correia,[2,4,*] Angela Casini[1,*]

[1] *Chair of Medicinal and Bioinorganic Chemistry, Department of Chemistry, School of Natural Sciences, Technical University of Munich, Lichtenbergstrasse 4, 85748 Garching bei München, Germany*

[2] *Centro de Ciências e Tecnologias Nucleares, Instituto Superior Técnico, Universidade de Lisboa, CTN, Estrada Nacional 10, 2695-066 Bobadela, LRS, Portugal.*

[3] *Department of Nuclear Medicine, TUM University Hospital, Central Institute for Translational Cancer Research (TranslaTUM), School of Medicine and Health, Technical University of Munich, 81675 Munich, Germany.*

[4] *Departamento de Engenharia e Ciências Nucleares, Instituto Superior Técnico, Universidade de Lisboa, Estrada Nacional 10, 2695-066 Bobadela, LRS, Portugal.*

[5] *Fundação GIMM – Gulbenkian Institute for Molecular Medicine, Avenida Professor Egas Moniz, 1649–028, Lisboa, Portugal*

[6] *Instituto de Bioquímica, Faculdade de Medicina, Universidade de Lisboa, Avenida Professor Egas Moniz, 1649–028, Lisboa, Portugal*

Abbreviations

AA, amino acid; AMT, adsorptive-mediated transcytosis; BBB, blood–brain barrier; CMT, carrier-mediated transcytosis; CO_2, carbon dioxide; CPP, cell-penetrating peptides; CXCR4, chemokine receptor 4; DEN2C, Dengue virus type 2 capsid protein; DMEM, Dulbecco's modified Eagle's medium; DMF, dimethylformamide; DMSO, dimethyl sulfoxide; DIEA, N,N-diisopropylethylamine; DOTA, dodecane tetraacetic acid; EtOH, ethanol; FBS, fetal bovine serum; FD, fluorescently labeled dextran; FITC, fluorescein 5-isothiocyanat; GRPR, gastrin-releasing peptide receptor; HCl, hydrochloric acid; HOAt, 1-hydroxy-7-azabenzotriazole; HR-ESI-MS, high-resolution electrospray ionization mass spectrometry; HSA, human serum albumin; Hz, hertz; m/z, mass-to-charge; MeCN, acetonitrile; MeOH, methanol; PBS, phosphate buffered saline; PepH3, peptide designed to cross BBB; ppm, parts per million; PSMA, prostate-specific membrane antigen; RCP, radio-chemical purity; RMT, receptor-mediated transcytosis; RP-HPLC, reverse-phase high-performance liquid chromatography; SPECT, single-photon emission computed tomography; SPPS, solid-phase peptide synthesis; SSTR, somatostatin receptor type 2; TBTU, 2-(1H-benzotriazole-1-yl)-1,1,3,3-tetramethylaminium tetrafluoroborate; TFA, trifluoroacetic acid; TIPS, triisopropyl silane; TPP, tumor penetrating peptide designed to target mHsp70; TRT, targeted radionuclide therapy.

Table of Contents

Synthesis and Characterization 4

RP-HPLC Chromatograms and (HR-)ESI-MS Spectra of Quality Controls 9

Radio-RP-HPLC and Radio-TLC Chromatograms of Radioactive Labeling 24

In Silico Prediction of BBB-Translocation...33

Stability Studies...34

Flow Cytometry Analysis..36

Biodistribution and Metabolic Studies 37

Synthesis and Characterization

PepH3-1: DOTA-D-γ-Glu-D-PepH3-NH$_2$

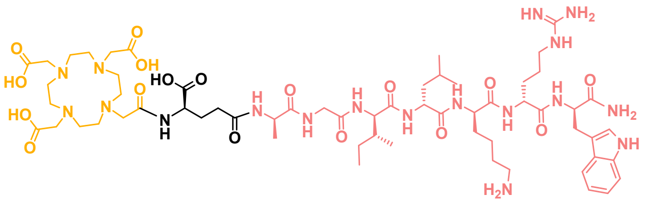

Chemical Formula: $C_{61}H_{100}N_{18}O_{17}$
Molecular Weight: 1357,58 g/mol

PepH3-1 was synthesized via automated SPPS at the rink amide ProTide resin, except for the DOTA coupling, which is done manually. After cleavage off the resin with simultaneous cleavage of the protection groups, the crude peptide was purified via RP-HPLC, yielding 17.0 mg (12.5 mmol, 25%) pure PepH3-1 as a white powder.

Analytical RP-HPLC (10 → 60% B, 15 min, 1 mL/min, MultoKrom® 100-5 C18): t_R = 7.8 min.

MS (ESI, positive): Calc. monoisotopic mass ($C_{61}H_{100}N_{18}O_{17}$): 1356.75 g/mol; found: m/z = 679.1 $[M+2H]^{2+}$, 1358.8 $[M+H]^{+}$.

HR-MS (ESI, positive): Calc. monoisotopic mass ($C_{61}H_{100}N_{18}O_{17}$): 1356.75 g/mol; found: m/z = 679.4 $[M+2H]^{2+}$, 1358.8 $[M+H]^{+}$.

Complexation with natural lutetium was carried out following, and $[^{nat}Lu]$Lu-PepH3-1 was obtained.

Analytical RP-HPLC (10 → 50% B, 15 min, 1 mL/min, MultoKrom® 100-5 C18): t_R = 9.3 min.

MS (ESI, positive): Calc. monoisotopic mass ($C_{61}H_{97}LuN_{18}O_{17}$): 1528.67 g/mol; found: m/z = 764.6 $[M+2H]^{2+}$, 1526.8 $[M+H]^{+}$.

PepH3-2: DOTA-PEG$_6$-D-PepH3-NH$_2$

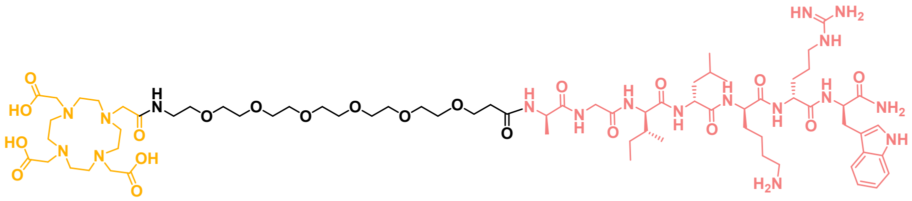

Chemical Formula: $C_{71}H_{122}N_{18}O_{21}$
Molecular Weight: 1563,86 g/mol

PepH3-2 was synthesized via automated SPPS at the rink amide ProTide resin, except for the PEG$_6$ and DOTA coupling, which are done manually. After cleavage off the resin with simultaneous cleavage of the protection groups, the crude peptide was purified via RP-HPLC, yielding 25.2 mg (16.1 mmol, 32%) pure PepH3-2 as a white powder.

Analytical RP-HPLC (10 → 50% B, 15 min, 1 mL/min, MultoKrom® 100-5 C18): t_R = 10.2 min.

MS (ESI, positive): Calc. monoisotopic mass ($C_{71}H_{122}N_{18}O_{21}$): 1562.90 g/mol; found: m/z = 522.3 $[M+3H]^{3+}$, 782.5 $[M+2H]^{2+}$.
HR-MS (ESI, positive): Calc. monoisotopic mass ($C_{71}H_{122}N_{18}O_{21}$): 1562.90 g/mol; found: m/z = 782.5 $[M+2H]^{2+}$, 1563.9 $[M+H]^{+}$.

Complexation with natural lutetium was carried out following, and [^{nat}Lu]Lu-PepH3-2 was obtained.
Analytical RP-HPLC (10 → 50% B, 15 min, 1 mL/min, MultoKrom® 100-5 C18): t_R = 10.8 min.
MS (ESI, positive): Calc. monoisotopic mass ($C_{71}H_{119}LuN_{18}O_{21}$): 1734.82 g/mol; found: m/z = 578.6 $[M+3H]^{3+}$, 867.2 $[M+2H]^{2+}$, 1156.3 $[2M+3H]^{3+}$, 1733.1 $[M+H]^{+}$.

TPP-1: DOTA-D-Glu-L-TPP-OH

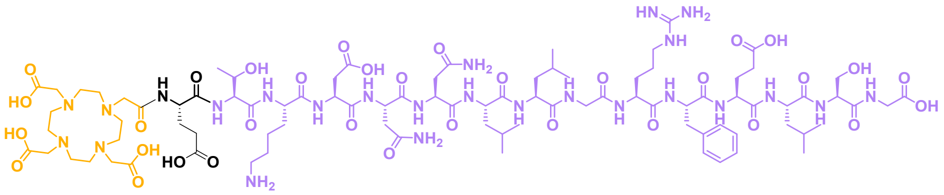

Chemical Formula: $C_{88}H_{143}N_{25}O_{33}$
Molecular Weight: 2079,25 g/mol

TPP-1 was synthesized via manual SPPS at the 2-CTC resin. After cleavage off the resin with simultaneous cleavage of the protection groups, the crude peptide was purified via RP-HPLC, yielding 4.1 mg (2.0 µmol, 4%) pure TPP-1 as a white powder.
Analytical RP-HPLC (10 → 50% B, 15 min, 1 mL/min, MultoKrom® 100-5 C18): t_R = 10.1 min.
MS (ESI, positive): Calc. monoisotopic mass ($C_{88}H_{143}N_{25}O_{33}$): 2078.03 g/mol; found: m/z = 692.7 $[M+3H]^{3+}$, 1038.7 $[M+2H]^{2+}$, 1358.74 $[2M+3H]^{3+}$, 1558.2 $[3M+4H]^{4+}$.
HR-MS (ESI, positive): Calc. monoisotopic mass ($C_{88}H_{143}N_{25}O_{33}$): 2078.03 g/mol; found: m/z = 694.0 $[M+3H]^{3+}$, 1040.5 $[M+2H]^{2+}$.

Complexation with natural lutetium was carried out following, and [^{nat}Lu]Lu-TPP-1 was obtained.
Analytical RP-HPLC (10 → 50% B, 15 min, 1 mL/min, MultoKrom® 100-5 C18): t_R = 9.5 min.
MS (ESI, positive): Calc. monoisotopic mass ($C_{88}H_{140}LuN_{25}O_{33}$): 2249.95 g/mol; found: m/z = 749.6 $[M+3H]^{3+}$, 1123.2 $[M+2H]^{2+}$, 1498.1 $[2M+3H]^{3+}$, 1684.0 $[3M+4H]^{4+}$.

TPP-2: DOTA-PEG$_6$-L-TPP-OH

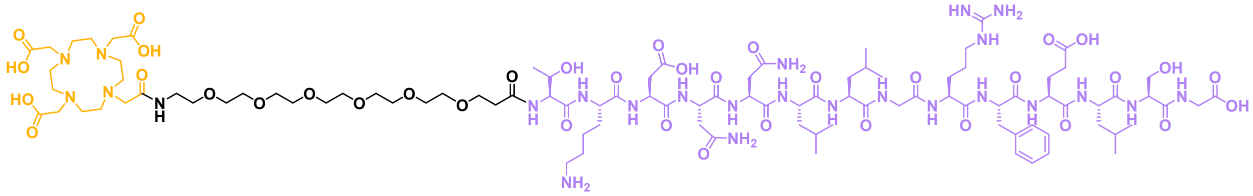

Chemical Formula: $C_{98}H_{165}N_{25}O_{37}$
Molecular Weight: 2285,54 g/mol

TPP-2 was synthesized via manual SPPS at the 2-CTC resin. After cleavage off the resin with simultaneous cleavage of the protection groups, the crude peptide was purified via RP-HPLC, yielding 6.9 mg (3.0 µmol, 6%) pure TPP-2 as a white powder.

Analytical RP-HPLC (10 → 50% B, 15 min, 1 mL/min, MultoKrom® 100-5 C18): t_R = 10.2 min.

MS (ESI, positive): Calc. monoisotopic mass ($C_{98}H_{165}N_{25}O_{37}$): 2284.18 g/mol; found: m/z = 762.1 $[M+3H]^{3+}$, 1142.2 $[M+2H]^{2+}$.

HR-MS (ESI, positive): Calc. monoisotopic mass ($C_{98}H_{165}N_{25}O_{37}$): 2284.18 g/mol; found: m/z = 762.4 $[M+3H]^{3+}$, 775.4 $[M+2H+K]^{3+}$, 1143.1 $[M+2H]^{2+}$, 1162.6 $[M+H+K]^{2+}$.

Complexation with natural lutetium was carried out following, and $[^{nat}Lu]$Lu-TPP-2 was obtained.

Analytical RP-HPLC (15 → 45% B, 15 min, 1 mL/min, MultoKrom® 100-5 C18): t_R = 9.5 min.

MS (ESI, positive): Calc. monoisotopic mass ($C_{98}H_{162}LuN_{25}O_{37}$): 2456.10 g/mol; found: m/z = 818.4 $[M+3H]^{3+}$, 1226.6 $[M+2H]^{2+}$, 1635.6 $[2M+3H]^{3+}$.

Comb-1: DOTA-D-PepH3-L-TPP-OH

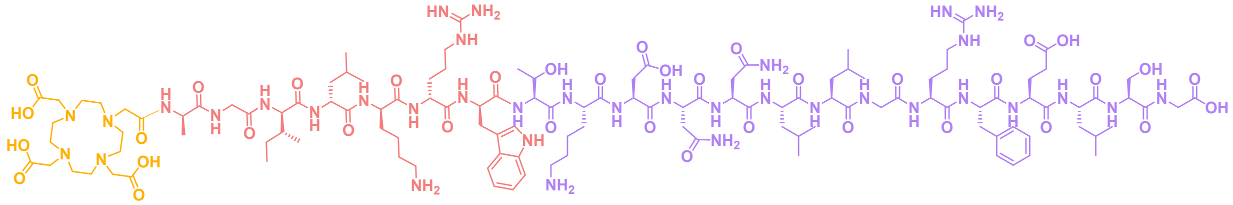

Chemical Formula: $C_{123}H_{200}N_{36}O_{37}$
Molecular Weight: 2775,17 g/mol

Comb-1 was synthesized via manual SPPS at the 2-CTC resin. After cleavage off the resin with simultaneous cleavage of the protection groups, the crude peptide was purified via RP-HPLC, yielding 11.0 mg (4.0 µmol, 8%) pure Comb-1 as a white powder.

Analytical RP-HPLC (10 → 50% B, 15 min, 1 mL/min, MultoKrom® 100-5 C18): t_R = 11.0 min.

MS (ESI, positive): Calc. monoisotopic mass ($C_{123}H_{200}N_{36}O_{37}$): 2773.49 g/mol; found: m/z = 695.0 $[M+4H]^{4+}$, 926.1 $[M+3H]^{3+}$, 1386.6 $[M+2H]^{2+}$.

HR-MS (ESI, positive): Calc. monoisotopic mass ($C_{123}H_{200}N_{36}O_{37}$): 2773.49 g/mol; found: m/z = 5559 $[M+5H]^{5+}$, 694.6 $[M+4H]^{4+}$, 925.8 $[M+3H]^{3+}$, 1388.3 $[M+2H]^{2+}$.

Complexation with natural lutetium was carried out, and [^{nat}Lu]Lu-Comb-1 was obtained.
Analytical RP-HPLC (15 → 45% B, 15 min, 1 mL/min, MultoKrom® 100-5 C18): t_R = 11.4 min.
MS (ESI, positive): Calc. monoisotopic mass ($C_{123}H_{197}LuN_{36}O_{37}$): 2945.40 g/mol; found: m/z = 736.5 $[M+4H]^{4+}$, 981.2 $[M+3H]^{3+}$, 1470.8 $[M+2H]^{2+}$.

Comb-2: DOTA-PEG$_6$-D-PepH3-L-TPP-OH

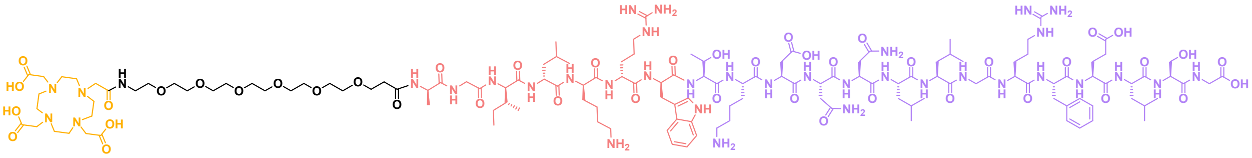

Chemical Formula: $C_{138}H_{229}N_{37}O_{44}$
Molecular Weight: 3110,57 g/mol

Comb-2 was synthesized via manual SPPS at the 2-CTC resin. After cleavage off the resin with simultaneous cleavage of the protection groups, the crude peptide was purified via RP-HPLC, yielding 20.8 mg (6.7 µmol, 13%) pure Comb-2 as a white powder.
Analytical RP-HPLC (10 → 50% B, 15 min, 1 mL/min, MultoKrom® 100-5 C18): t_R = 11.3 min.
MS (ESI, positive): Calc. monoisotopic mass ($C_{138}H_{229}N_{37}O_{44}$): 3108.68 g/mol; found: m/z = 623.1 $[M+5H]^{5+}$, 778.4 $[M+4H]^{4+}$, 1037.2 $[M+3H]^{3+}$.
HR-MS (ESI, positive): Calc. monoisotopic mass ($C_{138}H_{229}N_{37}O_{44}$): 3108.68 g/mol; found: m/z = 778.7 $[M+4H]^{4+}$, 1037.2 $[M+3H]^{3+}$, 1555.3 $[M+2H]^{2+}$.

Complexation with natural lutetium was carried out, and [^{nat}Lu]Lu-Comb-2 was obtained.
Analytical RP-HPLC (15 → 45% B, 15 min, 1 mL/min, MultoKrom® 100-5 C18): t_R = 12.1 min.
MS (ESI, positive): Calc. monoisotopic mass ($C_{138}H_{226}LuN_{37}O_{44}$): 3280.60 g/mol; found: m/z = 819.8 $[M+4H]^{4+}$, 1093.5 $[M+3H]^{3+}$, 1638.1 $[M+2H]^{2+}$.

FITC-TPP-2: FITC-PEG$_6$-L-TPP-OH

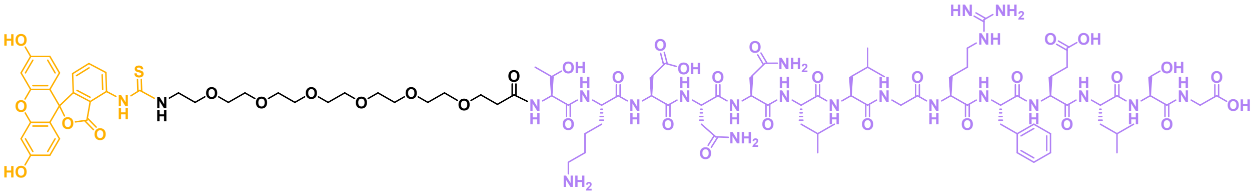

Chemical Formula: $C_{103}H_{150}N_{22}O_{35}S$
Molecular Weight: 2288,51 g/mol

FITC-TPP-2 was synthesized via automated SPPS at the Fmoc-Gly-Wang-ProTide resin. Only the PEG$_6$ linker and FITC isomer 1 were coupled manually. After cleavage off the resin with

simultaneous cleavage of the protection groups, the crude peptide was purified via RP-HPLC, yielding 1.8 mg (0.7 µmol, 1%) pure FITC-TPP-2 as a yellow powder.

Analytical RP-HPLC (10 → 90% B, 15 min, 1 mL/min, MultoKrom® 100-5 C18): t_R = 7.6 min.

MS (ESI, positive): Calc. monoisotopic mass ($C_{103}H_{150}N_{22}O_{35}S$): 2287.04 g/mol; found: m/z = 762.1 $[M+3H]^{3+}$, 1142.5 $[M+2H]^{2+}$, 1522.4 $[2M+3H]^{3+}$, 1712.9 $[3M+4H]^{4+}$.

HR-MS (ESI, positive): Calc. monoisotopic mass ($C_{103}H_{150}N_{22}O_{35}S$): 2287.04 g/mol; found: m/z = 763.7 $[M+3H]^{3+}$, 1142.0 $[M+2H]^{2+}$.

FITC-Comb-2: FITC-PEG$_6$-D-PepH3-L-TPP-OH

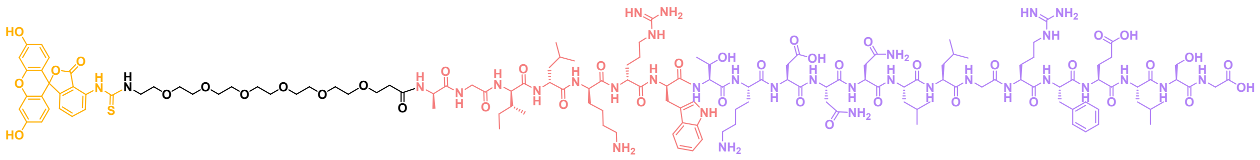

Chemical Formula: $C_{143}H_{214}N_{34}O_{42}S$
Molecular Weight: 3113,54 g/mol

FITC-Comb-2 was synthesized via automated SPPS at the Fmoc-Gly-Wang-ProTide resin. Only the PEG$_6$ linker and FITC isomer 1 were coupled manually. After cleavage off the resin with simultaneous cleavage of the protection groups, the crude peptide was purified via RP-HPLC, yielding 1.9 mg (0.6 µmol, 1%) pure FITC-Comb-2 as a yellow powder.

Analytical RP-HPLC (20 → 60% B, 15 min, 1 mL/min, MultoKrom® 100-5 C18): t_R = 9.4 min.

MS (ESI, positive): Calc. monoisotopic mass ($C_{143}H_{214}N_{34}O_{42}S$): 3111.54 g/mol; found: m/z = 778.8 $[M+4H]^{4+}$, 1038.5 $[M+3H]^{3+}$.

HR-MS (ESI, positive): Calc. monoisotopic mass ($C_{143}H_{214}N_{34}O_{42}S$): 3111.54 g/mol; found: m/z = 779.1 $[M+4H]^{4+}$, 1038.5 $[M+3H]^{3+}$, 1557.3 $[M+2H]^{2+}$.

RP-HPLC Chromatograms and (HR-)ESI-MS Spectra of Quality Controls

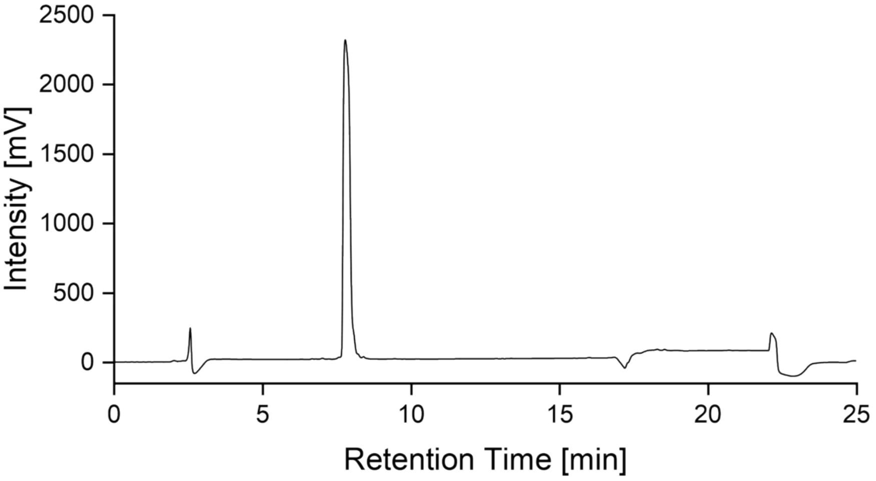

Figure S1. RP-HPLC of the quality control of PepH3-1. With a gradient of 10-60% B in 15 min, the retention time observed was t_R = 7.8 min and the purity: 98%.

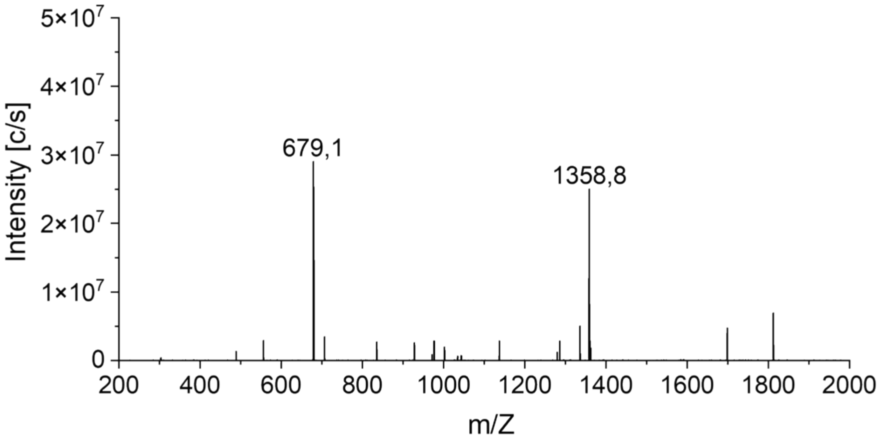

Figure S2. ESI-MS spectrum of the quality control of PepH3-1. Calc. monoisotopic mass ($C_{61}H_{100}N_{18}O_{17}$): 1356.75 g/mol; found: m/z = 679.1 $[M+2H]^{2+}$, 1358.8 $[M+H]^{+}$.

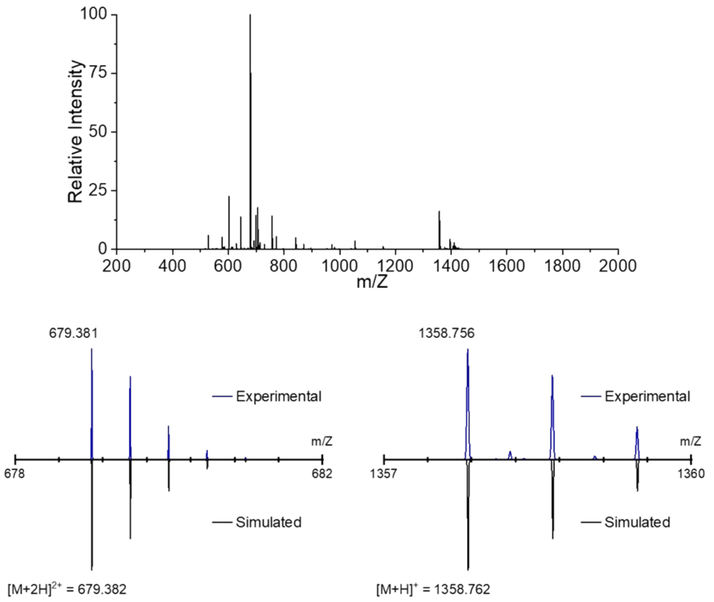

Figure S3. HR-ESI-MS spectrum of PepH3-1.

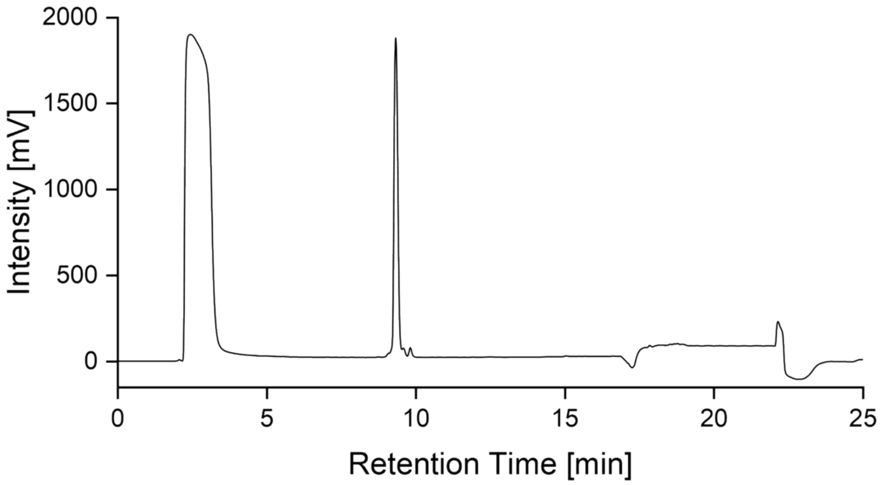

Figure S4. RP-HPLC of the quality control of [natLu]Lu-PepH3-1. With a gradient of 10-50% B in 15 min, the retention time observed was t_R = 9.3 min and the purity: 97%.

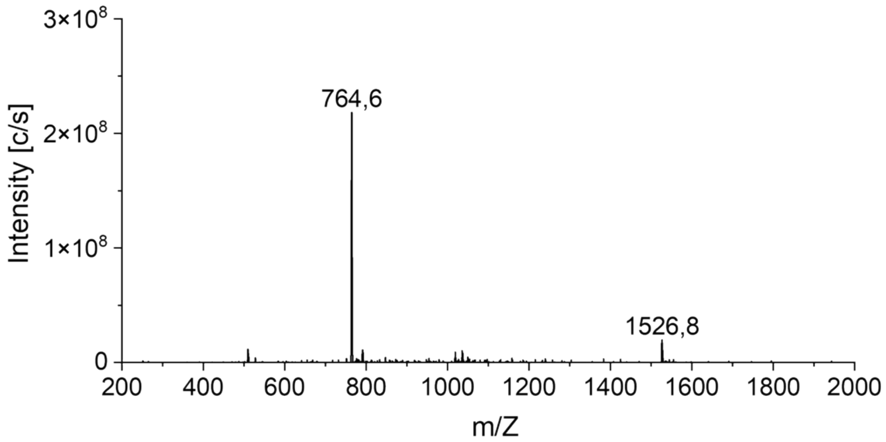

Figure S5. ESI-MS spectrum of the quality control of [natLu]Lu-PepH3-1. Calc. monoisotopic mass ($C_{61}H_{97}LuN_{18}O_{17}$): 1528.67 g/mol; found: m/z = 764.6 $[M+2H]^{2+}$, 1526.8 $[M+H]^{+}$.

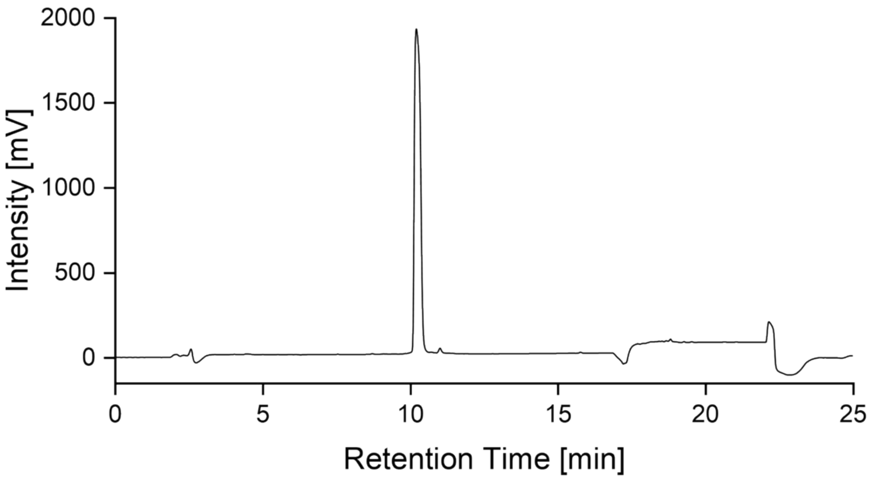

Figure S6. RP-HPLC of the quality control of PepH3-2. With a gradient of 10-50% B in 15 min, the retention time observed was t_R = 10.2 min and the purity: 99%.

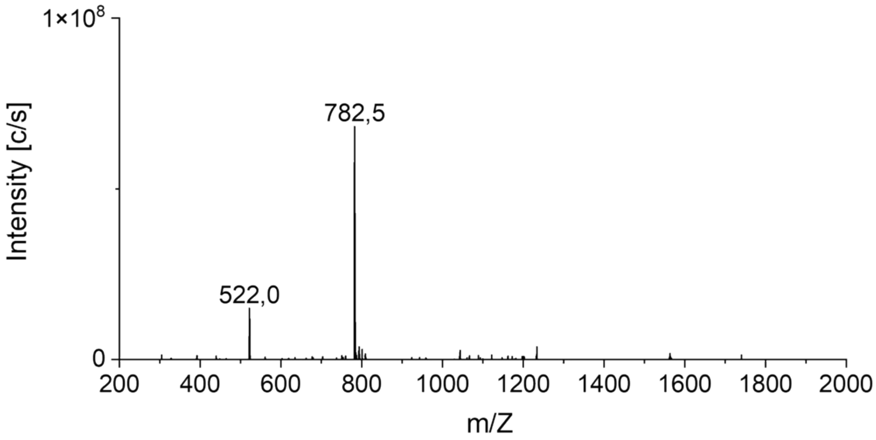

Figure S7. ESI-MS spectrum of the quality control of PepH3-2. Calc. monoisotopic mass ($C_{71}H_{122}N_{18}O_{21}$): 1562.90 g/mol; found: m/z = 522.0 $[M+3H]^{3+}$, 782.5 $[M+2H]^{2+}$.

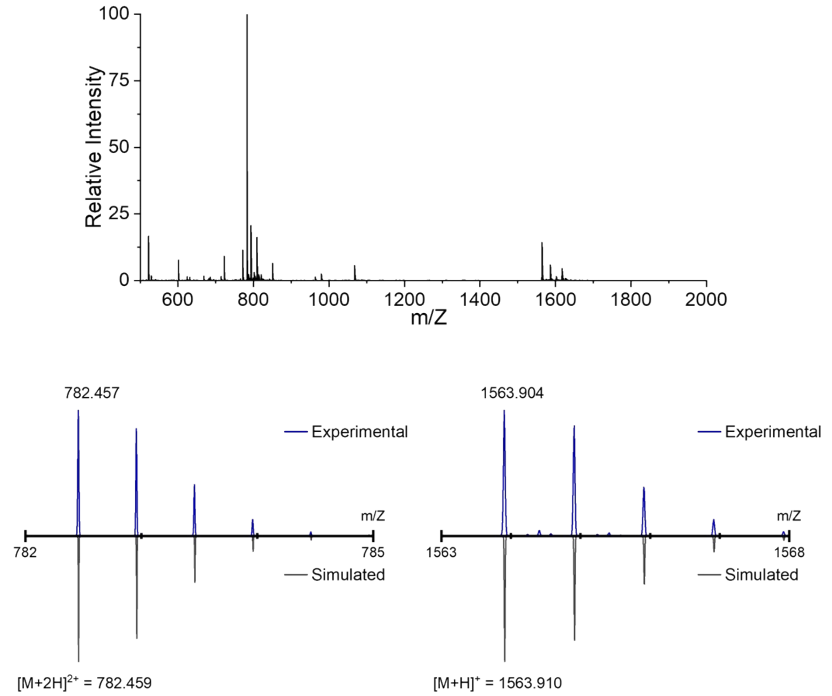

Figure S8. HR-ESI-MS spectrum of PepH3-2.

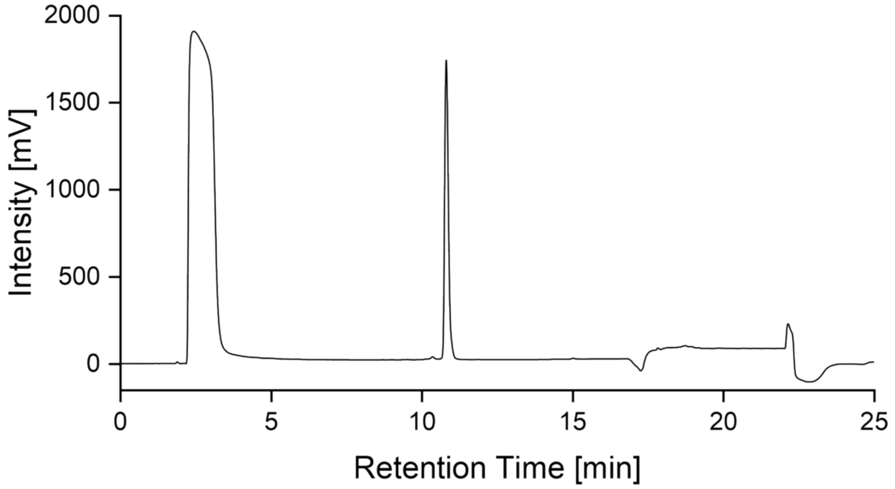

Figure S9. RP-HPLC of the quality control of [^{nat}Lu]Lu-PepH3-2. With a gradient of 10-50% B in 15 min, the retention time observed was t_R = 10.8 min and the purity: 99%.

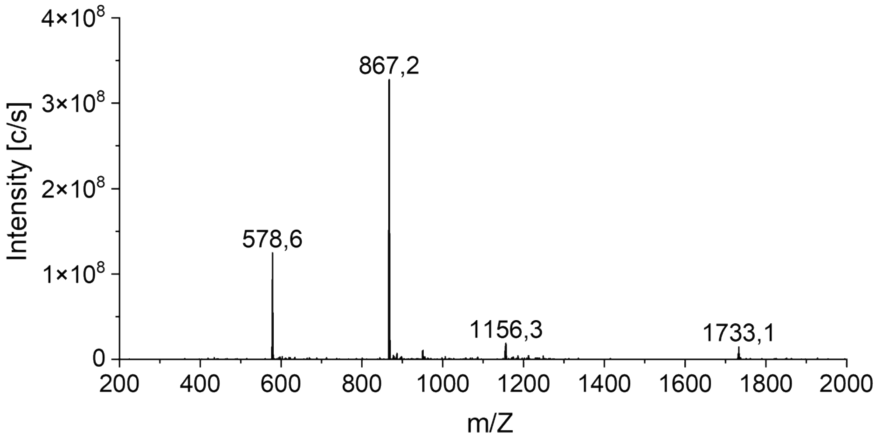

Figure S10. ESI-MS spectrum of the quality control of [natLu]Lu-PepH3-2. Calc. monoisotopic mass ($C_{71}H_{119}LuN_{18}O_{21}$): 1734.82 g/mol; found: m/z = 578.6 $[M+3H]^{3+}$, 867.2 $[M+2H]^{2+}$, 1156.3 $[2M+3H]^{3+}$, 1733.1 $[M+H]^{+}$.

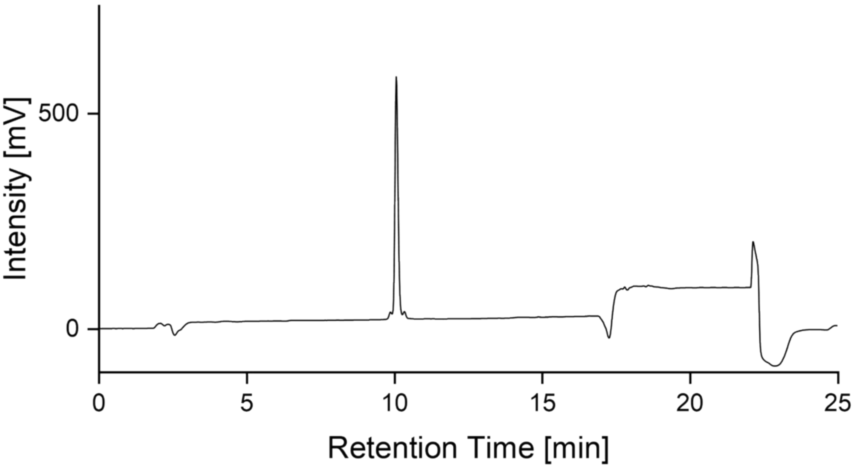

Figure S11. RP-HPLC of the quality control of TPP-1. With a gradient of 10-50% B in 15 min, the retention time observed was t_R = 10.1 min and the purity: 95%.

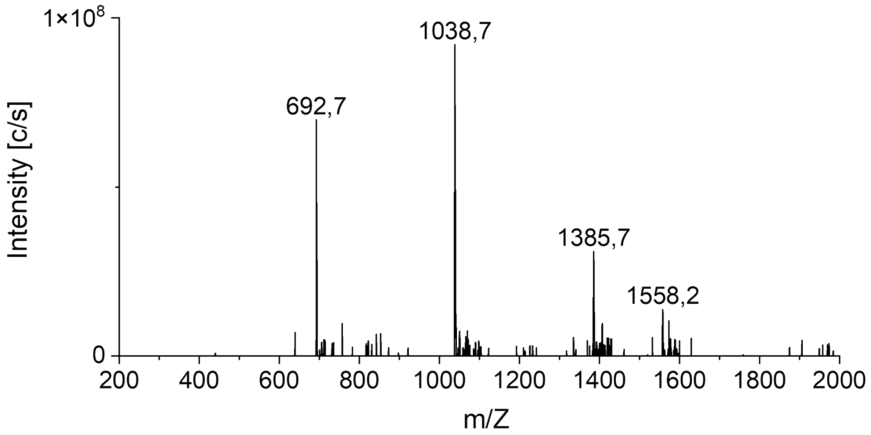

Figure S12. ESI-MS spectrum of the quality control of TPP-1. Calc. monoisotopic mass ($C_{88}H_{143}N_{25}O_{33}$): 2078.03 g/mol; found: m/z = 692.7 $[M+3H]^{3+}$, 1038.7 $[M+2H]^{2+}$, 1358.7 $[2M+3H]^{3+}$, 1558.2 $[3M+4H]^{4+}$.

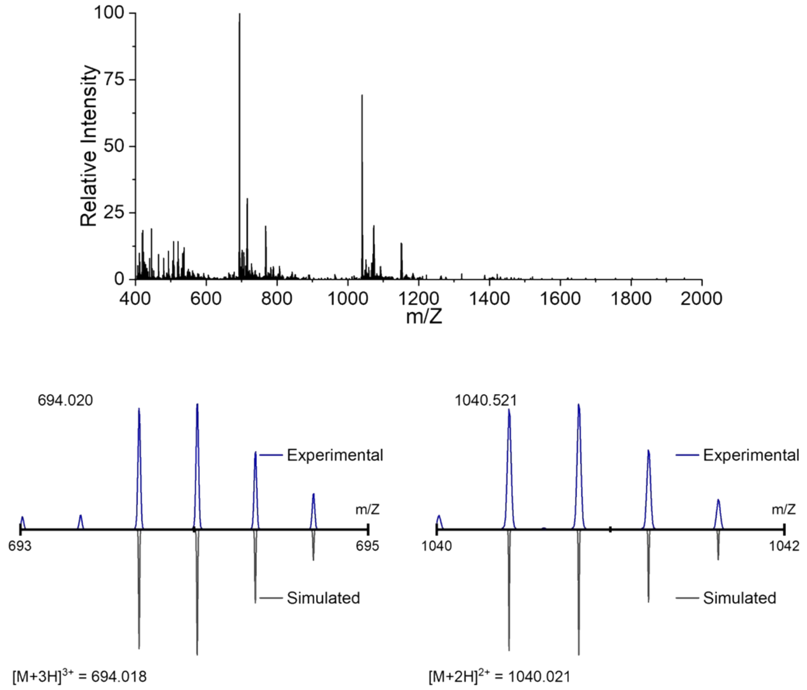

Figure S13. HR-ESI-MS spectrum of TPP-1.

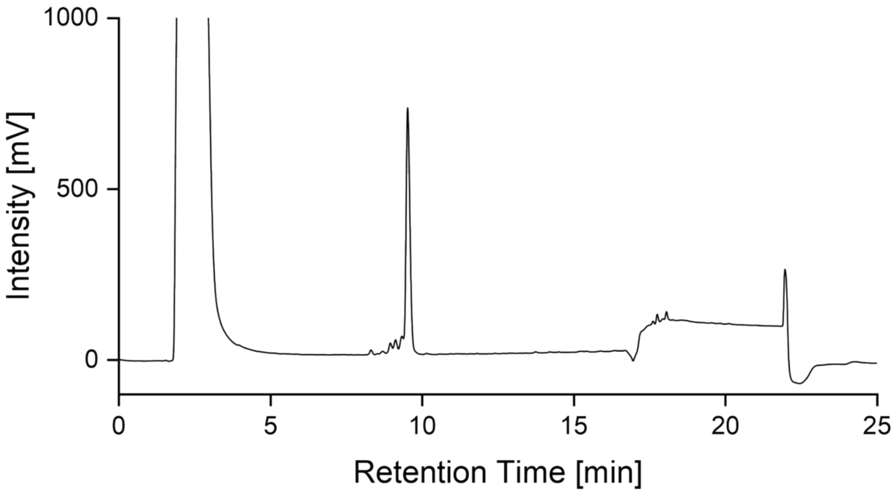

Figure S14. RP-HPLC of the quality control of [natLu]Lu-TPP-1 in DMSO. With a gradient of 10-50% B in 15 min, the retention time observed was t_R = 9.5 min and the purity: 92%.

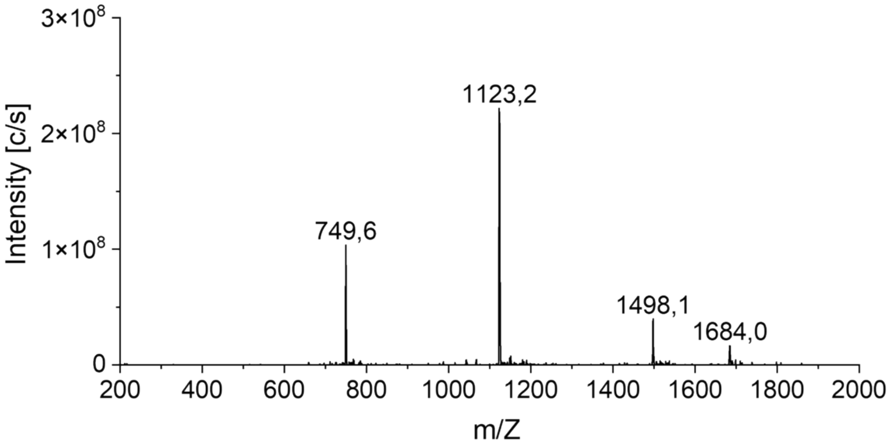

Figure S15. ESI-MS spectrum of the quality control of [natLu]Lu-TPP-1. Calc. monoisotopic mass ($C_{88}H_{140}LuN_{25}O_{33}$): 2249.95 g/mol; found: m/z = 749.6 $[M+3H]^{3+}$, 1123.2 $[M+2H]^{2+}$, 1498.1 $[2M+3H]^{3+}$, 1684.0 $[3M+4H]^{4+}$.

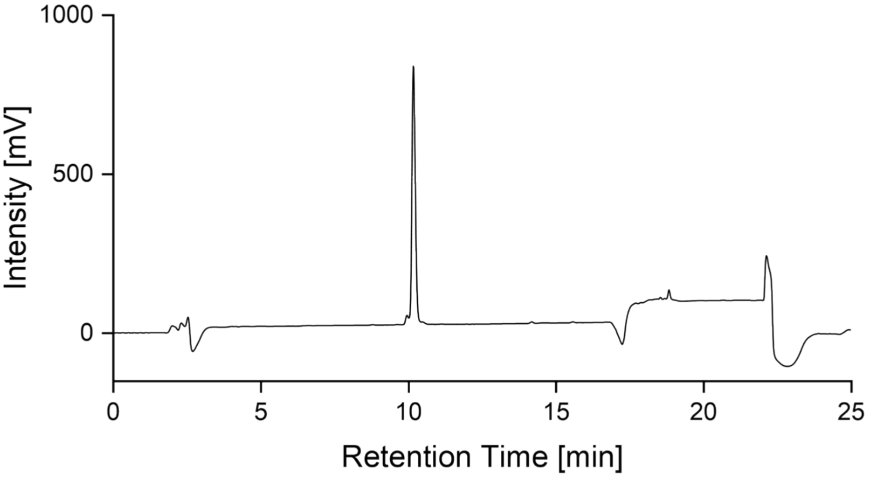

Figure S16. RP-HPLC of the quality control of TPP-2. With a gradient of 10-50% B in 15 min, the retention time observed was t_R = 10.2 min and the purity: 96%.

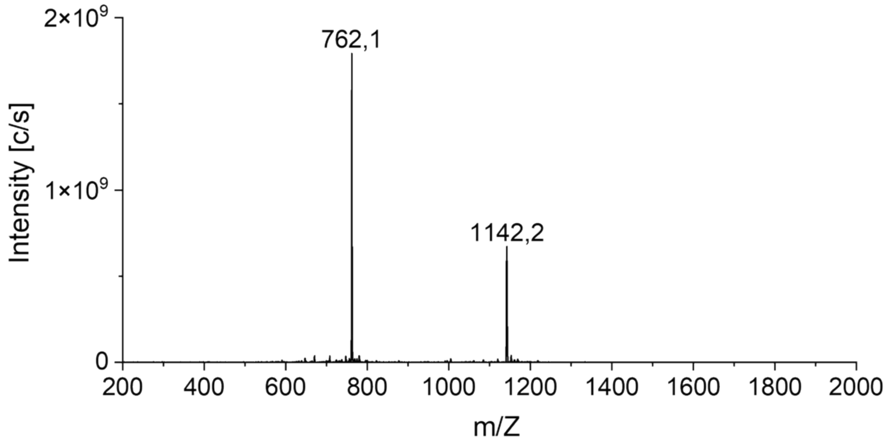

Figure S17. ESI-MS spectrum of the quality control of TPP-2. Calc. monoisotopic mass ($C_{98}H_{165}N_{25}O_{37}$): 2284.18 g/mol; found: m/z = 762.1 $[M+3H]^{3+}$, 1142.2 $[M+2H]^{2+}$.

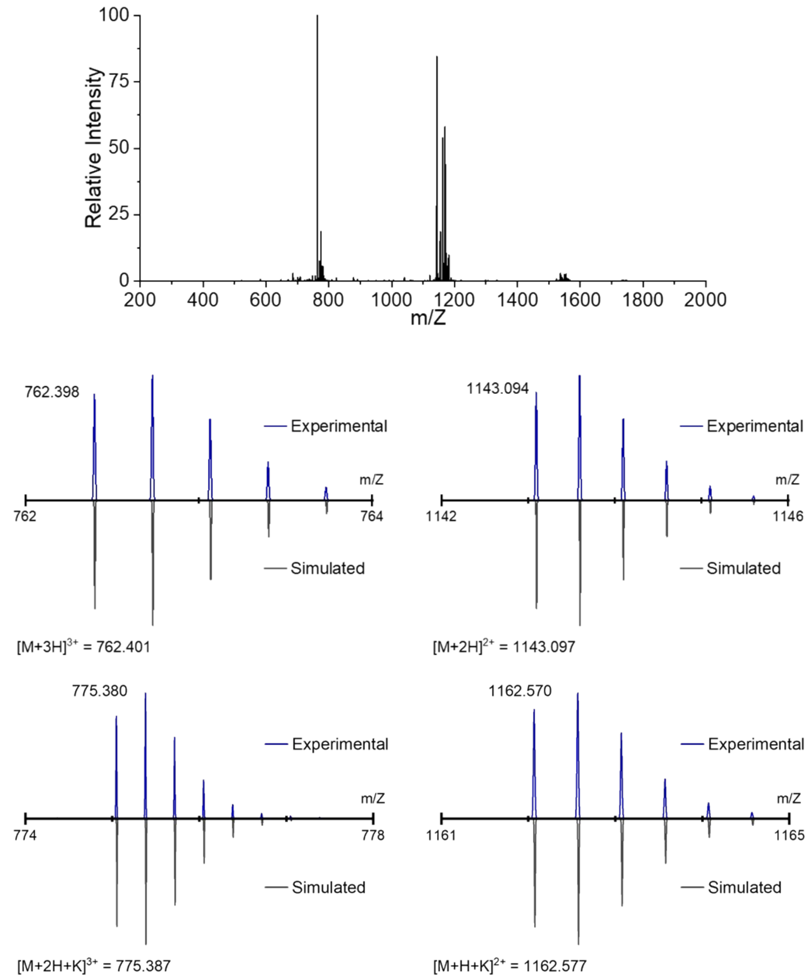

Figure S18. HR-ESI-MS of TPP-2.

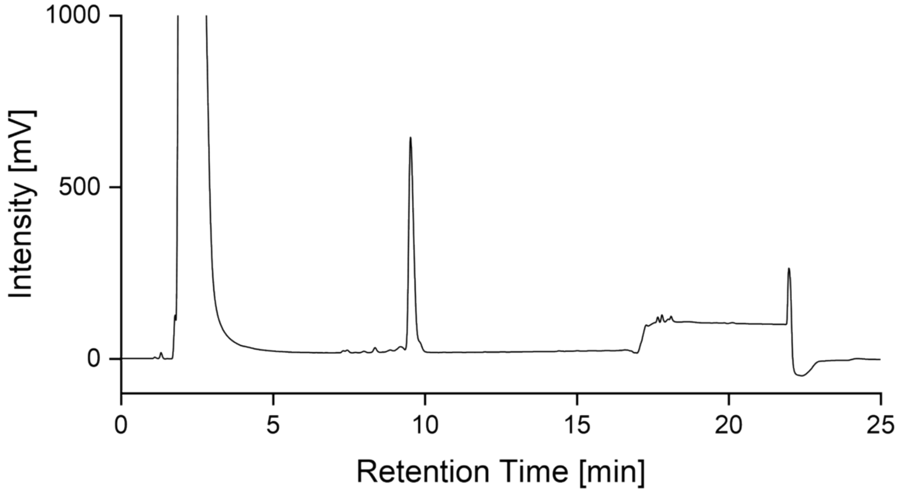

Figure S19. RP-HPLC of the quality control of [^{nat}Lu]Lu-TPP-2 in DMSO. With a gradient of 15-45% B in 15 min, the retention time observed was t_R = 9.5 min and the purity: 95%.

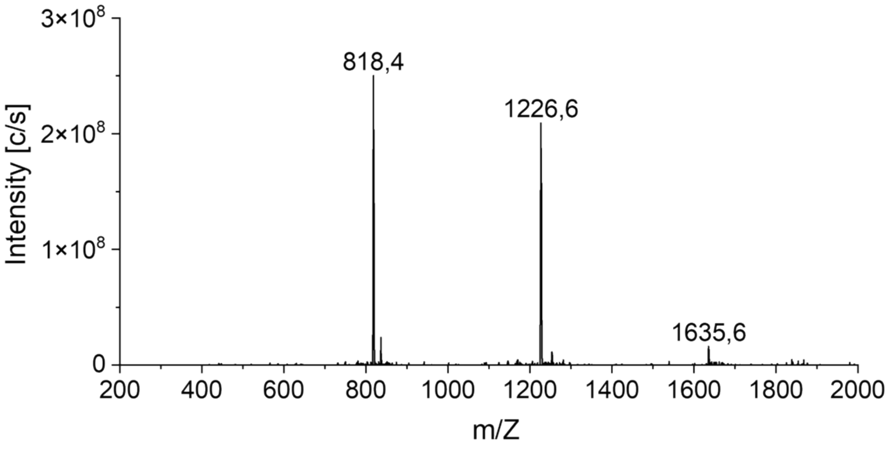

Figure S20. ESI-MS spectrum of the quality control of [natLu]Lu-TPP-2. Calc. monoisotopic mass ($C_{98}H_{162}LuN_{25}O_{37}$): 2456.10 g/mol; found: m/z = 818.4 $[M+3H]^{3+}$, 1226.6 $[M+2H]^{2+}$, 1635.6 $[2M+3H]^{3+}$.

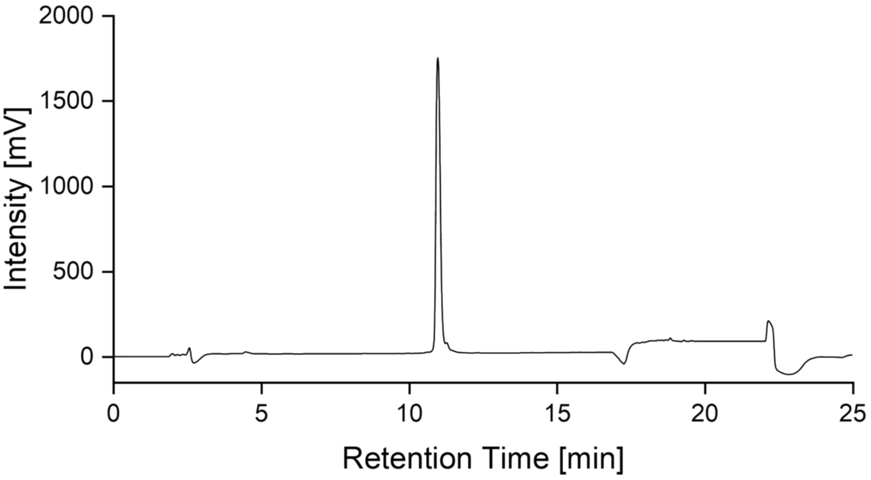

Figure S21. RP-HPLC of the quality control of Comb-1. With a gradient of 10-50% B in 15 min, the retention time observed was t_R = 11.0 min and the purity: 98%.

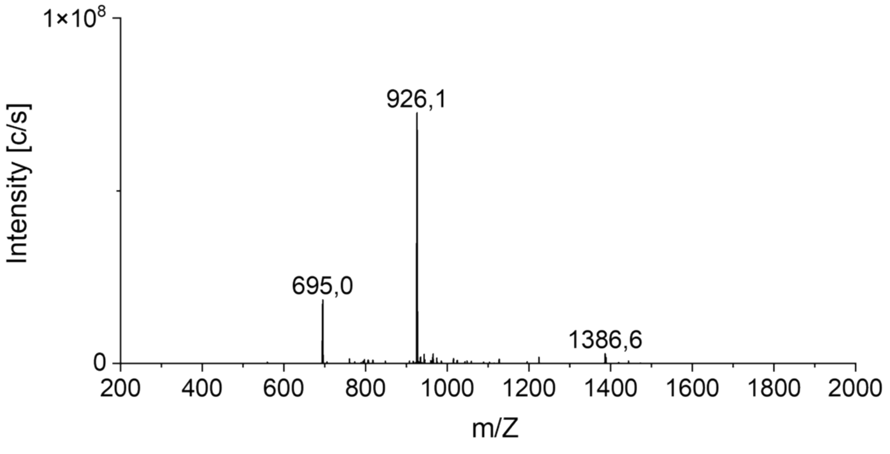

Figure S22. ESI-MS spectrum of the quality control of Comb-1. Calc. monoisotopic mass ($C_{123}H_{200}N_{36}O_{37}$): 2773.49 g/mol; found: m/z = 695.0 $[M+4H]^{4+}$, 926.1 $[M+3H]^{3+}$, 1386.6 $[M+2H]^{2+}$.

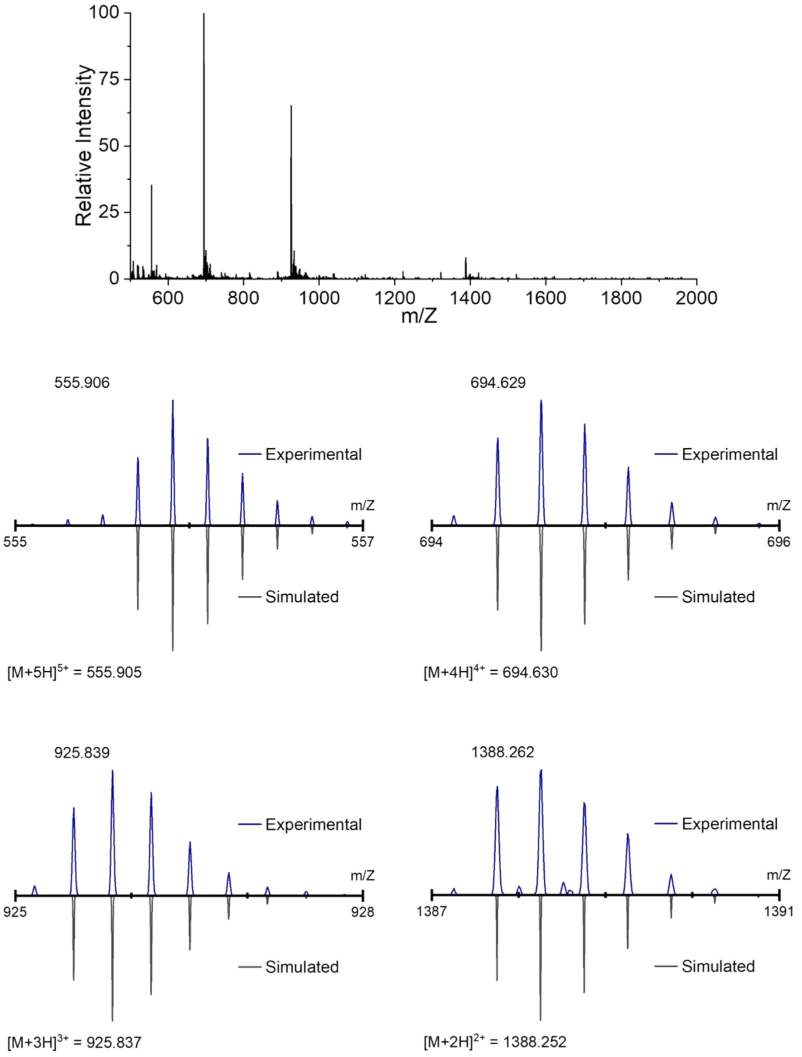

Figure S23. HR-ESI-MS spectrum of Comb-1.

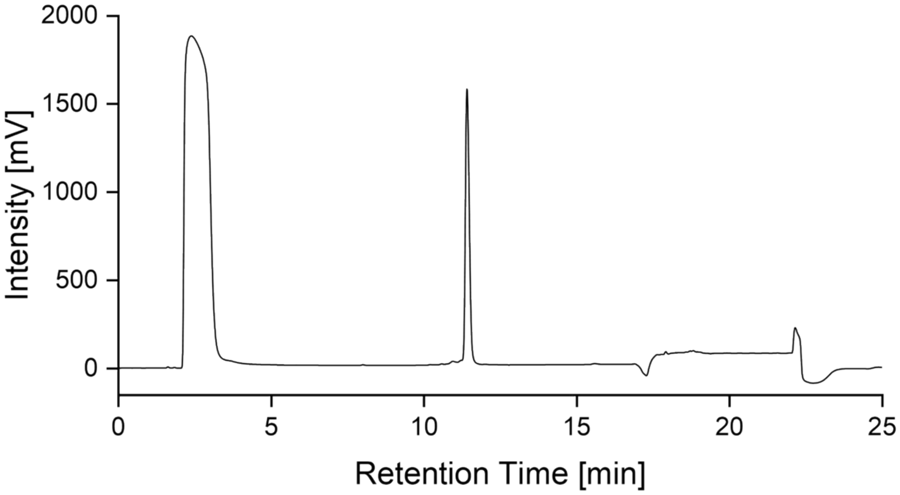

Figure S24. RP-HPLC of the quality control of [^{nat}Lu]Lu-Comb-1 in DMSO. With a gradient of 15-45% B in 15 min, the retention time observed was t_R = 11.4 min and the purity: 97%.

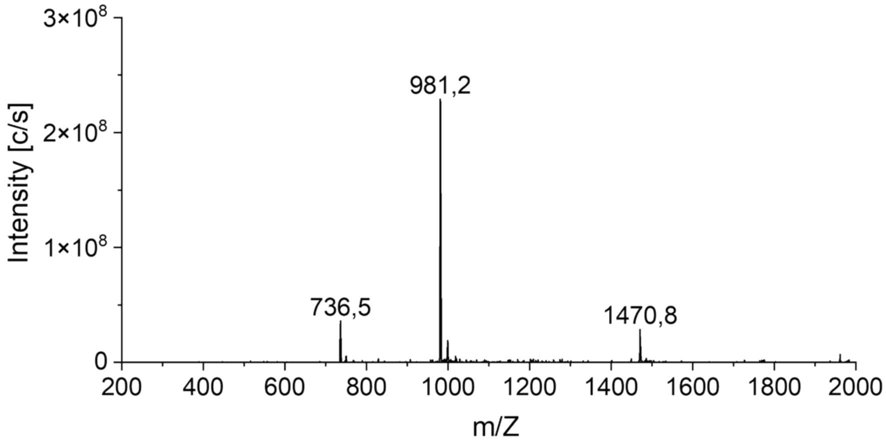

Figure S25. ESI-MS spectrum of the quality control of [natLu]Lu-Comb-1. Calc. monoisotopic mass ($C_{123}H_{197}LuN_{36}O_{37}$): 2945.40 g/mol; found: m/z = 736.5 $[M+4H]^{4+}$, 981.2 $[M+3H]^{3+}$, 1470.8 $[M+2H]^{2+}$.

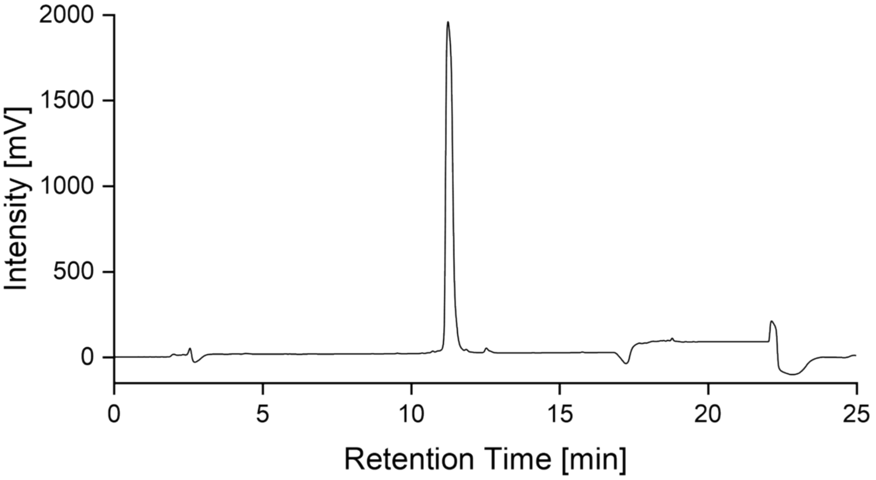

Figure S26. RP-HPLC of the quality control of Comb-2. With a gradient of 10-50% B in 15 min, the retention time observed was t_R = 11.3 min and the purity: 98%.

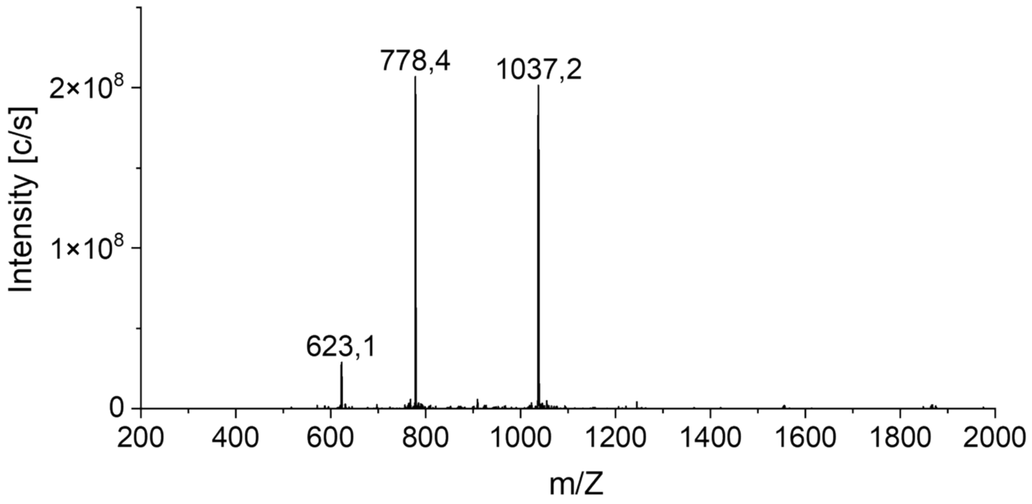

Figure S27. ESI-MS spectrum of the quality control of Comb-2. Calc. monoisotopic mass ($C_{138}H_{229}N_{37}O_{44}$): 3108.68 g/mol; found: m/z = 623.1 $[M+5H]^{5+}$, 778.4 $[M+4H]^{4+}$, 1037.2 $[M+3H]^{3+}$.

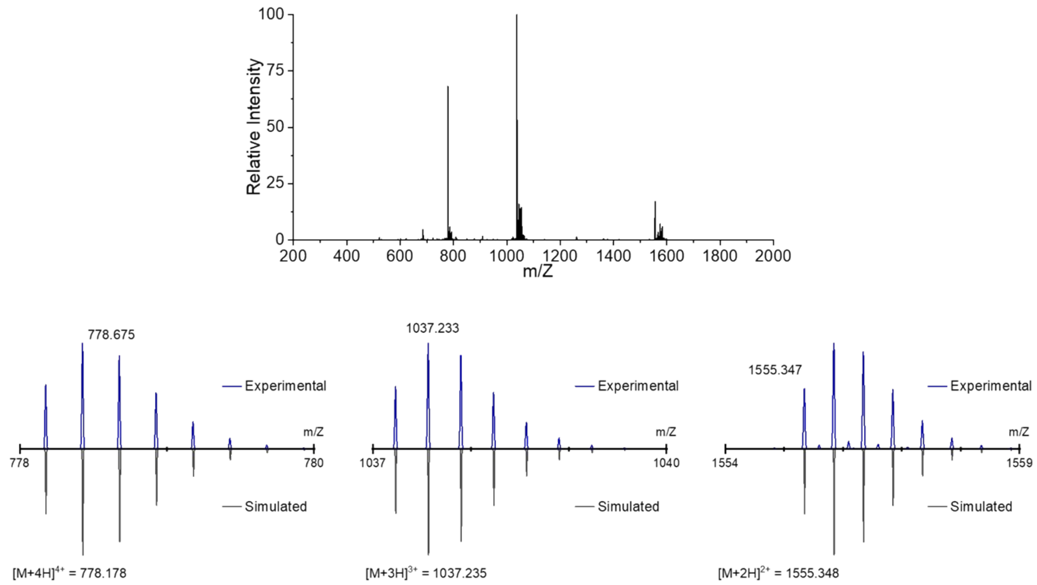

Figure S28. HR-ESI-MS spectrum of Comb-2.

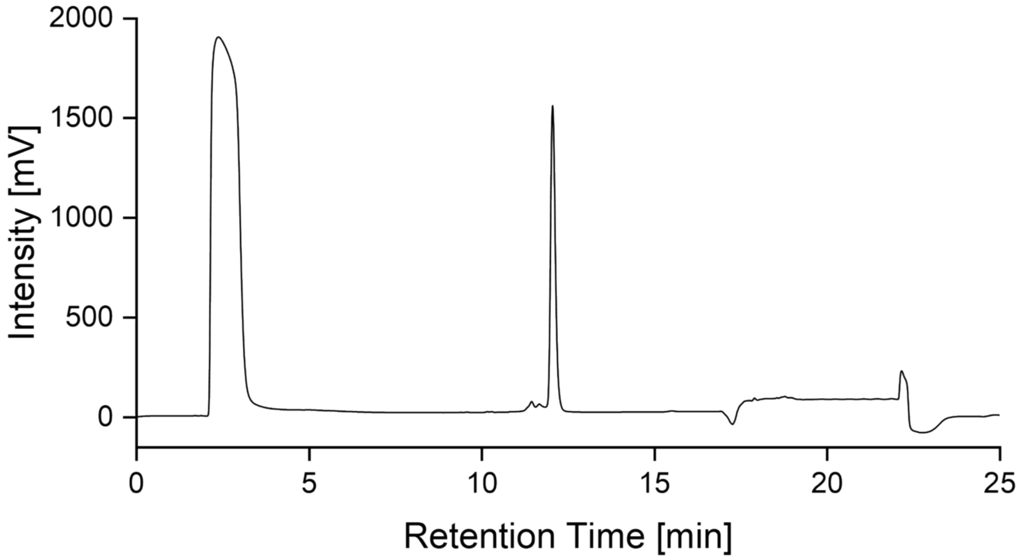

Figure S29. RP-HPLC of the quality control of [^{nat}Lu]Lu-Comb-2 in DMSO. With a gradient of 15-45% B in 15 min, the retention time observed was t_R = 12.1 min and the purity: 93%.

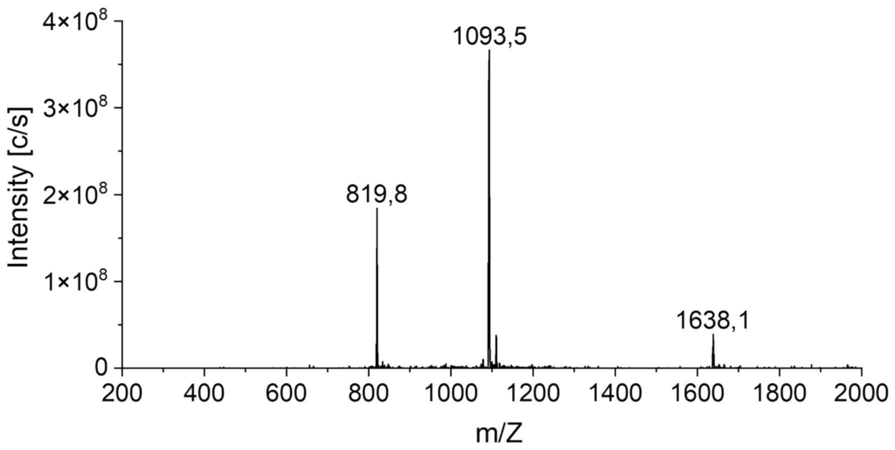

Figure S30. ESI-MS spectrum of the quality control of [natLu]Lu-Comb-2. Calc. monoisotopic mass ($C_{138}H_{226}LuN_{37}O_{44}$): 3280.60 g/mol; found: m/z = 819.8 $[M+4H]^{4+}$, 1093.5 $[M+3H]^{3+}$, 1638.1 $[M+2H]^{2+}$.

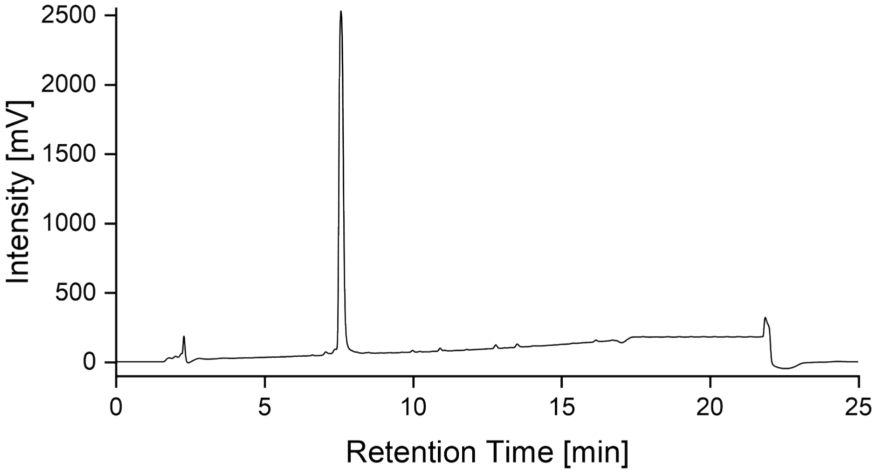

Figure S31. RP-HPLC of the quality control of FITC-TPP-2. With a gradient of 10-90% B in 15 min, the retention time observed was t_R = 7.6 min and the purity: 96%.

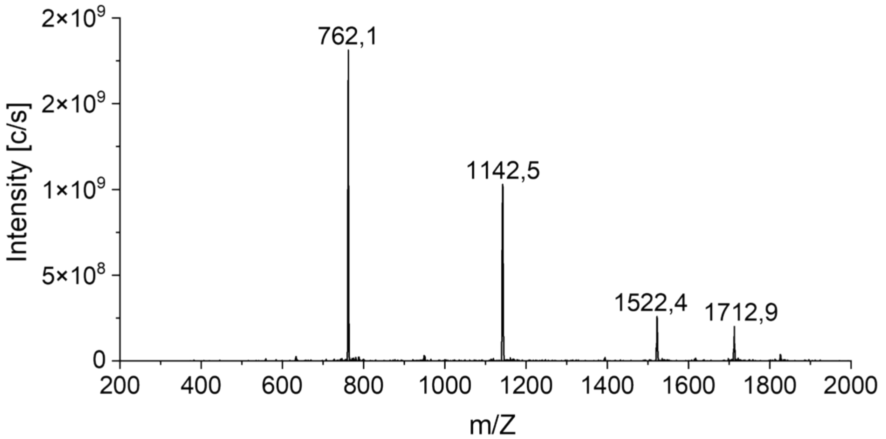

Figure S32. ESI-MS spectrum of the quality control of FITC-TPP-2. Calc. monoisotopic mass ($C_{103}H_{150}N_{22}O_{35}S$): 2287.04 g/mol; found: m/z = 762.1 $[M+3H]^{3+}$, 1142.5 $[M+2H]^{2+}$, 1522.4 $[2M+3H]^{3+}$, 1712.9 $[3M+4H]^{4+}$.

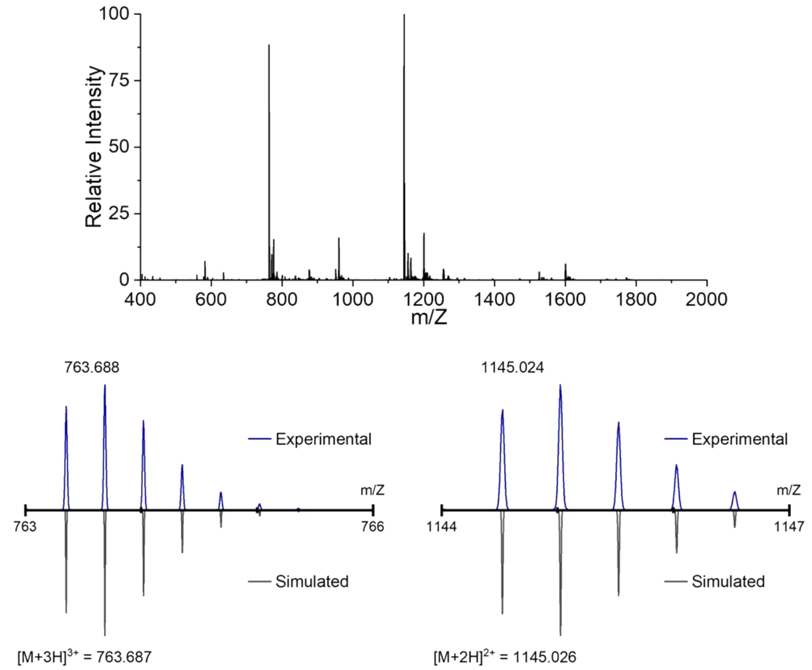

Figure S33. HR-ESI-MS of FITC-TPP-2.

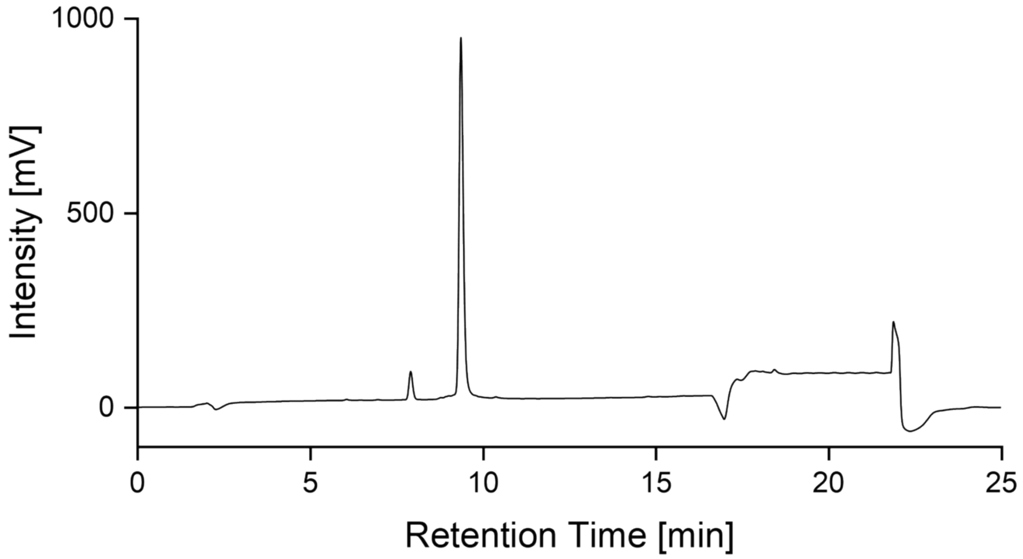

Figure S34. RP-HPLC of the quality control of FITC-Comb-2. With a gradient of 20-60% B in 15 min, the retention time observed was t_R = 9.4 min and the purity: 95%.

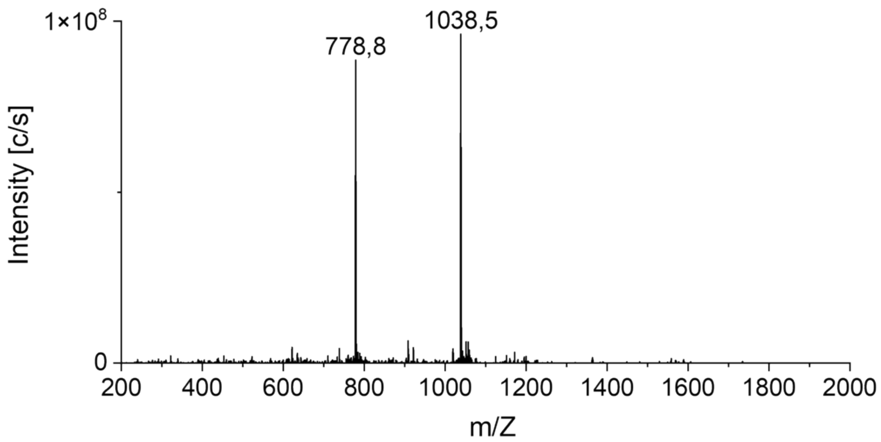

Figure S35. ESI-MS spectrum of the quality control of FITC-Comb-2. Calc. monoisotopic mass ($C_{143}H_{214}N_{34}O_{42}S$): 3111.54 g/mol; found: m/z = 778.8 $[M+4H]^{4+}$, 1038.5 $[M+3H]^{3+}$.

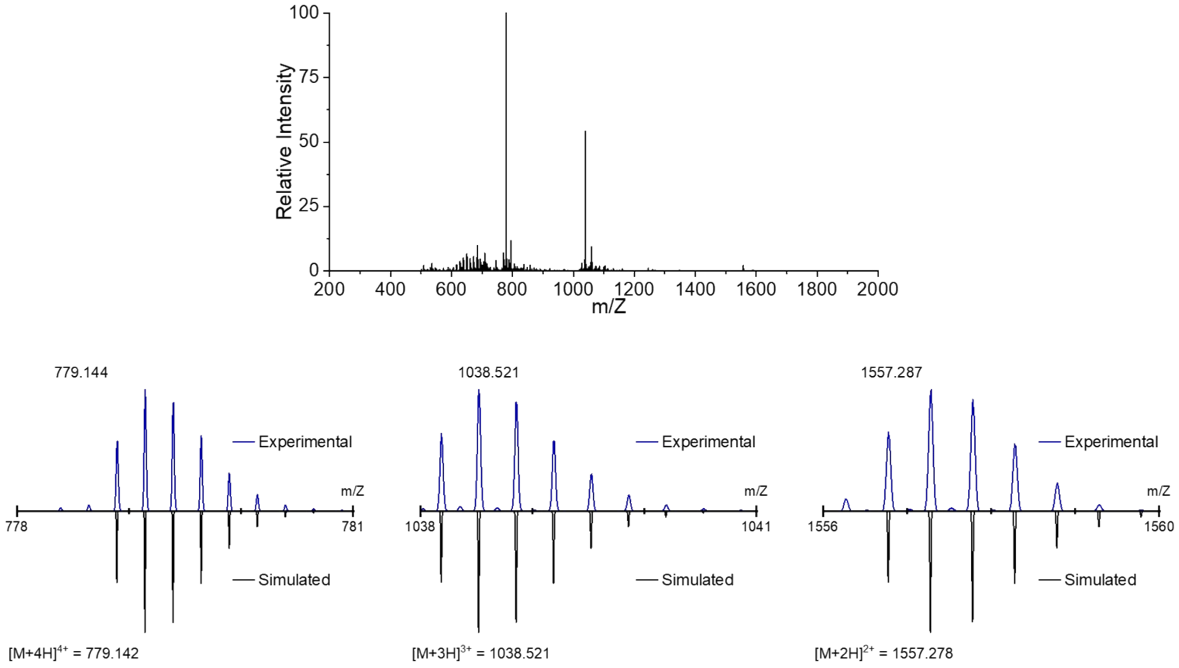

Figure S36. HR-ESI-MS of FITC-Comb-2.

Radio-RP-HPLC and Radio-TLC Chromatograms of Radioactive Labeling

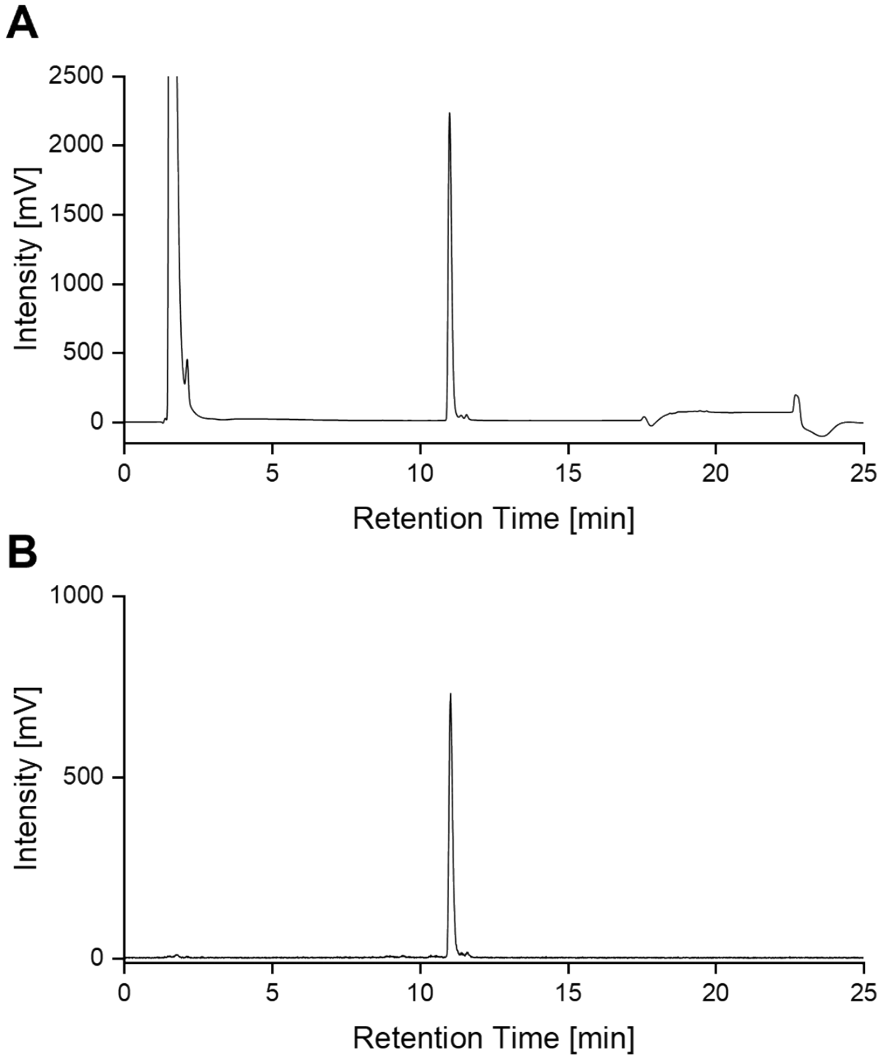

Figure S37. Radio RP-HPLC A: UV- and B: HERM-chromatogram of the coinjection of [^{nat}Lu]Lu-PepH3-1 and [^{177}Lu]Lu-PepH3-1. With a gradient of 10-40% B in 15 min, the retention time observed was t_R = 11.0 min for [^{nat}Lu]Lu-PepH3-1 and t_R = 11.1 min for [^{177}Lu]Lu-PepH3-1 with an RCP of 96%.

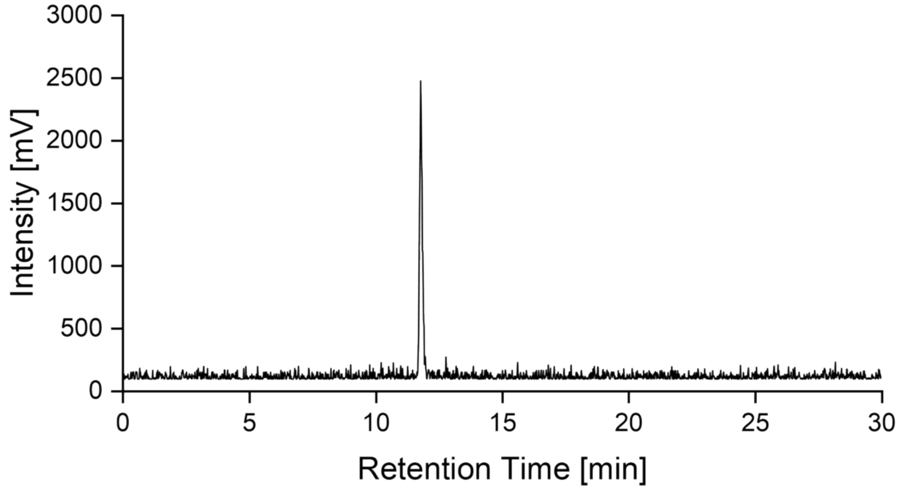

Figure S38. Radio RP-HPLC HERM-chromatogram of [^{67}Ga]Ga-PepH3-1. With a gradient of 10% MeOH for 5 min, 10-100% MeOH in 20 min, the retention time observed was t_R = 11.8 min with an RCP >99%.

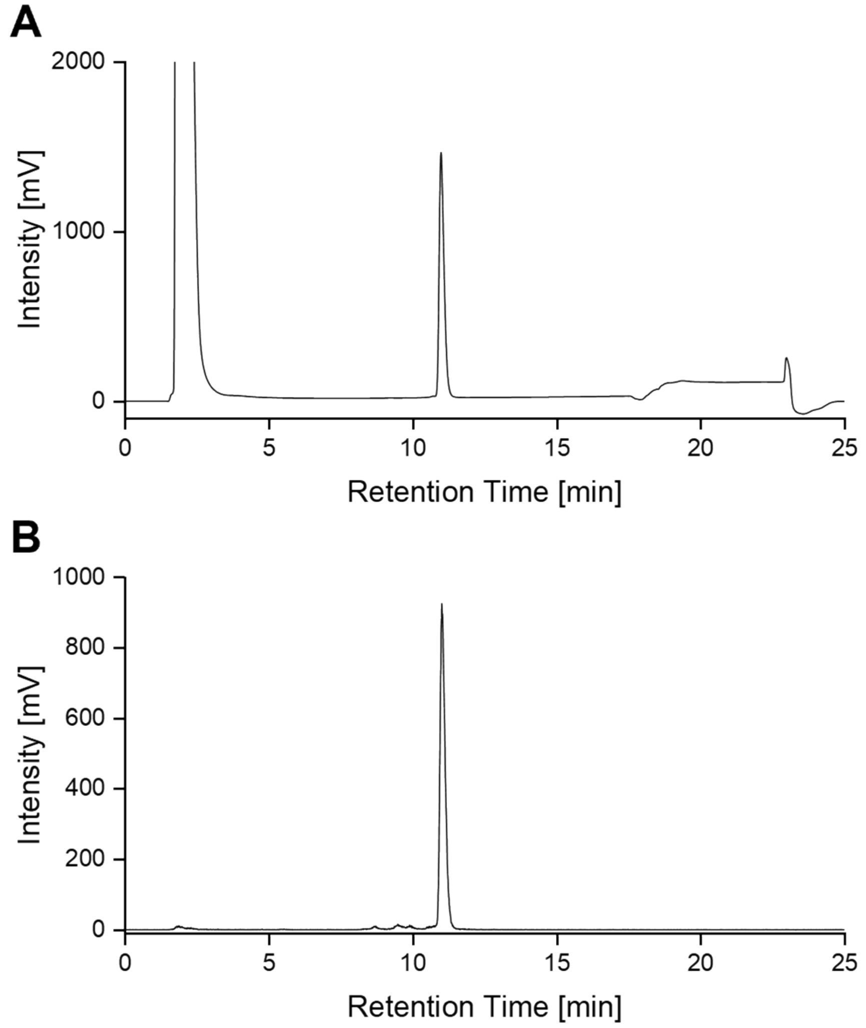

Figure S39. Radio RP-HPLC A: UV- and B: HERM-chromatogram of the coinjection of [^{nat}Lu]Lu-PepH3-2 and [^{177}Lu]Lu-PepH3-2. With a gradient of 10-50% B in 15 min, the retention time observed was t_R = 11.0 min for both [^{nat}Lu]Lu-PepH3-2 and [^{177}Lu]Lu-PepH3-2 with an RCP of 96%.

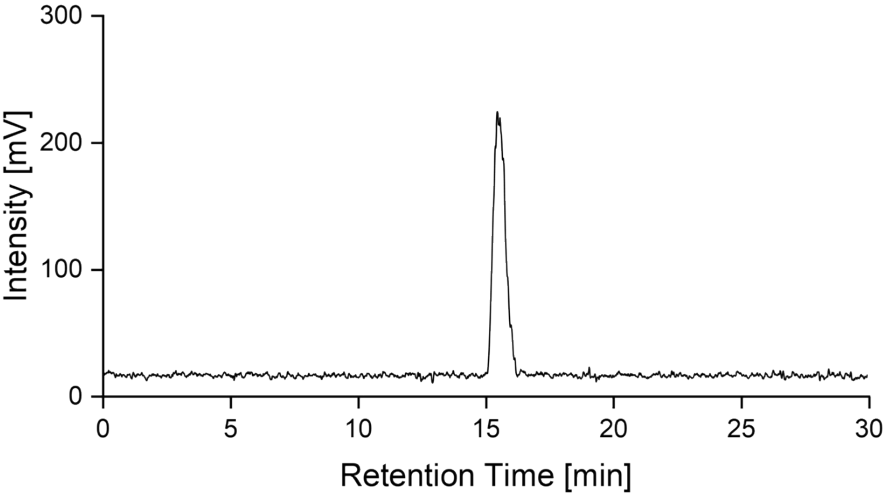

Figure S40. Radio RP-HPLC HERM-chromatogram of [^{67}Ga]Ga-PepH3-2. With a gradient of 10% MeOH for 5 min, 10-100% MeOH in 20 min, the retention time observed was t_R = 15.5 min with an RCP >99%.

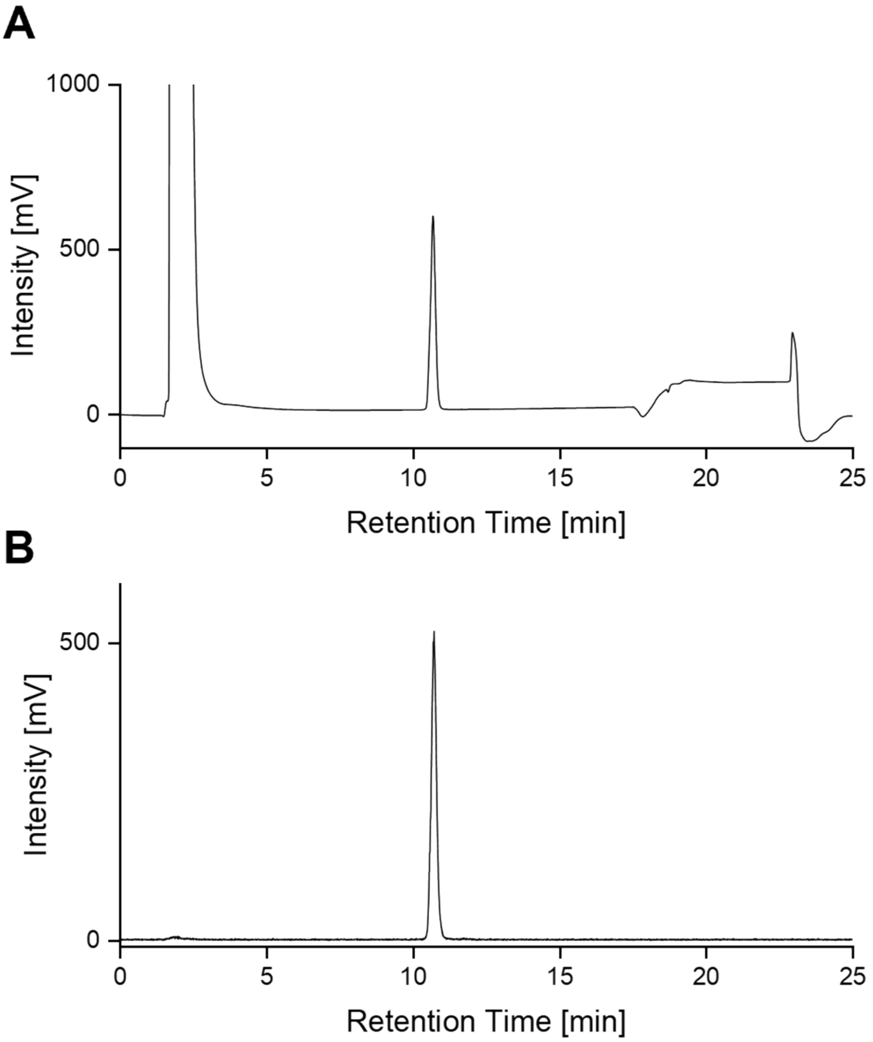

Figure S41. Radio RP-HPLC A: UV- and B: HERM-chromatogram of the coinjection of [^{nat}Lu]Lu-TPP-1 and [^{177}Lu]Lu-TPP-1. With a gradient of 10-50% B in 15 min, the retention time observed was t_R = 10.7 min for both [^{nat}Lu]Lu-TPP-1 and [^{177}Lu]Lu-TPP-1 with an RCP of 98%.

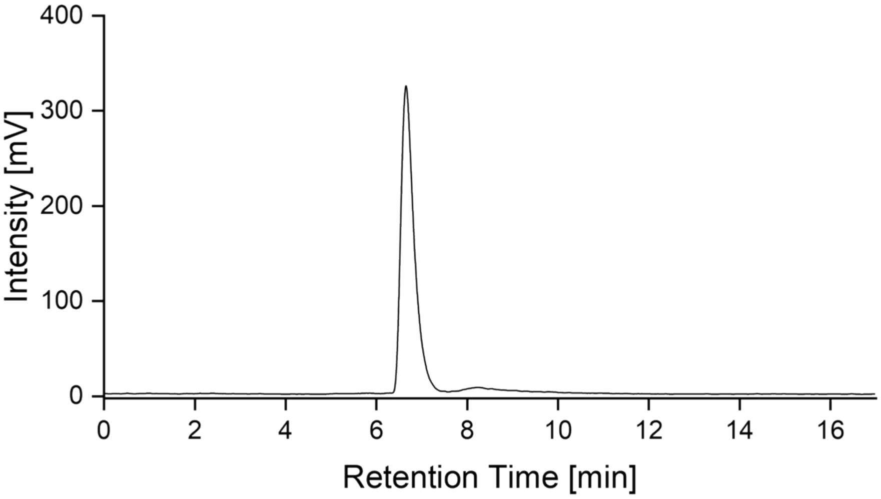

Figure S42. Radio RP-HPLC HERM-chromatogram of [^{67}Ga]Ga-TPP-1. With a gradient of 10-90% B in 10 min, the retention time observed was t_R = 6.7 min with an RCP of 97%.

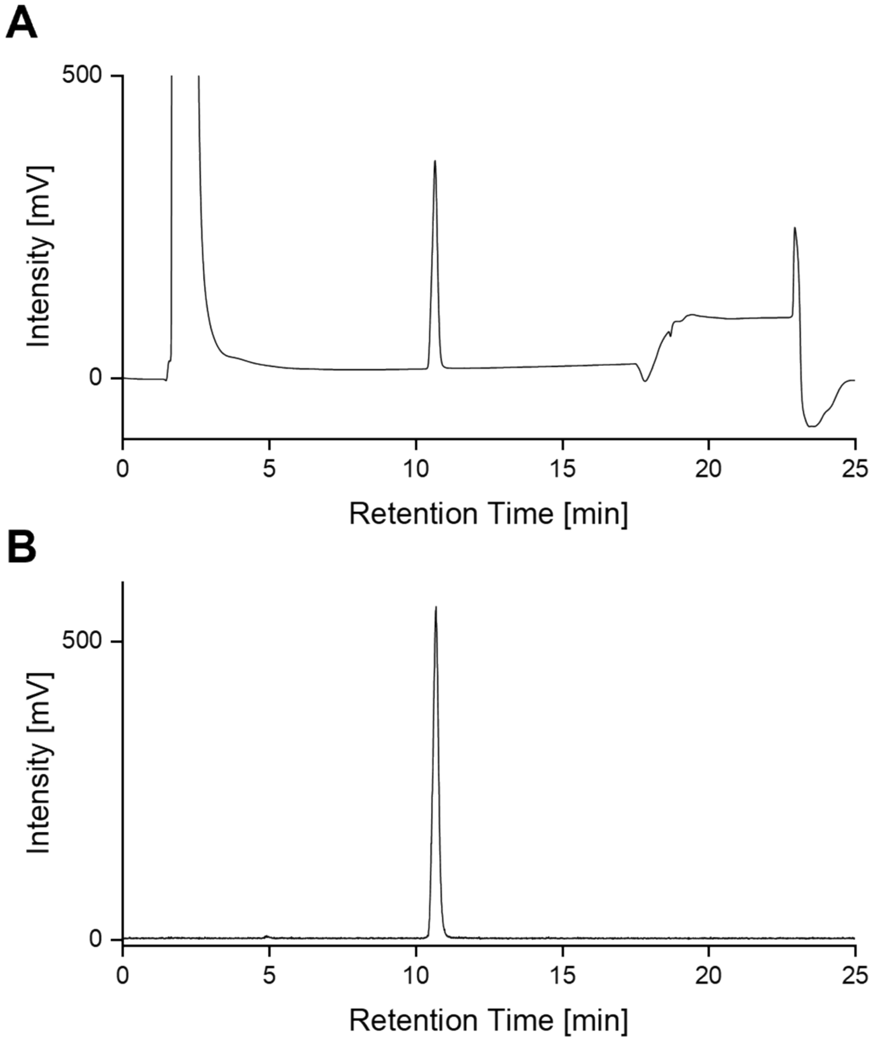

Figure S43. Radio RP-HPLC A: UV- and B: HERM-chromatogram of the coinjection of [^{nat}Lu]Lu-TPP-2 and [^{177}Lu]Lu-TPP-2. With a gradient of 10-50% B in 15 min, the retention time observed was t_R = 10.7 min for both [^{nat}Lu]Lu-TPP-1 and [^{177}Lu]Lu-TPP-1 with an RCP of >99%.

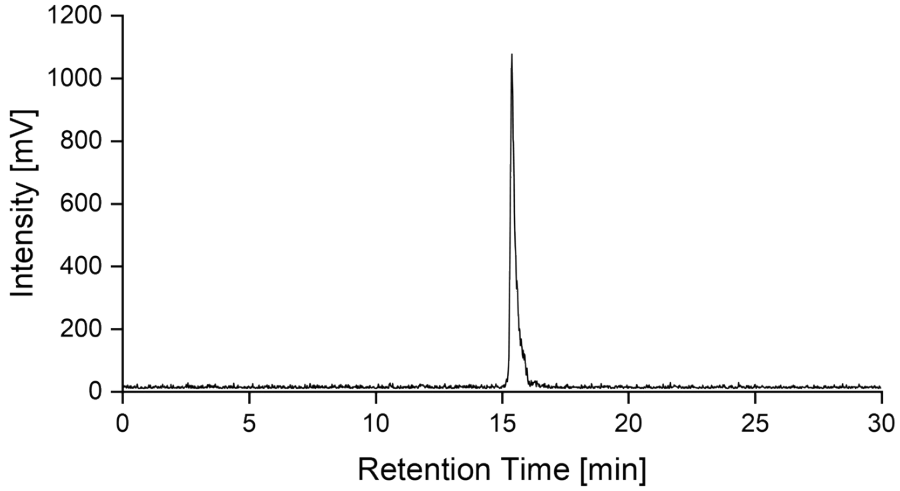

Figure S44. Radio RP-HPLC HERM-chromatogram of [^{67}Ga]Ga-TPP-2. With a gradient of 10% MeOH for 5 min, 10-100% MeOH in 20 min, the retention time observed was t_R = 15.4 min with an RCP 98%.

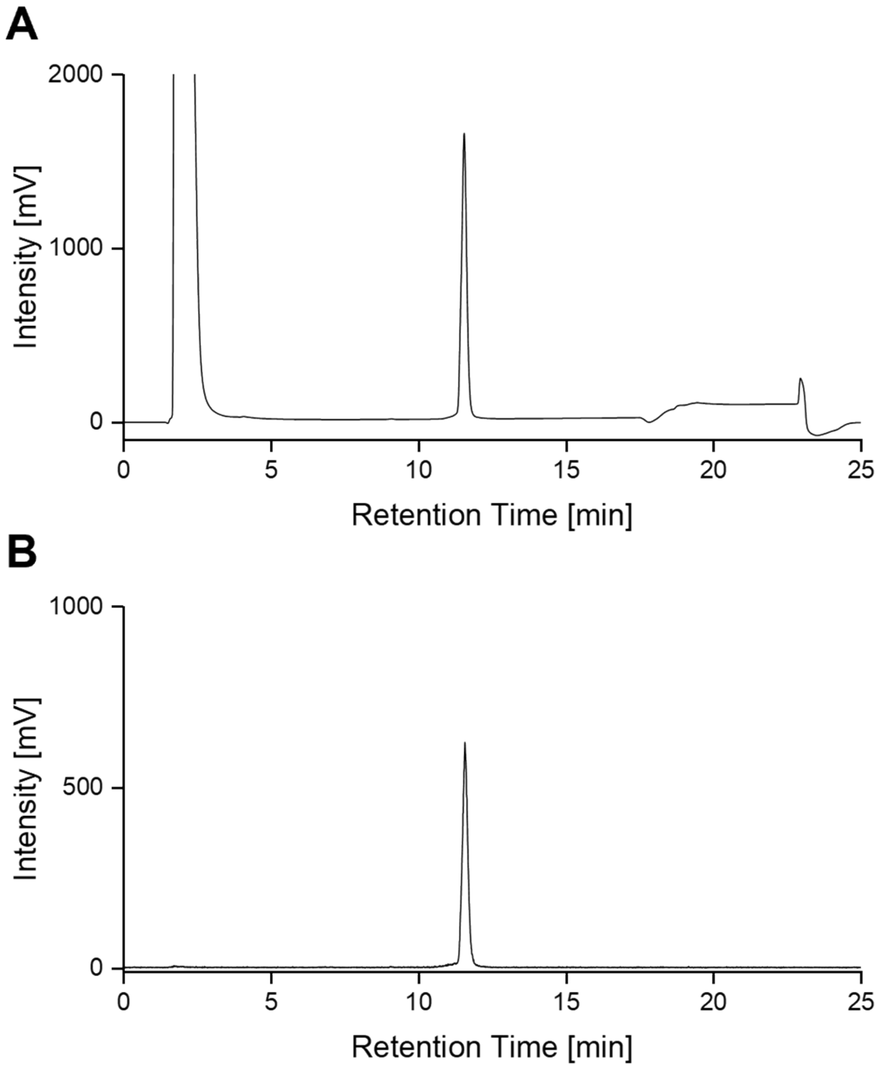

Figure S45. Radio RP-HPLC A: UV- and B: HERM-chromatogram of the coinjection of [^{nat}Lu]Lu-Comb-1 and [^{177}Lu]Lu-Comb-1. With a gradient of 10-50% B in 15 min, the retention time observed was t_R = 11.5 min for [^{nat}Lu]Lu-Comb-1 and t_R = 11.6 min for [^{177}Lu]Lu-Comb-1 with an RCP of 97%.

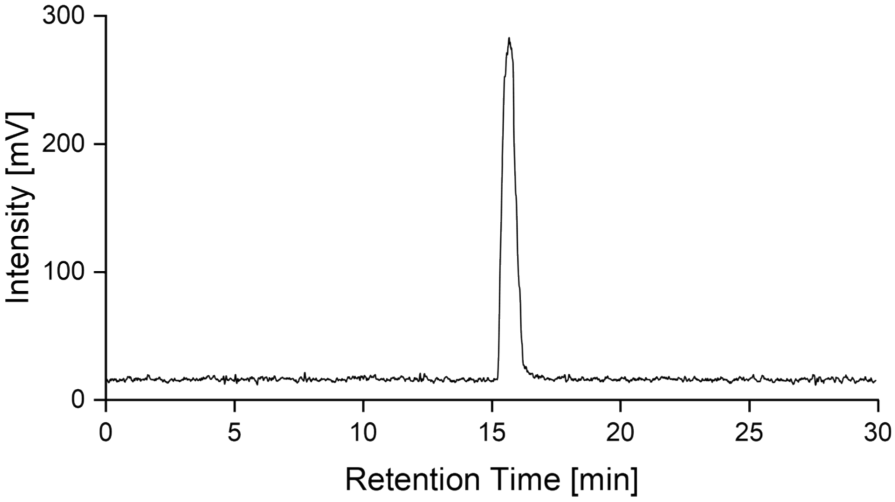

Figure S46. Radio RP-HPLC HERM-chromatogram of [^{67}Ga]Ga-Comb-1. With a gradient of 10% MeOH for 5 min, 10-100% MeOH in 20 min, the retention time observed was t_R = 15.7 min with an RCP >99%.

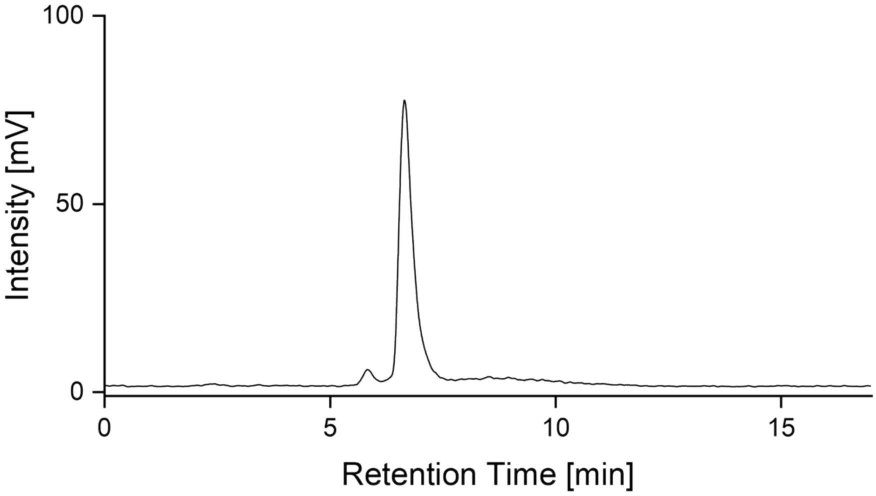

Figure S47. Radio RP-HPLC HERM-chromatogram of [^{68}Ga]Ga-Comb-1. With a gradient of 10-90% B in 10 min, the retention time observed was t_R = 6.7 min with an RCP of 97%.

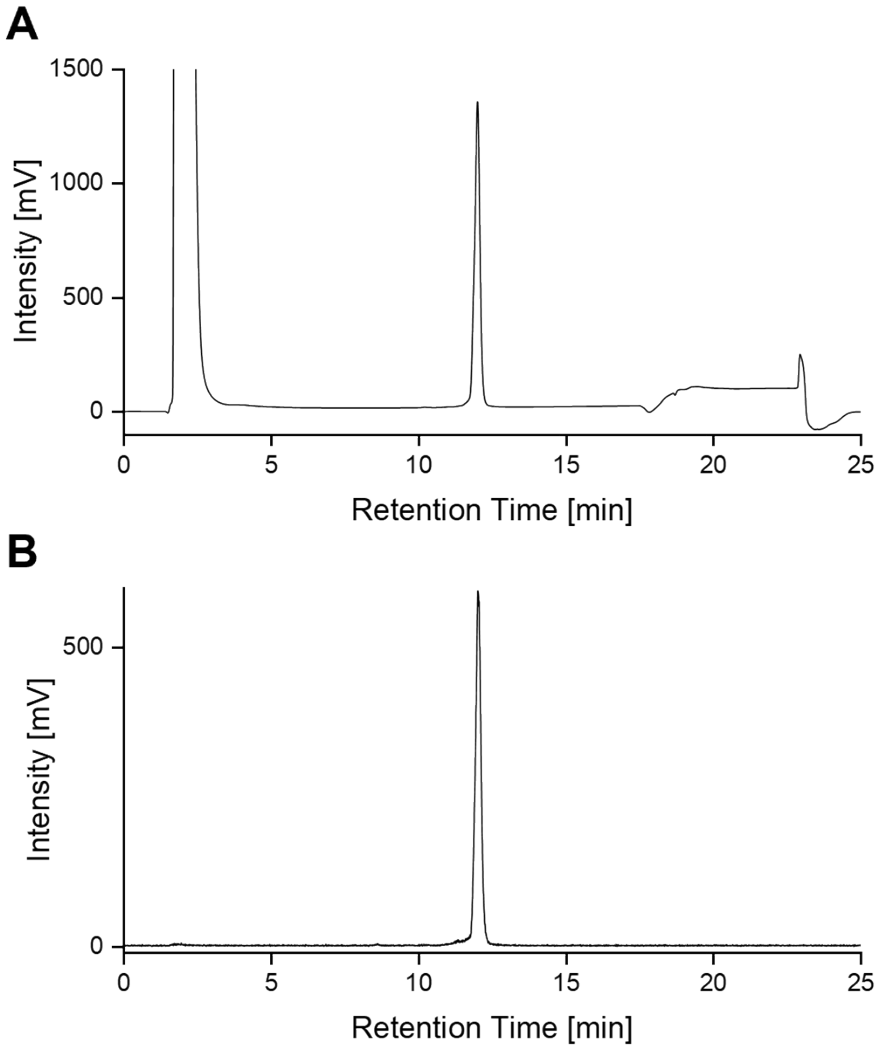

Figure S48. Radio RP-HPLC A: UV- and B: HERM-chromatogram of the coinjection of [^{nat}Lu]Lu-Comb-2 and [^{177}Lu]Lu-Comb-2. With a gradient of 10-50% B in 15 min, the retention time observed was t_R = 12.0 min for both [^{nat}Lu]Lu-Comb-2 and [^{177}Lu]Lu-Comb-2 with an RCP of 97%.

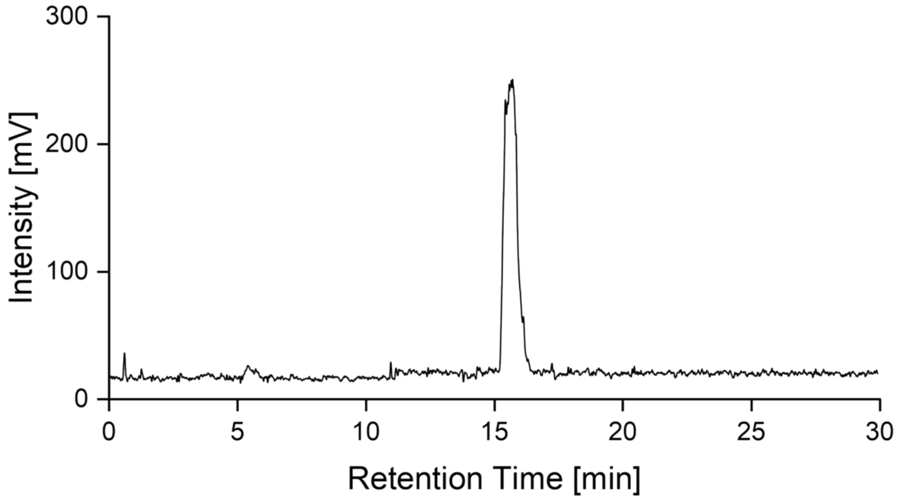

Figure S49. Radio RP-HPLC HERM-chromatogram of [^{67}Ga]Ga-Comb-2. With a gradient of 10% MeOH for 5 min, 10-100% MeOH in 20 min, the retention time observed was t_R = 15.8 min with an RCP 97%.

In Silico Prediction of BBB-Translocation

Table 1. *In silico* prediction of BBB-translocation.

**Peptide**	**Sequence**	**Translocation score (cross)**
PepH3_1	AGILKRWW	0.85
PepH3_2	(PEG6)-AGILKRWW	0.82
TPP_1	TKDNNLLGRFELSG	0.30
TPP_2	(PEG6)-TKDNNLLGRFELSG	0.28
Combo_1	AGILKRWWTKDNNLLGRFELSG	0.46
Combo_2	(PEG6)-AGILKRWWTKDNNLLGRFELSG	0.42
Translocation score: High (cross>0.80); moderate (0.50>cross>0.80); low (cross<0.50)		

Stability Studies

Table S2. Percentages of intact compound during stability study in DMEM GlutaMAX™, calculated based on integration of radio RP-HPLC signals.

Compound	QC [%]	10 min [%]	1 h [%]	2 h [%]	24 h [%]
[^{177}Lu]Lu-PepH3-1	95.2	94.8	93.7	92.9	90.3
[^{177}Lu]Lu-PepH3-2	95.1	92.6	91.2	91.1	91.0
[^{177}Lu]Lu-TPP-1	98.0	97.8	97.7	95.4	95.1
[^{177}Lu]Lu-TPP-2	98.7	98.0	97.2	96.8	96.0
[^{177}Lu]Lu-Comb-1	98.1	98.0	98.0	98.0	98.0
[^{177}Lu]Lu-Comb-2	98.3	98.2	98.2	98.1	97.8

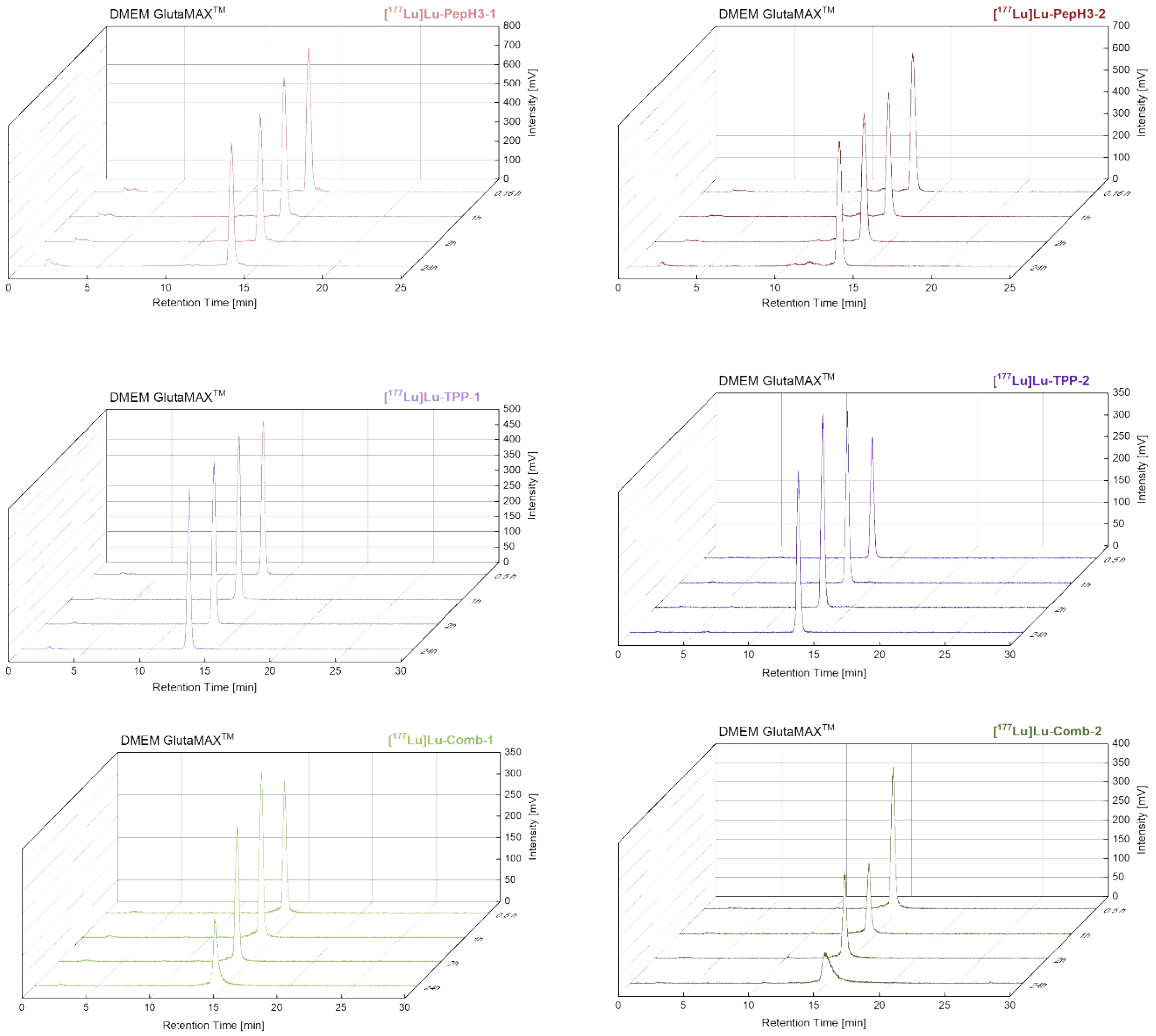

Figure S50. RP-HPLC chromatograms of the stability studies of the various ^{177}Lu labelled tracers in DMEM GlutaMAX™.

Table S3. Percentages of intact compound during stability study in human serum, calculated based on integration of radio RP-HPLC signals.

Compound	QC [%]	10 min [%]	1 h [%]	24 h [%]
[^{177}Lu]Lu-PepH3-1	91.7	90.9	82.7	81.5
[^{177}Lu]Lu-PepH3-2	96.5	96.3	96.3	95.3
[^{177}Lu]Lu-TPP-1	95.5	93.2	69.7	0
[^{177}Lu]Lu-TPP-2	98.5	93.9	70.7	0
[^{177}Lu]Lu-Comb-1	95.9	93.8	69.3	0
[^{177}Lu]Lu-Comb-2	95.2	93.0	85.8	6.1

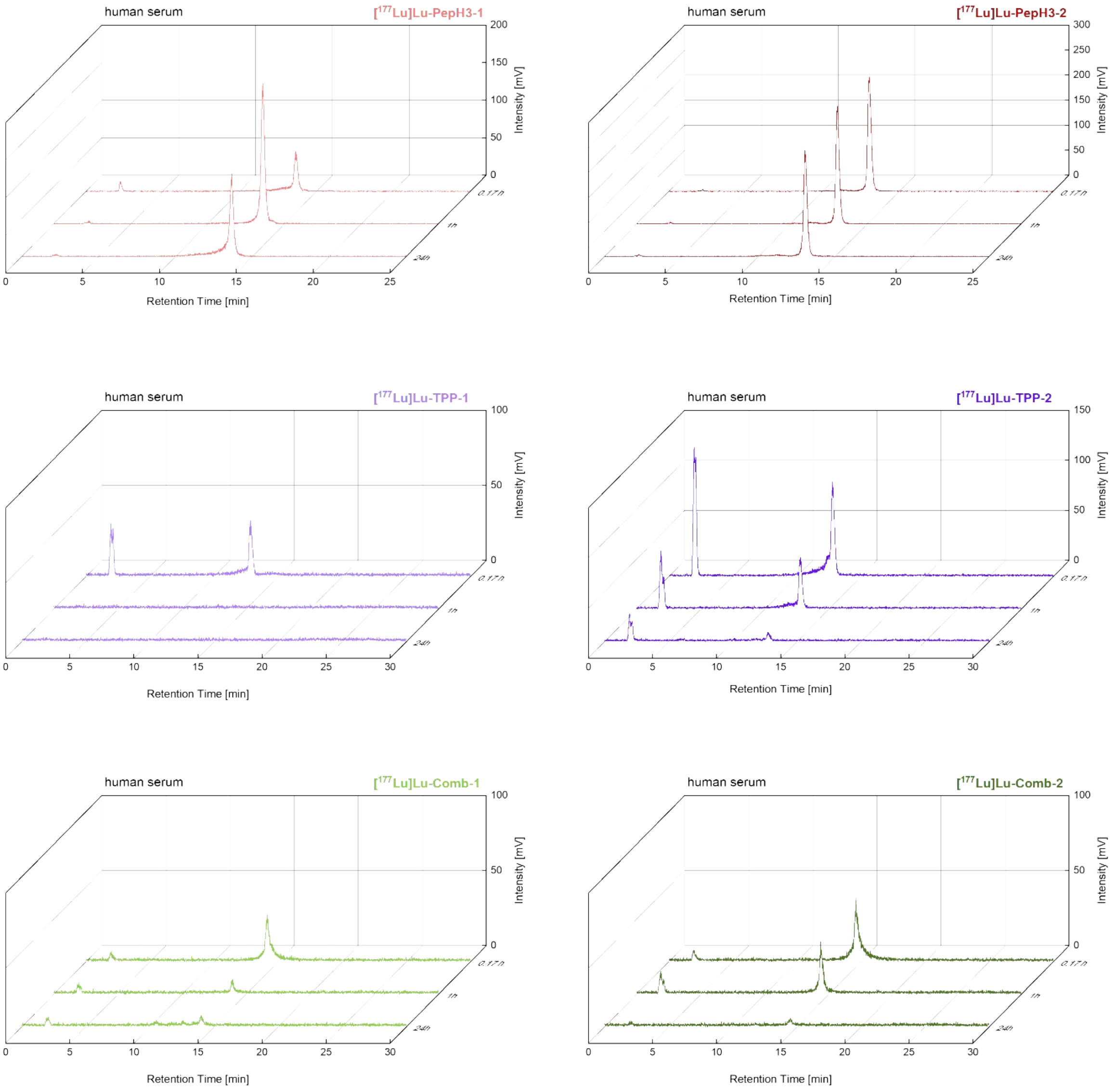

Figure S51. RP-HPLC chromatograms of the stability studies of the various ^{177}Lu-labelled tracers in human serum.

Flow Cytometry Analysis

Original cytometry data files have been provided on Zenodo, doi: 10.5281/zenodo.20183561.

Table S4. Percentage of positive U87-MG cells according to flow cytometry analysis.

Compound	30 min					60 min		
	50 µg/mL (n=3)	1 µM (n=1)	5 µM (n=3)	8 µM (n=3)	24 µM	5 µM	8 µM	24 µM
**Isotype**	11.2±2.5							
**FITC-cmHsp70.1**	28.7±6.0							
**FITC-TPP-2**		12.5	46.2±2.5	71.6±8.0	98.8	77.3	95.9	100
**FITC-Comb-2**		23.7	77.5±4.4	92.7±0.1	98.8	59.8	94.3	100

Table S5. Percentage of positive bEnd.3 cells according to flow cytometry analysis.

Compound	60 min
	50 µg/mL (n=1)
**Isotype (AF488)**	0.2
**FITC-cmHsp70.1**	16.9

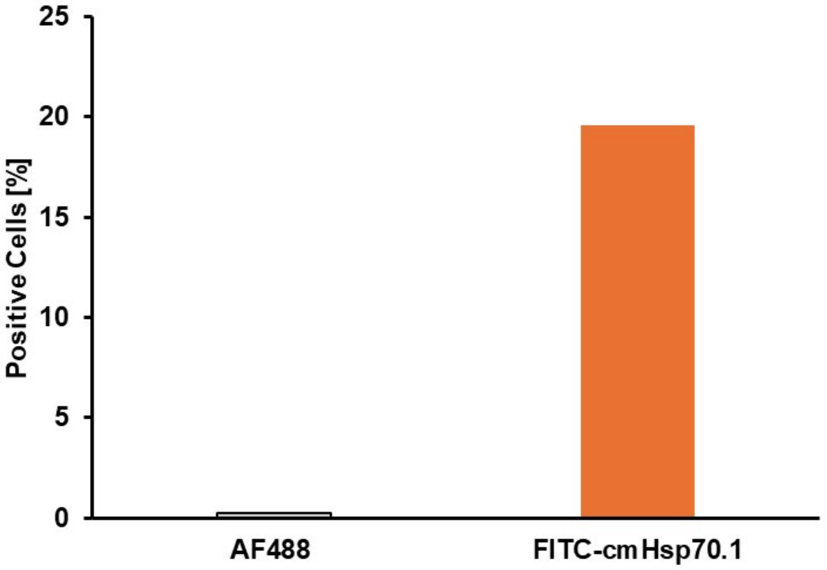

Figure S52. Cytometry analysis of mHsp70 expression in murine brain endothelial cells (b.End3) cells with antibody FITC-cmHsp70.1 (50 µg/mL). AF488 (50 µg/mL) was used as a negative isotype control.

Biodistribution and Metabolic Studies

Table S6. Biodistribution results 2 min p.i. in %IA/g of the ^{67}Ga-labeled compounds in naive CD1 mice (n =3).

**Organ**	**[^{67}Ga]Ga-PepH3-1**	**[^{67}Ga]Ga-PepH3-2**	**[^{67}Ga]Ga-Comb-1**	**[^{67}Ga]Ga-Comb-2**	**[^{67}Ga]Ga-TPP-2**	**[^{67}Ga]Ga-Comb-2 (perfusion)**
Blood	8.46±0.67	12.29±2.77	8.65±0.22	7.74±2.29	9.68±2.05	10.21±0.41
Heart	2.59±0.56	3.27±0.36	2.93±0.62	2.86±0.82	2.48±0.16	1.44±0.09
Lung	5.97±0.97	6.02±0.42	5.59±1.37	4.53±0.81	4.88±0.94	1.57±0.76
Liver	4.44±2.10	4.04±0.35	3.34±0.28	2.85±0.82	3.30±0.87	1.45±0.19
Stomach	1.31±0.30	1.56±0.04	0.90±0.13	1.18±0.26	1.05±0.26	0.86±0.19
Pancreas	1.55±0.11	2.16±0.35	1.71±0.16	1.57±0.15	1.40±0.05	1.27±0.16
Spleen	2.13±0.66	2.19±0.25	2.21±0.25	1.32±0.26	1.04±0.18	1.64±0.10
Intestine	1.61±0.30	1.90±0.22	1.49±0.09	1.53±0.27	2.00±0.59	1.55±0.10
Kidney	21.97±5.84	23.02±1.74	64.16±2.37	60.31±5.71	27.64±3.70	8.12±0.43
Muscle	2.30±0.20	2.59±0.17	1.67±0.39	2.23±0.23	2.21±0.16	2.16±0.23
Bone	2.34±0.20	2.91±0.44	2.90±0.44	2.40±0.74	2.67±0.18	2.43±0.16
**Brain**	**0.42±0.08**	**0.37±0.05**	**0.42±0.15**	**0.60±0.17**	**0.56±0.10**	**0.14±0.06**

Table S7. Biodistribution results 60 min p.i. in %IA/g of the ^{67}Ga-labeled compounds in naive CD1 mice (n = 3).

**Organ**	**[^{67}Ga]Ga-PepH3_1**	**[^{67}Ga]Ga-PepH3_2**	**[^{67}Ga]Ga-Comb_1**	**[^{67}Ga]Ga-Comb_2**	**[^{67}Ga]Ga-TPP_2**
Blood	0.34±0.13	0.11±0.06	0.29±0.07	0.27±0.09	0.22±0.12
Heart	0.12±0.04	0.04±0.01	0.10±0.04	0.09±0.03	0.05±0.03
Lung	0.84±0.38	0.13±0.02	0.23±0.05	0.24±0.03	0.15±0.04
Liver	4.86±1.01	1.28±0.21	0.81±0.49	0.63±0.16	0.33±0.18
Stomach	0.18±0.11	0.05±0.01	0.12±0.10	0.18±0.12	0.12±0.05
Pancreas	0.07±0.02	0.04±0.01	0.09±0.04	0.17±0.08	0.04±0.03
Spleen	2.42±1.20	0.24±0.02	0.22±0.13	0.30±0.09	0.15±0.08
Intestine	0.18±0.07	0.08±0.00	0.14±0.07	0.23±0.10	0.14±0.09
Kidney	5.30±0.66	12.32±1.31	91.88±20.63	110.90±16.80	7.98±1.92
Muscle	0.12±0.02	0.05±0.02	0.11±0.04	0.11±0.06	0.05±0.03
Bone	0.35±0.27	0.11±0.02	0.16±0.06	0.19±0.09	0.07±0.03
**Brain**	**0.03±0.01**	**0.01±0.00**	**0.02±0.01**	**0.03±0.02**	**0.01±0.00**

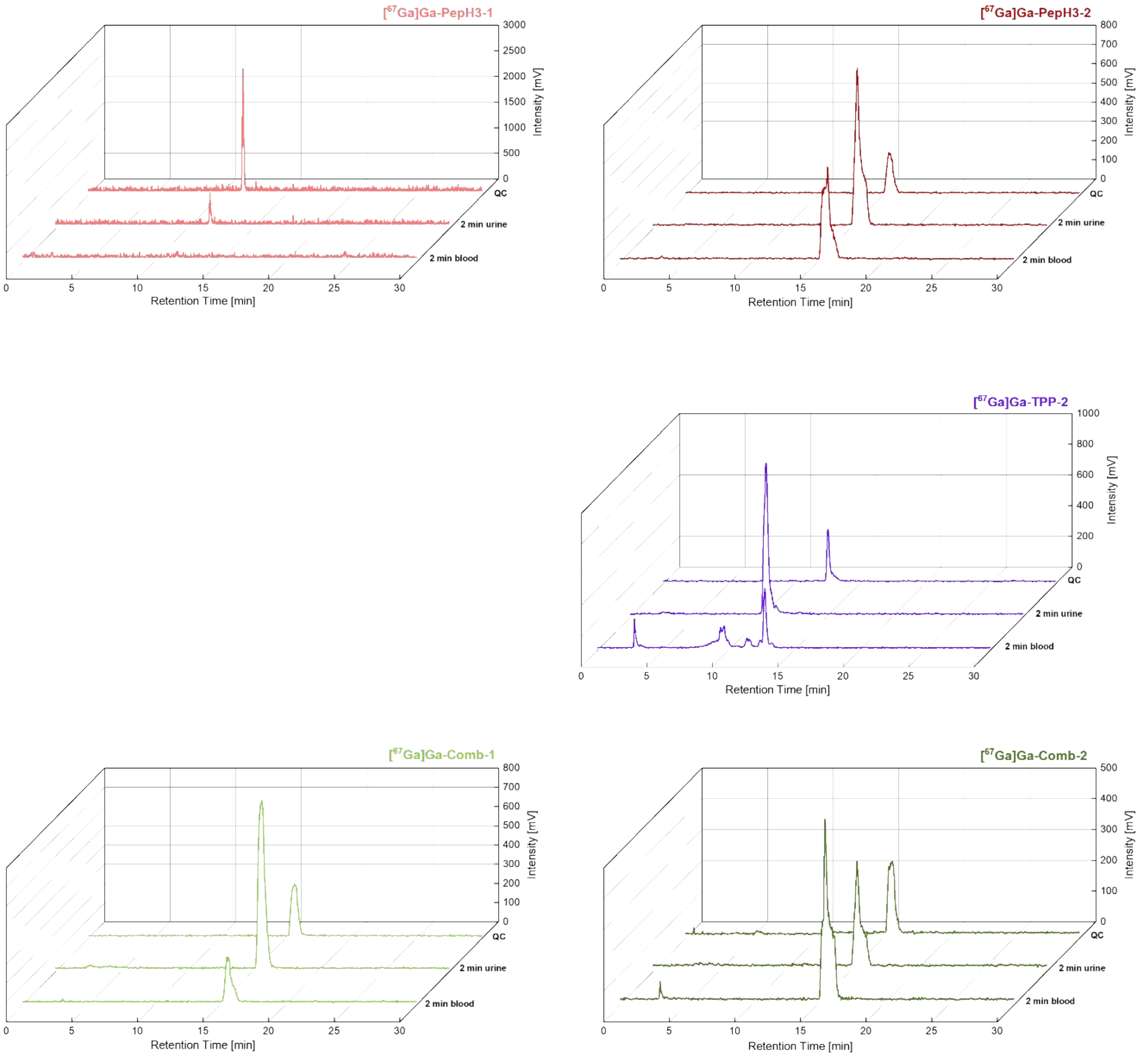

Figure S53. Stability studies in murine urine and blood were performed after the biodistribution study. Radio RP-HPLC chromatograms from back to front: Quality control (QC), urine 2 min p.i., blood 2 min p.i. Gradient: 10% MeOH for 5 min, 10-100% MeOH for 20 min, 100% MeOH for 3 min, 100-10% MeOH for 2 min.
